# Synthesis of 9-Hydroxy-1*H*-Benzo[*f*]chromene Derivatives with Effective Cytotoxic Activity on MCF7/ADR, *P*-Glycoprotein Inhibitors, Cell Cycle Arrest and Apoptosis Effects

**DOI:** 10.3390/ijms24010049

**Published:** 2022-12-20

**Authors:** Fawzia F. Albalawi, Mohammed A. A. El-Nassag, Raafat A. El-Eisawy, Mahmoud Basseem I. Mohamed, Ahmed M. Fouda, Tarek H. Afifi, Ahmed A. Elhenawy, Ahmed Mora, Ahmed M. El-Agrody, Heba K. A. El-Mawgoud

**Affiliations:** 1 Chemistry Department, Faculty of Science, Taibah University, Al-Madinah Al-Munawarah 30002, Saudi Arabia; 2 Chemistry Department, Faculty of Science, Al-Azhar University, Cairo 11884, Egypt; 3 Chemistry Department, Faculty of Science and Art, Al-Baha University, Al-Baha 65582, Saudi Arabia; 4 Chemistry Department, Faculty of Science, King Khalid University, Abha 61413, Saudi Arabia; 5 Chemistry Department, Faculty of Science and Art, Albaha University, Albahah 65731, Saudi Arabia; 6 Chemistry Department, Faculty of Women for Arts, Science, and Education, Ain Shams University, Cairo 11757, Egypt

**Keywords:** 1*H*-Benzo[*f*]chromenes, cytotoxicity, MCF-7/ADR, *P*-gp inhibitors, cell cycle arrest, SAR study

## Abstract

*β*-Enaminonitriles bearing 9-hydroxy-1*H*-benzo[*f*]chromene moiety was synthesized. The targeted compounds were evaluated for their anti-proliferative activity against three human tumor cell lines, PC-3, SKOV-3 and HeLa, and the active cytotoxic compounds were further evaluated against cancer cells, MCF-7/ADR, and two normal cell lines, HFL-1 and WI-38. Few compounds were assigned to be the most potent derivatives against PC-3, SKOV-3 and HeLa cell lines in comparison with Vinblastine and Doxorubicin. Several compounds possessed a relatively good potency against MCF-7/ADR cells as compared with Doxorubicin and were tested as a *P*-gp inhibitor. Moreover, the halogenated substituents, 2,4-F_2_, 2,3-Cl_2_, 2,5-Cl_2_ and 3,4-Cl_2;_ have good potency against *P*-gp-mediated MDR in MCF-7/ADR as compared with Doxorubicin. Meanwhile, Rho123 accumulation assays revealed that few compounds effectively inhibited *P*-pg and efflux function. In addition, certain derivatives induced apoptosis and an accumulation of the treated MCF-7/ADR cells in the G1, S and G1/S phases.

## 1. Introduction

Among the most significant heterocyclics are the substituted chromenes and benzochromenes, owing to their antimicrobial [1,2,3,4,5], antiviral, anti-HIV, antileishmanial, anti-anaphylactic [6,7,8], anticancer [9,10,11,12,13,14,15,16], anti-inflammatory [17,18] and antioxidant [19] applications. *β*-enaminonitriles containing 4*H*-benzo[*h*]chromene systems have been used in cancer treatment approaches; therefore, they are described as prospective cancer pharmaceuticals’ targets. Examples include 2-amino/acetylamino substituents of 6-chloro/methoxy-4*H*-benzo[*h*]chromenes that exhibited cytotoxic effects against MCF-7, HCT-116 and HepG-2 [9,20,21], as well as 2-amino and 2,7-diamino derivatives that showed anti-proliferative and *c*-Src kinase inhibitory effects [14,22]. Additionally, 1*H*-benzo[*f*]chromene derivatives are advantageous for the creation of strong anticancer agents. For instance, 1*H*-benzo[*f*]chromenes with 8-bromo/methoxy and 1H-tetrazol-5-yl substituents block *c*-Src kinase inhibitory activity with anticancer characteristics [23,24]. Apoptosis and cell cycle arrest in human cancer cells are brought on by the 8/9-bromo substituents of 1*H*-benzo[*f*]chromenes, which inhibit topoisomerase I and II [9,10]. The 3-amino substituent of 1*H*-benzo[*f*]chromenes also exhibits anti-proliferative properties, and DNA binding also functions as a Bcl-2 protein inhibitor [25]. Meanwhile, fused benzochromenes are also valuable as effective anticancer agents; for instance, the 9-amino/benzylideneamino and 8-amino/imino derivatives of benzochromenopyrimidine and benzochromenopyrimidin-8-one, respectively, are found to be effective against caspase 3/7 activators and targets for cell cycle study [5,11]. Furthermore, the cell cycle was arrested in the 9-amino/methyl derivative of 7-(2,4/3,4-dimethoxyphenyl)benzochromenopyrimidine, responsible for apoptosis, caspase 3/7 activation, DNA breakage, cell invasion and migration [13,14].

Overexpression of ABC transporters such as *P*-glycoprotein (P-gp), multidrug resistance protein 1 (MDR1) and ATP-binding cassette subfamily B member 1 (ABCB1) is the main contributor to MDR [26,27]. On the other hand, MDR is contributing to chemotherapy failure [28]. The multidrug-resistant phenotype in cancer that mediates MDR also heavily relies on *P*-glycoprotein (*P*-gp/ABCB1) [29].

**Figure 1:**
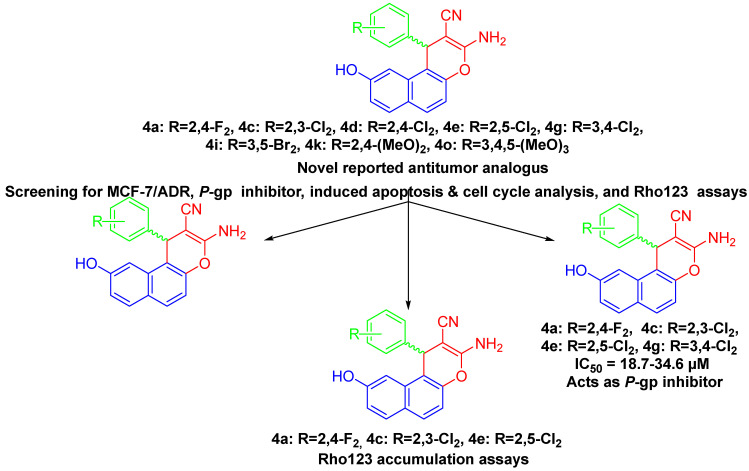
Study of antitumor activities, MCF-7/ADR, P-gp inhibitor, apoptosis, cell cycle analysis, and Rh123 results.

Based on our ongoing experiences to create effective anticancer benzochromene-based medicines, [30,31,32,33,34,35,36,37,38,39,40,41,42,43,44,45,46,47,48], we have initiated the current study. Developing drugs with potential anti-proliferative activity against three cancer cell lines, PC-3, SKOV-3 and HeLa, while having no cytotoxic effect on normal cell lines HFL-1 and WI-38, was the main motivation behind our decision to design and synthesize *β*-enaminonitriles incorporating a 9-hydroxy-1*H*-benzo[*f*]chromene moiety. An additional objective is to target MCF-7/ADR cells; the active 9-hydroxy-1*H*-benzo[*f*]chromene derivatives were investigated as *P*-gp inhibitors utilizing various assays, including Western blot and Rho123 accumulation. The 9-hydroxy-1*H*-benzo[*f*]chromene derivatives **4i**, **4e**, **4d**, **4a**, **4g** and **4c**, respectively, have good potency against MCF-7/ADR cells based on screening results. Additionally, Rho123 accumulation experiments revealed that **4a**, **4c** and **4e** efficiently inhibited *P*-pg and efflux function (Figure 1).

**Figure 2:**
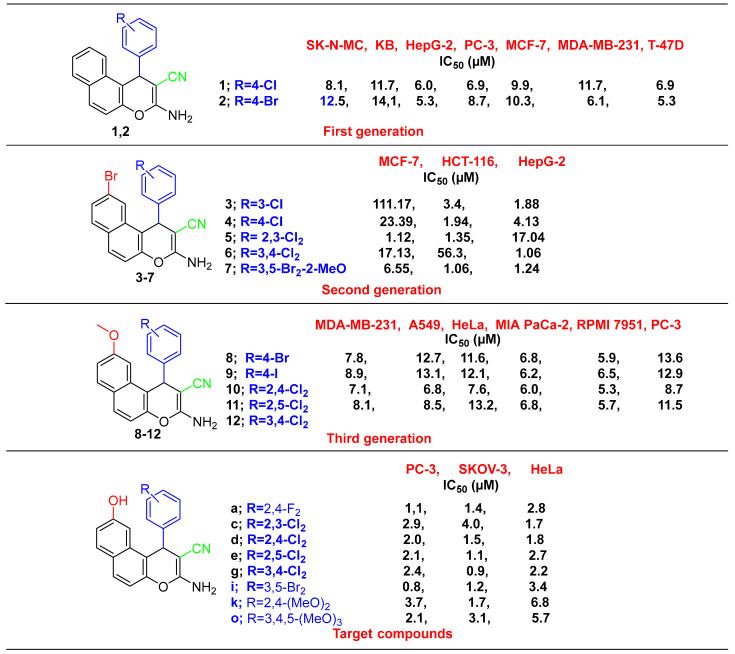
Rationale for designing target benzochromene derivatives and previous studies compounds.

These compounds were found to be effective against *P*-gp inhibitors. Additionally, compounds **4d** and **4e** caused apoptosis and an increase in MCF-7/ADR cells in the G1 phase, whereas compounds **4a**, **4c**, **4g** and **4i** did so in the S phase and G1/S phases, respectively (Figure 1). The type of substituents at the 9-position and the aryl group at the 1-position appears to be critical to the cytotoxic action; therefore, this was well thought out during the rational design of the 9-hydroxy-1*H*-benzo[*f*]chromene derivatives. Figure 2 compares the behaviors of the recently synthesized derivatives linked to a hydroxyl group at position 9 with those previously synthesized with unsubstituted naphthalene moiety: in first generation **1** and **2** [49] is a substituted naphthalene moiety linked to a bromine at position 9, second generation is **3**–**7** [10] and third generation with a methoxy group at position 9 is **8**–**12** [50]. The comparison revealed that the newly synthesized derivatives **4a**, **4c**, **4d**, **4e**, **4g**, **4i**, **4k** and **4o** significantly increased or paralleled the potency of the previously synthesized compounds **1**–**12** when used against cancer cell lines [10,49,50].

## 2. Results and Discussion

### 2.1. Chemistry

**Scheme 1:**
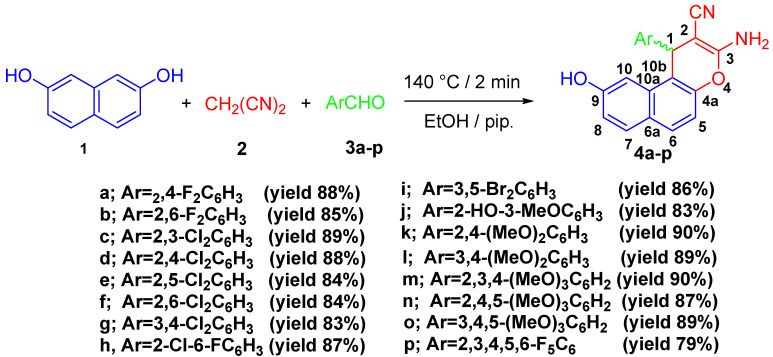
Microwave irradiation synthesis of 3-amino-1-aryl-9-hydroxy-1H-benzo[f]chromene-2-carbonitrile (**4a–p**).

Ethanolic solutions of compounds (3-amino-1-aryl-9-hydroxy-1*H*-benzo[*f*]chromene-2-carbonitrile, **4a–p**) were created by heating naphthalene-2,7-diol (**1**), malononitrile (**2**) and aromatic aldehydes (**3a–p**), and piperidine was kept under microwave irradiation for two minutes at 140 °C (Scheme 1). The best results were obtained using 400 W with a 2 min reaction period to produce the largest yield of compound **4**. This reaction was repeated at different watt powers (200, 300, 400 W) and time intervals (1, 1.5, 2 min) while being monitored using TLC. For the synthesis of **4a–p**, the optimum microwave irradiation conditions were mentioned in Table S1: Supplementary Materials.

### 2.2. Optical Activities 

A Carl Zeiss polarimeter (Jena, Germany.) was used to gauge the target compound 4 which found “optical inactive” in the form of a racemic (±) mixture [51], refer to Scheme 1.

### 2.3. Spectroscopic Data

The structures and purities of the synthesized compounds **4a–p** were confirmed by spectral analysis, i.e., IR, ^1^H NMR, ^13^C NMR and MS spectra. The IR spectra of **4a–p** revealed the presence of peaks around *υ* 3490–3423, 3445–3408, 3353–3222, 3267–3177 cm ^−1^ assigned to the amino and hydroxyl groups and for the CN groups in the region *υ* 2214–2175 cm.^−1^ The 1H NMR spectra of **4a–p** showed the singlet signal of the OH, NH_2_ and CH protons around *δ* 10.77–9.82, 7.27–6.41 and *δ* 5.97–4.96 ppm, respectively. Furthermore, the ^13^ C NMR spectra of **4a–p** showed the signals resonating at *δ* 38.47–26.51 ppm, attributable to the CH carbons. In addition, the MS spectra and the elemental analyses of **4a–p** delivered absolute confirmation for their structures (see Supplementary Materials of the spectral data from Figure S1 to S55).

### 2.4. Biological Evaluation

#### 2.4.1. In Vitro Cytotoxic Activity

**Figure 3:**
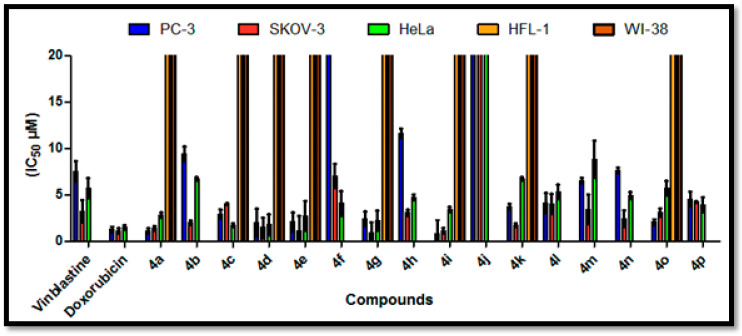
IC_50_ values are expressed in (μM) of the target compounds **4a–p** against PC-3, SKOV-3 and HeLa tumor cells and HFL-1 and WI-38 normal cell lines.

**Figure 4:**
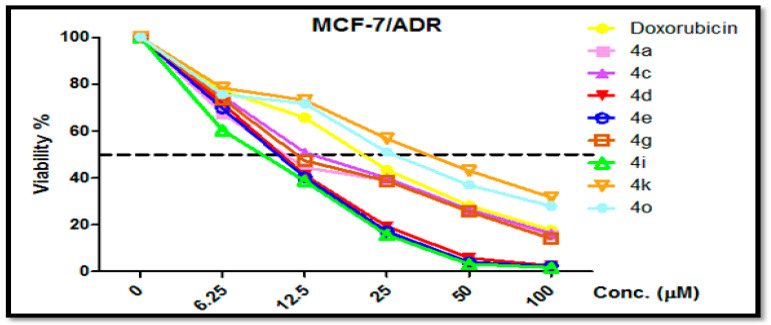
Dose dependent cytotoxicity in MCF-7/ADR cells and the effect of varying concentrations of tested compounds **4a**, **4c**, **4d**, **4e**, **4g**, **4i**, **4k** and **4o** on cell growth of MCF-7/ADR cells following exposure for 24 h.

Three human cancer cell lines, PC-3 (human prostate adenocarcinoma, metastatic), SKOV-3 (ovarian cancer cell line) and HeLa (cervical cancer cell line), were used to test the anti-proliferative activity of *β*-enaminonitrile (**4a–p**) using the MTT colorimetric assay [52] at concentrations ranging from 0–100 μM. The representative active cytotoxic substances **4a**, **4c**, **4d**, **4e**, **4g**, **4i**, **4k** and **4o** were also tested against two normal cell lines, human fetal lung (HFL-1) and human diploid fibroblasts (WI-38), as well as Adriamycin (ADR)-resistant human breast cancer cells (MCF-7/ADR). The reference cytotoxic agents, Vinblastine and Doxorubicin, were used in the tests. The results were expressed as growth inhibitory concentration (IC_50_ μM) values, which reveal the concentrations necessary for 50% inhibition of cell growth after 24 h of incubation compared to the untreated ones (Figure 3 and Figure 4 and Table 1).

Table 1 indicated that the target compounds displayed excellent to modest anti-proliferative activity against PC-3, SKOV-3 and HeLa cell lines. In particular, compounds **4i** and **4a** (IC_50_ = 0.8 ± 0.1 and 1.1 ± 0.4 μM) exhibited the most potent counterparts against PC-3 cells as they were 9.4 and 6.8 times more active than Vinblastine (IC_50_ = 7.5 ± 1.3 μM) and 1.6 and 1.2 times more active than Doxorubicin (IC_50_ = 1.3 ± 0.3 μM), while compounds **4d**, **4e**, **4o**, **4g**, **4c**, **4k**, **4l**, **4p** and **4m** with IC_50_ ranging from 2.0 to 6.5 μM had excellent anti-proliferative activities against PC-3 cells as compared to Vinblastine (IC_50_ = 7.5 ± 1.3 μM).

In addition, compounds **4g**, **4e** and **4i** have excellent anti-proliferative activities against SKOV-3 cells (IC_50_ = 0.9 ± 0.3, 1.1 ± 0.4 and 1.1 ± 0.1 μM, respectively), which are better than the used reference drugs or equal, Vinblastine (IC_50_ = 3.2 ± 1.2 μM) and Doxorubicin (IC_50_ = 1.1 ± 0.2 μM), while compounds **4a**, **4d**, **4k**, **4b**, **4n**, **4h** and **4o** with IC_50_ ranging from 1.4 to 3.1 μM showed anti-proliferative activity that was better than Vinblastine (IC_50_ = 3.2 ± 1.2 μM). Several compounds showed anti-proliferative activity that was less than Vinblastine (IC_50_ = 5.7 ± 1.1 μM), such as **4c**, **4d**, **4g**, **4e**, **4a**, **4i**, **4p**, **4f**, **4h**, **4n** and **4l** with IC_50_ values ranging from 1.7–5.3 μM against HeLa cells. Additionally, compounds **4a**, **4c**, **4d**, **4e**, **4g**, **4i**, **4k** and **4o** were weakly active against two normal cell lines, HFL-1 and WI-38, with an IC_50_ ranging from 20.4 to 39.1 μM. Furthermore, compounds **4i**, **4e**, **4d**, **4a**, **4g** and **4c** possessed good potency against MCF-7/ADR cells with IC_50_ = 8.6 ± 0.7, 10.0 ± 0.3, 10.4 ± 0.3, 10.8 ± 0.4, 11.5 ± 0.4 and 12.4 μM, respectively, as compared with Doxorubicin (IC_50_ = 18.6 ± 0.3 μM), while compounds **4o** and **4k** were inactive against MCF-7/ADR cells with IC_50_ = 23.7 ± 0.4 and 36.8 ± 0.32. Finally, the other compounds had moderate-to-fair cytotoxic activities. In the near future, we will assess this in an in vivo model using MDA-MB-231 xenografts grown on the chorioallantoic membrane of fertilized chick eggs (CAM). Additionally, we will then study the drug likeness for the benzochromene derivatives.

#### 2.4.2. *P*-Glycoprotein Expression and Function in MCF-7/ADR Cell Lysate

**Figure 5:**
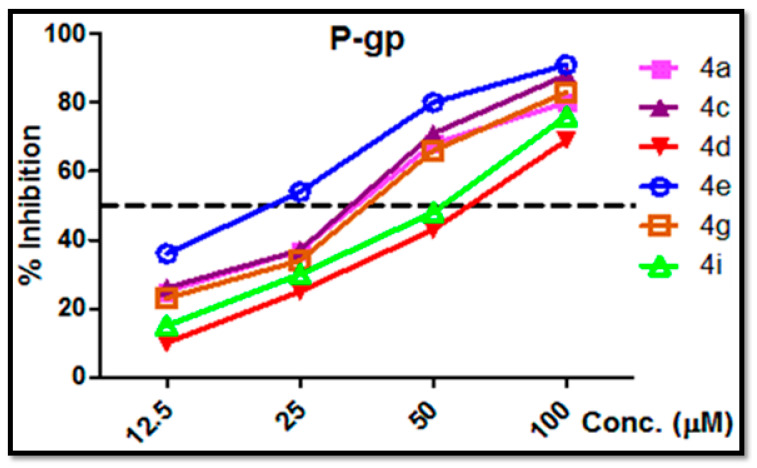
Inhibition of *P*-gp content in the lysate of MCF-7/ADR cells uses varying conc. (12.5–100 µM) of tested compounds **4a**, **4c**, **4d**, **4e**, **4g** and **4i** following an exposure of 48 h as determined by ELISA.

*P*-glycoprotein (*P*-gp)-mediated multidrug resistance (MDR) is a phenomenon in which cells become resistant to structurally and mechanistically unrelated drugs, resulting in low intracellular drug concentrations. It is one of the noteworthy problems in malignant tumor clinical therapeutics [53]. The most active compounds **4a**, **4c–4e**, **4g** and **4i** against MCF-7/ADR cells were tested as *P*-gp inhibitors using commercial ELISA kits (Human permeability glycoprotein ELISA Kit). The inhibition of *P*-gp content in MCF-7/ADR cell lysate using different compounds conc. as shown in Figure 5 and Table 2.

Table 2 indicated that compounds **4a**, **4c**, **4e** and **4g** that have 2,4-F_2_, 2,3-Cl_2_, 2,5-Cl_2_ and 3,4-Cl_2_ substituents have good potency against *P*-gp in MCF-7/ADR with IC_50_ ranging from 18.7 to 34.6 μM as compared with Doxorubicin (IC_50_ = 50.9 μM), as shown in Figure 5 and Table 2.

Despite having a high cytotoxic effect on MCF-7/ADR cells, chemicals **4d** and **4i** had an almost negligible inhibitory effect on the *P*-gp level (Figure 5); however, only **4a**, **4c** and **4e** had the strong inhibitory effect needed to be highly effective in reversing the MDR in MCF-7/ADR in earlier studies [54,55]. Furthermore, the reversion of *P*-gp-mediated multidrug resistance could be achieved by the down-regulation of *P*-gp expression and/or *P*-gp efflux function inhibition [56]. Hence, the effect of the current synthesized drugs was tested for inhibitory potential of *P*-glycoprotein activity using a Rhodamine 123 Accumulation Assay (Rhodamine Competitive ELISA Kit).

The information presented in Table 3 shows that Rhodamine 123 has a (IC_50_) range of 14.6–28.7 µM for evaluating the *P*-gp functional inhibition of **4a**, **4c** and **4e** in comparison with 14.3 µM referenced medication “Verapamil”. The compounds **4a**, **4c** and **4e** reduced *P*-gp expression and its function as well, which had an impact on the recovery of sensitivity to MCF-7/ADR cells. Contrarily, the cytotoxic effect of **4d**, **4g** and **4i** against MDR could be attributed to genetics, growth hormones and/or improved DNA repair capability [57].

#### 2.4.3. Cell Cycle Arrest in Treated MCF-7/ADR Cancer Cells 

**Figure 6:**
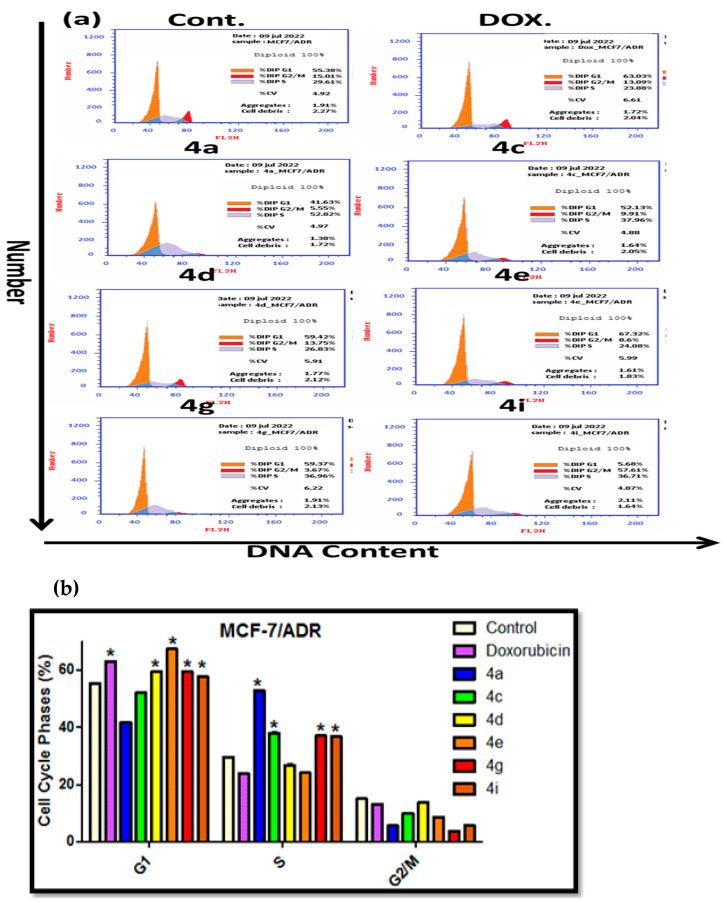
Effects of compounds **4a**, **4c–4e**, **4g** and **4i** on the cell cycle phases of MCF-7/ADR cells. (**a**) Representative histograms of the DNA content distribution of cells were incubated with IC_50_ values for 24 h and stained with propidium iodide (PI). Their DNA content was analyzed using fluorescence flow cytometry. (**b**) The percentage of MCF-7/ADR cells in the G1, S and G2/M phases after incubation with tested compounds **4a**, **4c–4e**, **4g** and **4i** (IC_50_ value) for 24 h. The data are expressed as the mean ± SD of three independent experiments in triplicate. Significances are shown in comparison to control cells (* *p* < 0.001).

Cancer cells undergo unscheduled cell divisions by the down-regulation of the four cell cycle stages (G1, S, G2 and M); therefore, the development of anticancer agents targeting cell cycle arrest represents an important therapeutic intervention [58,59]. *P*-gp breast cancer resistance proteins (BCRP) inhibit the action of anticancer drugs on cell cycle arrest by preventing intercellular drug accumulation [60]. By using flow cytometry and the FACS Calibers, it was possible to examine how the potent-produced compounds (**4a**, **4c**, **4e**, **4g** and **4i**) affected the regulation of cell cycle progression in MCF-7/ADR cells (Becton Dickinson). The distribution of cells along the G1 (2n), G2/M (4n) and S (2n to 4n) phases of the cycle was exhibited in the representative cell cycle distribution histogram of the stained DNA (Figure 6a). The MCF-7/ADR cancer cells were remedied with each derivative at its IC_50_ values for 24 h, a controlled experiment with no treatment given with ” blank trials”. The outcomes of the cell cycle progression give three different results showing compounds with cell cyclic arrest at the G1 phase (**4d** and **4e**), while there were other compounds at the S phase (**4a** and **4c**) and a final compound with cell cycle arrest at both of the G1/S phases (**4g** and **4i**). Additionally, in comparison to the untreated controlled cells, these results were followed by a significant drop in percentage at the G2/M stages (Figure 6b). According to the cell cycle analysis, the tested derivatives considerably stopped the MCF-7/ADR cancer cells in the G1/S phase.

#### 2.4.4. Apoptosis Induction in MCF-7/ADR Cancer Cells

**Figure 7:**
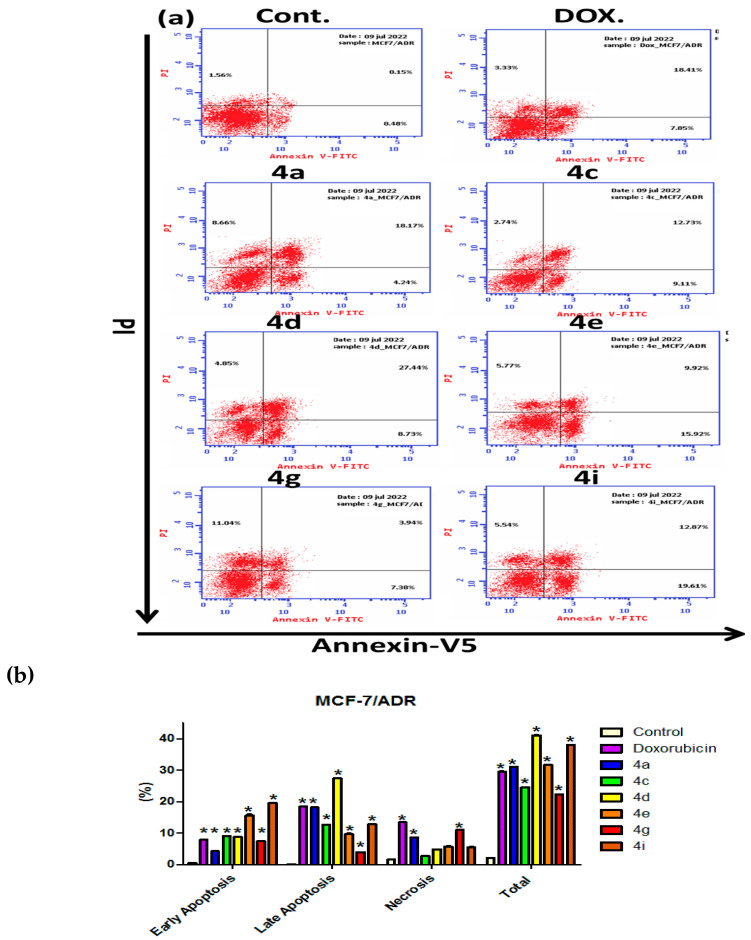
The apoptosis percentage of MCF-7/ADR cells after incubation with tested compounds **4a**, **4c–4e**, **4g** and **4i** (IC_50_ value) for 24 h. (**a**) The dot plot of the Annexin V/PI-stained cells, treated with the indicated drugs. (**b**) The apoptosis percentage of MCF-7/ADR cells after incubation with tested compounds (IC50 value) for 24 h. The data are expressed as the mean ± SD of three independent experiments in triplicate. Significances are shown in comparison to control cells (* *p* < 0.001).

One of the optional mechanisms regarding the lethal action of chemotherapeutic medicines is blocking cell cycle progression, triggering apoptosis with otherwise mutual effect [61]. Moreover, *P*-gp prevents the release of cytochrome c, which is controlled by the intrinsic mitochondrial pathway, hence inhibiting apoptosis [62]. The pivotal relation of the newly synthesized MCF-7/ADR “anticancer drugs” was further evaluated using the Annexin V/PI double staining flow cytometric test [63]. Figure 7a shows specimen dot plots of the double-stained MCF-7/ADR cells following treatment with the various substances under investigation. In comparison to Doxorubicin’s 30% total apoptosis rate, which was nearly identical to **4a**, **4c** and **4g**, compounds **4d**, **4e** and **4i** were leading with percentages of 41%, 31% and 38%, respectively. Only compounds 4a (8.5%) and 4g (11%) had greater effects on necrosis compared to doxorubicin (5%). Other examined substances caused both early and late apoptosis in all treated cells (Figure 7b). Therefore, MCF-7/ADR cytotoxicity is induced by apoptotic processes, apparently without negative effects on *P*-gp.

#### 2.4.5. Structure Activity Relationship (SAR)

In a comparison of the cytotoxic activities of the two series, the disubstituents **4a–l** and the polysubstituents **4m–p** on the aryl groups linked to the 9-hydroxy-1*H*-benzo[*f*]-chromene scaffold at 1-postion against PC-3 cells; the activities of the first series were decreased in the order of 3,5-Br_2_ > 2,4-F_2_ > 2,4-Cl_2_ > 2,5-Cl_2_ > 3,4-Cl_2_ > 2,3-Cl_2_ > 2,4-(MeO)_2_ > 3,4-(MeO)_2_ > 2,6-F_2_ > 2,6-ClF > 2,6-Cl_2_ > 2,3-HO,MeO with IC_50_ ranging from 0.8 to 33.5 μM, and for the second series in the order of 3,4,5-(MeO)_3_ > C_6_F_5_ > 2,3,4-(MeO)_3_ > 2,4,5-(MeO)_3_ with IC_50_ ranging from 2.1 to 7.6 μM, implying that grafting a lipophilic bulky electron-withdrawing substituent such as halogens is more beneficial than a non-bulky electron-donating substituent such as methoxy for the activity. Furthermore, exploration of the impact of the substitution on the 1-position of the pendant phenyl group showed that the order of anti-proliferative activities of the disubstituents **4a–l** against SKOV-3 cells were decreased in the order of 3,4-Cl_2_ > 2,5-Cl_2_ > 3,5-Br_2_ > 2,4-F_2_ > 2,4-Cl_2_ > 2,4-(MeO)_2_ > 2,6-F_2_ > 2,6-ClF > 2,3-Cl_2_ > 3,4-(MeO)_2_ > 2,6-Cl_2_ > 2,3-HO,MeO with IC_50_ ranging from 0.9 to 97.4 μM, and for the second series in the order of 2,4,5-(MeO)_3_ > 3,4,5-(MeO)_3_ > 2,3,4-(MeO)_3_ > C_6_F_5_ with IC_50_ ranging from 2.4 to 4.2 μM, hinting that grafting a lipophilic bulky electron-withdrawing substituent such as halogens has greatly enhanced the activity. Concerning the effect of the two series, the disubstituents **4a–l** and the polysubstituents **4m–p** against HeLa cells, the activities were decreased in the order of 2,3-Cl_2_ > 2,4-Cl_2_ > 3,4-Cl_2_ > 2,5-Cl_2_ > 2,4-F_2_ > 3,5-Br_2_ > 2,6-Cl_2_ > 2,6-ClF > 3,4-(MeO)_2_ > 2,4-(MeO)_2_ > 2,6-F_2_ > 2,3-HO,MeO with IC_50_ ranging from 1.7 to 50.4 μM, and for the second series the order of anti-proliferative activities was C_6_F_5_ > 2,4,5-(MeO)_3_ > 3,4,5-(MeO)_3_ > 2,3,4-(MeO)_3_ with IC_50_ ranging from 3.9 to 8.8 μM; this suggested that the prepared disubstituents **4a–l** exert their biological activity more than the polysubstituents **4m–p**. In addition, compounds **4i**, **4o**, **4d**, **4a**, **4g** and **4c** possessed good potency against MCF-7/ADR cells with IC_50_ ranging from 8.6–11.5 μM, as compared with Doxorubicin (IC_50_ = 18.6 μM), while compounds **4o** and **4k** were inactive against MCF-7/ADR cells with IC_50_ = 23.7 ± 0.4 and 36.8 ± 0.32 μM. Finally, compounds **4a**, **4c**, **4d**, **4e**, **4g**, **4i**, **4k** and **4o** had weak inhibitory activity against two normal cell lines, HFL-1 and WI-38, with IC_50_ ranging from 19.1 to 30.1 μM. In particular, compounds **4a**, **4c**, **4d**, **4e**, **4g**, **4i**, **4k** and **4o** were weakly active against these control normal cell lines, with strong selectivity in anti-proliferative effects for the cancer lines.

#### 2.4.6. Molecular Docking

In order to confirm the link between the in vitro cytotoxicity results and the binding affinity of hybrids **4a–p**, docking simulation experiments were carried out. More attention is devoted to *P*-glycoprotein (*P*-gp) which exists in cryo-EM structure; it contains the active site (PDB ID: 6FN1 [64]). The active site for 6FN1 was determined using CASTp to be Trp231, 1GLU874, MET875, GLN945 and MET948. The 3D loop structure of 6FN1 was created by the mGenTHERADER [65] within the docking framework, while the TS protein was created using the PyMol v:1.4 package. Nevertheless, the *P*-gp protein was validated using PROCHECK server [66], which also provided a 97.36 overall quality factor score on ERRAT server, to overview the statistics of nonbonding interactions between various atom types. When compared to the reference drug Zosuquidar, which blocked Glu874 and Trp231 with an RMSD of 1.20 Å, the toxicity behavior of 4a-p molecules was demonstrated in binding energy (BE) with a P-gp receptor. After being re-docked, the studied 4a-p hybrids attained a root mean square deviation (RMSD) of less than 2 Å (Table 4). To evaluate the binding affinity of the synthesized compounds, the pose with the lowest BE and RMSD was chosen. To further support the validity of the BE, the inhibitory constant (Ki) and ligand efficacy (LE) were calculated [67].

**Figure 8:**
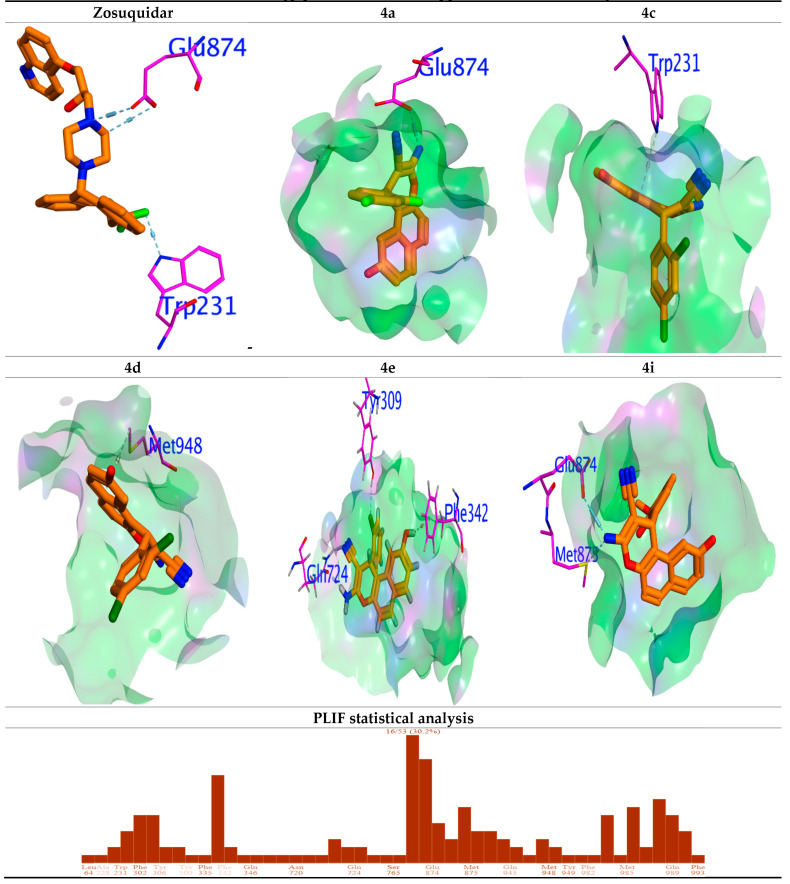
Binding interaction of most active compounds **4a**, **4c, 4d**, **4e** and **4i** into *P*-gp, and PLIF statistical analysis histogram for compounds **4a–p** and residues of *P*-gp protein.

The most active compounds (**4a**, **4c**, **4d**, **4e** and **4i**) display promising binding scores (−6.30, −7.17, −7.10, −8.12 and −6.07 Kcal/mol), respectively, compared to Zosuquidar (−8.22 Kcal/mol). These BE values are supportive to the cytotoxicity data for tested compounds. All compounds were successful in blocking active binding sites in a manner similar to Zosuquidar. The docking statistical analysis implemented herein favors 30% of the tested compounds and can inhibit the subpocket *P-gp* protein by blocking the vital amino acid Glu 874 in the binding pocket (Figure 8). 

The most active compound **4e** bearing 2,5-Cl_2_ substituents (IC_50_ 18.7 μM) showed the most binding affinity −8.12 Kcal/mol and inhibited the binding site by the formation of two strong H-bonding interactions with Tyr309, Gln7724 and Phe342 (Figure 8). The compound showed promising bioactive parameters LE, Ki and FQ (Table 4) but moderate activity was obtained for compounds **4a** and **4c** bearing 2,4-F_2_ and 2,3-Cl_2_ substituents.

The 2,4-F_2_ and 2,3-Cl_2_ rings are arranged with the main GLU874 and TRP231 in a perpendicular manner to be stabilized in the binding pocket, in addition to strong H-bonding interactions with the respective amino acids. For all compounds, the bioactivity metrics LE, Ki and FQ were found within the traditional range [68]. As per the docking outcomes, *P*-gp protein inhibition supports the in vitro findings.

## 3. Experimental Section

### 3.1. Materials and Equipment

All chemicals were purchased from Sigma-Aldrich Chemical Co. (Sigma-Aldrich Corp., St. Louis, MO, USA). All the melting points were measured with a Stuart Scientific Co. Ltd. Apparatus (Camberley, UK), which means they are uncorrected. The IR spectra were recorded on a KBr disc on a Jasco FT/IR 460 plus spectrophotometer (Jasco, Tokyo, Japan). The ^1^H/^13^C (500/125 MHz) NMR spectra were measured on a BRUKER AV 500 MHz spectrometer (BRUKER, Billerica, MA, USA) in DMSO-d_6_, as solvent, using tetramethylsilane (TMS) as an internal standard. The microwave apparatus utilized is Milestone Sr1, Microsynth (Milstone, Shelton, CT, USA.). The mass spectra were determined on a Shimadzu GC/MS-QP5050A spectrometer (Shimadzu, Kyoto, Japan). The elemental analysis was carried out at the Regional Center for Mycology and Biotechnology (RCMP), Al-Azhar University, Cairo, Egypt, and the results were within ± 0.25%. The reaction courses and products were routinely monitored using thin layer chromatography (TLC) on silica gel precoated F_254_ Merck plates (Merck, Billerica, MA, USA).

### 3.2. General Procedure for Synthesis of 1H-Benzo[f]chromene Derivatives (***4a–p***)

A reaction mixture of naphthalene-2,7-diol (**1**) (0.01 mol), malononitrile (**2**) (0.01 mol), different aromatic aldehydes (**3a–p**) and piperidine (0.5 mL) in an absolute ethanol solution (30 mL) was heated under microwave irradiation conditions for 2 min at 140 °C. After the reaction reached completion, the reaction mixture was cooled to room temperature and the precipitated solid was filtered off, washed with methanol and recrystallized from ethanol/benzene. The physical and spectral data of compounds **(4a–p)** are as follows: 

#### 3.2.1. 3-Amino-1-(2,4-Difluorophenyl)-9-Hydroxy-1*H*-Benzo[*f*]chromene-2-Carbonitrile (**4a**)

Orange crystals; yield 88%; m.p. 203–204 °C; IR (KBr) *υ* (cm^−1^): 3423, 3414, 3328, 3208 (NH_2_ and OH), 2193 (CN); ^1^H NMR *δ*: 9.96 (s, 1H, OH), 8.04 (t, 1H, *J* = 8.8 Hz, H-7), 8.00 (s, 1H, H-10), 7.80 (d, 1H, *J* = 8.8 Hz, H-6), 7.76 (d, 1H, *J* = 8.7 Hz, H-5), 7.24 (m, 1H, *J* = 5.5 Hz, H-8), 7.04 (bs, 2H, NH_2_), 7.06 (1H, d, *J* = 2.21 Hz, Ar, H-6), 6.98 (s, 1H, Ar, H-3), 6.89 (1H, d, *J* = 2.21 Hz, Ar, H-5), 5.29 (s, 1H, H-1); ^13^C NMR *δ*: 160.6 (C-3), 157.0 (C-9), 152.3(C-4a), 150.1, 148.1 (C-2,4, Ar, d, *J* = 250 Hz, C-F), 132.3 (C-10a), 130.8 (C-6, Ar), 130.1 (C-7), 130.0 (C-6), 125.6 (C-6a, C-1, Ar), 120.7 (C-10b), 117.7 (CN), 116.4 (C-5, Ar), 115.7 (C-8), 113.6 (C-5), 110.5 (C-3, Ar), 105.3 (C-10), 56.3 (C-2), 32.3 (C-1); MS *m*/*z* (%): 350 (M^+^, 100); Anal. Calcd for C_20_H_12_F_2_N_2_O_2_ (350.32): C, 68.57; H, 3.45; N, 8.00. Found: C, 68.64; H, 3.51; N, 8.06%. 

#### 3.2.2. 3-Amino-1-(2,6-Difluorophenyl)-9-Hydroxy-1*H*-Benzo[*f*]chromene-2-Carbonitrile (**4b**)

Colorless crystals; yield 85%; m.p. 193–194 °C; IR (KBr) *υ* (cm^−1^): 3457, 3445, 3322, 3226 (NH_2_ and OH), 2175 (CN); ^1^H NMR *δ*: 10.00 (s, 1H, OH), 7.78 (d, 1H, *J* = 8.9 Hz, H-7), 7.75 (d, 1H, *J* = 8.8 Hz, H-6), 7.32 (m, 1H, *J* = 4.9 Hz, H-5), 7.09 (bs, 2H, NH_2_), 7.06 (t, 1H, *J* = 9.0 Hz, Ar, H-4), 7.03 (d, 1H, *J* = 8.9 Hz, H-10), 6.99 (q, 2H, *J* = 3.7 Hz, Ar, H-3,5), 6.90 (d, 1H, *J* = 2.1 Hz, H-8), 5.45 (s, 1H, H-1); ^13^C NMR *δ*: 161.0 (C-3), 157.1 (C-9, C-4a), 150.1, 148.2 (C-2,6, Ar, d, *J* = 240 Hz, C-F), 132.5 (C-10a), 130.9 (C-7), 129.9 (C-6), 125.5 (C-6a), 120.7 (C-4, Ar), 120.3 (C-10b, C-1, Ar), 117.6 (CN), 113.5 (C-8), 112.6 (C-5), 111.4 (C-3,5, Ar), 104.5 (C-10), 54.1 (C-2), 28.6 (C-1); MS *m*/*z* (%): 350 (M^+^, 100); Anal. Calcd for C_20_H_12_F_2_N_2_O_2_ (350.32): C, 68.57; H, 3.45; N, 8.00. Found: C, 68.51; H, 3.40; N, 7.64%. 

#### 3.2.3. 3-Amino-1-(2,3-Dichlorophenyl)-9-Hydroxy-1*H*-Benzo[*f*]chromene-2-Carbonitrile (**4c**) 

Yellow crystals; yield 89%; m.p. 296–297 °C; IR (KBr) *υ* (cm^−1^): 3438, 3421, 3320, 3208 (NH_2_ and OH), 2181 (CN); ^1^H NMR *δ*: 10.01 (s, 1H, OH), 7.83–6.96 (m, 10H, aromatic and NH_2_), 5.65 (s, 1H, H-1); ^13^C NMR *δ*: 161.0 (C-3), 157.1 (C-9), 148.2 (C-4a), 146.1 (C-1, Ar), 132.5 (C-10a), 130.9 (C-3, Ar), 130.3 (C-7), 129.9 (C-2, Ar), 129.4 (C-5, Ar), 129.4 (C-6), 125.7 (C-4, Ar), 124.6 (C-6, Ar), 121.4 (C-6a), 120.3 (C-10b), 117.8 (CN), 113.6 (C-8), 111.1 (C-5), 105.4 (C-10), 56.5 (C-2), 35.64 (C-1); MS *m*/*z* (%): 386 (M^+^ + 4, 10.10), 384 (M^+^+2, 64.97), 382 (M^+^, 100); Anal. Calcd for C_20_H_12_Cl_2_N_2_O_2_ (383.23): C, 62.68; H, 3.16; N, 7.31. Found: C, 62.73; H, 3.20; N, 7.36%. 

#### 3.2.4. 3-Amino-1-(2,4-Dichlorophenyl)-9-Hydroxy-1*H*-Benzo[*f*]chromene-2-Carbonitrile (**4d**)

Colorless crystals; yield 88%; m.p. 221–222 °C; IR (KBr) *υ* (cm^−1^): 3463, 3441, 3339, 3197 (NH_2_ and OH), 2191 (CN); ^1^H NMR *δ*: 10.01 (s, 1H, OH), 7.82 (d, 1H, *J* = 8.8 Hz, H-7), 7.76 (d, 1H, *J* = 8.8 Hz, H-6), 7.66 (s, 1H, 10), 7.28 (d, 1H, *J* = 8.9 Hz, H-5), 7.08 (d, 1H, *J* = 8.9 Hz, H-8), 7.07 (bs, 2H, NH_2_), 7.01, 7.00 (dd, 2H, *J* = 8.7, 2.0 Hz, Ar H-5,6), 6.76 (s, 1H, Ar H-3), 5.50 (s, 1H, H-1); ^13^C NMR *δ*: 160.4 (C-3), 157.1 (C-9), 148.1 (C-4a), 142.1 (C-1, Ar), 132.7 (C-10a), 132.4 (C-2, Ar), 131.6 (C-4, Ar), 130.9 (C-6,3, Ar), 130.3 (C-7), 129.4 (C-6), 128.9 (C-5, Ar), 125.7 (C-6a), 120.3 (C-10b), 117.8 (CN), 113.6 (C-8), 112.9 (C-5), 105.5 (C-10), 56.1 (C-2), 31.2 (C-1); MS *m*/*z* (%): 386 (M^+^ + 4, 9.78), 384 (M^+^ + 2, 62.89), 382 (M^+^, 100); Anal. Calcd for C_20_H_12_Cl_2_N_2_O_2_ (383.23): C, 62.68; H, 3.16; N, 7.31. Found: C, 62.71; H, 3.19; N, 7.34%. 

#### 3.2.5. 3-Amino-1-(2,5-Dichlorophenyl)-9-Hydroxy-1*H*-Benzo[*f*]chromene-2-Carbonitrile (**4e**) 

Pale yellow crystals; yield 84%; m.p. 293–294 °C; IR (KBr) *υ* (cm^−1^): 3461, 3423, 3342, 3206 (NH_2_ and OH), 2187 (CN); ^1^H NMR *δ*: 10.01 (s, 1H, OH), 7.84–6.77 (m, 10H, aromatic and NH_2_), 5.54 (s, 1H, H-1); ^13^C NMR *δ*: 160.5 (C-3), 157.2 (C-9), 148.2 (C-4a), 146.2 (C-1, Ar), 132.9 (C-10a), 132.4 (C-5, Ar), 132.0 (C-3, Ar), 130.9 (C-7), 130.7 (C-6, Ar), 130.4 (C-2, Ar), 129.1 (C-6), 125.7 (C-4, Ar), 122.2 (C-6a), 120.2 (C-10b), 117.8 (CN), 113.7 (C-8), 111.1 (C-5), 105.4 (C-10), 56.5 (C-2), 35.0 (C-1); MS *m*/*z* (%): 386 (M^+^ + 4, 9.98), 384 (M^+^ + 2, 65.9), 382 (M^+^, 100); Anal. Calcd for C_20_H_12_Cl_2_N_2_O_2_ (383.23): C, 62.68; H, 3.16; N, 7.31. Found: C, 62.62; H, 3.11; N, 7.26%.

#### 3.2.6. 3-Amino-1-(2,6-Dichlorophenyl)-9-Hydroxy-1*H*-Benzo[*f*]chromene-2-Carbonitrile (**4f**)

Colorless crystals; yield 84%; m.p. 196–197 °C; IR (KBr) *υ* (cm^−1^): 3449, 3430, 3327, 3203 (NH_2_ and OH), 2190 (CN); ^1^H NMR *δ*: 9.88 (s, 1H, OH), 7.78 (d, 1H, *J* = 8.8 Hz, H-7), 7.74 (d, 1H, *J* = 8.7 Hz, H-6), 7.63 (dd, 1H, *J* = 7.9, 1.5 Hz, Ar H-3), 7.30 (t, 1H, *J* = 7.0 Hz, Ar H-4), 7.28 (dd, 1H, *J* = 7.0 Hz, Ar H-5), 7.05 (bs, 2H, NH_2_), 6.99 (d, 1H, *J* = 8.7, 2.2 Hz, H-8), 6.97 (s, 1H, 10), 6.72 (d, 1H, *J* = 2.5 Hz, H-5), 5.97 (s, 1H, H-1); ^13^C NMR *δ*: 160.9 (C-3), 156.9 (C-9), 148.8 (C-4a), 137.8 (C-1, Ar), 135.3, 135.20 (C-2,6, Ar), 132.8 (C-7), 131.3 (C-10a), 130.80 (C-4, Ar), 130.29 (C-6), 129.43 (C-3,5, Ar), 125.55 (C-6a), 120.12 (C-10b), 117.4 (CN), 113.4 (C-8), 111.1 (C-5), 105.7 (C-10), 53.2 (C-2), 35.6 (C-1); MS *m*/*z* (%): 386 (M^+^, 10.02) 384 (M^+^, 64.99) 382 (M^+^, 100); Anal. Calcd for C_20_H_12_Cl_2_N_2_O_2_ (383.23): C, 62.68; H, 3.16; N, 7.31. Found: C, 62.74; H, 3.22; N, 7.37%.

#### 3.2.7. 3-Amino-1-(3,4-Dichlorophenyl)-9-Hydroxy-1*H*-Benzo[*f*]chromene-2-Carbonitrile (**4g**)

Colorless crystals; yield 83%; m.p. 275–276 °C; IR (KBr) *υ* (cm^−1^): 3477, 3408, 3335, 3267 (NH_2_ and OH), 2179 (CN); ^1^H NMR *δ*: 9.94 (s, 1H, OH), 7.81 (d, 1H, *J* = 8.9 Hz, H-7), 7.76 (d, 1H, *J* = 8.7 Hz, H-6), 7.54 (d, 1H, *J* = 8.4 Hz, H-5), 7.43 (d, 1H, *J* = 2.1 Hz, Ar, H-5), 7.09 (d, 1H, *J* = 8.8 Hz, H-10), 7.08 (bs, 2H, NH_2_) 7.06 (s, 1H, Ar, H-2), 6.99 (dd, 1H, *J* = 8.7, 2.3 Hz, Ar, H-6), 6.95 (d, 1H, *J* = 2.3 Hz, H-8), 5.17 (s, 1H, H-1); ^13^C NMR *δ*: 160.2 (C-3), 157.0 (C-9), 147.8 (C-4a), 147.1 (C-1, Ar), 132.4 (C-10a), 131.6 (C-3, Ar), 130.8 (C-4, Ar), 130.2 (C-7, C-5, Ar), 129.7 (C-2, Ar), 129.2 (C-6), 127.8 (C-6, Ar), 125.7 (C-6a), 120.7 (C-10b), 117.7 (CN), 113. (C-8), 112.9 (C-5), 106.1 (C-10), 57.4 (C-2), 37.8 (C-1); MS *m*/*z* (%): 386 (M^+^ + 4, 10.01), 384 (M^+^ + 2, 46.07), 382 (M^+^, 70) with base peak at 237; Anal. Calcd for C_20_H_12_Cl_2_N_2_O_2_ (383.23): C, 62.68; H, 3.16; N, 7.31. Found: C, 62.61; H, 3.11; N, 7.27%.

#### 3.2.8. 3-Amino-1-(2-Chloro-6-Flourophenyl)-9-Hydroxy-1*H*-Benzo[*f*]chromene-2-Carbonitrile (**4h**)

Pale yellow crystals; yield 87%; m.p. 298–299 °C; IR (KBr) *υ* (cm^−1^): 3490, 3433, 3336, 3211 (NH_2_ and OH), 2187 (CN); ^1^H NMR *δ*: 9.93 (s, 1H, OH), 7.78 (d, 1H, *J* = 8.8 Hz, H-7), 7.75 (d, 1H, *J* = 8.8 Hz, H-6), 7.43 (s, 1H, Ar, H-3,5), 7.31 (d, 1H, *J* = 3.8 Hz, H-5), 7.07 (s, 1H, H-10), 7.01 (bs, 2H, NH_2_), 6.87 (s, 1H, Ar, H-4), 5.65 (s, 1H, H-1); ^13^C NMR *δ*: 162.9, 161.7 (C-6, Ar, d, *J* = 250 Hz, C-F), 160.9 (C-9), 157.0 (C-4a, C-3), 148.2 (C-2, Ar), 133.1 (C-10a), 132.7 (C-1, Ar), 130.8 (C-7), 130.1 (C-4, Ar), 130.0 (C-6), 126.1 (C-3, Ar), 125.5 (C-6a), 120.4 (C-10b), 117.5 (CN), 113.5 (C-8, C-5, Ar), 112.0 (C-5), 105.3 (C-10), 53.9 (C-2), 33.0 (C-1); MS *m*/*z* (%): 368 (M^+^ + 2, 65.96) 366 (M^+^, 100); Anal. Calcd for C_20_H_12_ClFN_2_O_2_ (366.77): C, 65.49; H, 3.30; N, 7.64. Found: C, 65.41; H, 3.23; N, 7.58%.

#### 3.2.9. 3-Amino-1-(3,5-Dibromophenyl)-9-Hydroxy-1*H*-Benzo[*f*]chromene-2-Carbonitrile (**4i**)

Colorless crystals; yield 86%; m.p. 283–284 °C; IR (KBr) *υ* (cm^−1^): 3463, 3418, 3347, 3196 (NH_2_ and OH), 2191 (CN); ^1^H NMR *δ*: 9.93 (s, 1H, OH), 7.91 (s, 1H, Ar, H-4), 7.83 (d, 1H, *J* = 8.8 Hz, H-7), 7.78 (t, 1H, *J* = 1.7 Hz, H-6), 7.68 (d, 1H, *J* = 8.8 Hz, H-5), 7.30 (s, 2H, Ar, H-2,6), 7.11 (bs, 2H, NH_2_), 7.09 (d, 1H, *J* = 8.9 Hz, H-10), 6.96 (d, 1H, *J* = 2.2 Hz, H-8), 5.18 (s, 1H, H-1); ^13^C NMR *δ*: 160.3 (C-3), 157.1 (C-9), 150.6 (C-4a), 147.7 (C-1, Ar), 133.4 (C-10a), 132.3 (C-4, Ar), 130.8 (C-2,6, Ar), 130.3 (C-7), 129.3 (C-6), 125.7 (C-6a), 123.8, 123.3 (C-3,5, Ar), 120.6 (C-10b), 117.8 (CN), 113.7 (C-8), 112.6 (C-5), 106.0 (C-10), 57.3 (C-2), 37.9 (C-1); MS *m*/*z* (%): 474 (M^+^ + 4, 35.07), 472 (M^+^ + 2, 76.97), 470 (M^+^, 40) with base peak at 237 (100); Anal. Calcd for C_20_H_12_Br_2_N_2_O_2_ (472.13): C, 50.88; H, 2.56; N, 5.93. Found: C, 50.81; H, 2.50; N, 5.87%.

#### 3.2.10. 3-Amino-1-(2-Hydroxy-3-Methoxyphenyl)-9-Hydroxy-1*H*-Benzo[*f*]chromene-2-Carbonitrile (**4j**)

Pale yellow crystals; yield 83%; m.p. 269–270 °C; IR (KBr) *υ* (cm^−1^): 3445, 3421, 3353, 3203 (NH_2_ and OH), 2214 (CN); ^1^H NMR *δ*: 13.54 (s, 1H, HO-2, Ar), 10.77 (s, 1H, HO-9), 7.87–6.85 (m, 10H, aromatic and NH_2_), 5.60 (s, 1H, H-1), 3.86 (s, 3H, OMe); ^13^C NMR *δ*: 164.6 (C-3), 163.8 (C-3, Ar), 161.3 (C-2, Ar), 150.8 (C-9), 149.0 (C-4a), 147.6 (C-10a), 139.6 (C-7), 135.3 (C-6), 130.8 (C-6a), 124.9 (C-5, Ar), 121.4 (C-6, Ar), 121.0 (C-10b), 120.5 (C-1, Ar), 118.6 (CN), 118.4 (C-4, Ar), 115.2 (C-8), 111.8 (C-5), 97.7 (C-10), 56.2 (C-2), 56.2 (Me), 35.9 (C-1); MS *m*/*z* (%): 360 (M^+^, 15.97) with a base peak at 238 (100); Anal. Calcd for C_21_H_16_N_2_O_4_ (360.36): C, 69.99; H, 4.48; N, 7.77. Found: C, 69.92; H, 4.42; N, 7.72%.

#### 3.2.11. 3-Amino-1-(2,4-Dimethoxyphenyl)-9-Hydroxy-1*H*-Benzo[*f*]chromene-2-Carbonitrile (**4k**)

Colorless crystals; yield 90%; m.p. 242–243 °C; IR (KBr) *υ* (cm^−1^): 3453, 3418, 3220, 3196 (NH_2_ and OH), 2212 (CN); ^1^H NMR *δ*: 9.83 (s, 1H, OH), 7.74 (d, 1H, *j* = 8.9 Hz, H-7), 7.72 (d, 1H, *j* = 8.8 Hz, H-6), 7.04 (d, 1H, *j* = 8.9 Hz, H-5), 6.97 (dd, 1H, *J* = 8.7, 2.3 Hz, Hz, Ar H-6), 6.92 (d, 1H, *j* = 2.5 Hz, H-10), 6.79 (s, 2H, NH_2_), 6.64 (d, 1H, *J* = 8.1 Hz, Ar, H-5), 6.59 (d, 1H, *J* = 2.5 Hz, Ar, H-8), 6.35 (dd, 1H, *J* = 8.5, 2.4 Hz, Ar, H-3), 5.30 (s, 1H, H-1), 3.88 (s, 3H, OMe), 3.69 (s, 3H, OMe); ^13^C NMR *δ*: 160.6 (C-3), 159.6 (C-2 Ar), 157.2 (C-4 Ar), 156.7 (C-9), 148.1 (C-4a), 132.7 (C-10a), 130.5 (C-6, Ar), 129.3 (C-7), 129.2 (C-6), 126.4 (C-6a), 125.6 (C-10b), 121.1 (C-8), 117.5 (CN), 114.7 (C-5), 114.4 (C-1, Ar), 106.0 (C-5, Ar), 105.9 (C-10), 99.0 (C-3, Ar), 57.8 (Me), 56.4 (Me), 55.6 (C-2), 32.7 (C-1); MS *m*/*z* (%): 374 (M^+^ + 1, 73) with base peak at 238; Anal. Calcd for C_22_H_18_N_2_O_4_ (374.39): C, 70.58; H, 4.85; N, 7.48. Found: C, 70.51; H, 4.79; N, 7.42%.

#### 3.2.12. 3-Amino-1-(3,4-Dimethoxyphenyl)-9-Hydroxy-1*H*-Benzo[*f*]chromene-2-Carbonitrile (**4l**)

Colorless crystals; yield 89%; m.p. 268–269 °C; IR (KBr) *υ* (cm^−1^): 3468, 3435, 3227, 3199 (NH_2_ and OH), 2184 (CN); ^1^H NMR *δ*: 9.87 (s, 1H, OH), 7.78 (d, 1H, *J* = 8.9 Hz, H-7), 7.74 (d, 1H, *J* = 8.8 Hz, H-6), 7.07 (d, 1H, *J* = 8.8 Hz, H-5), 7.02 (d, 1H, *J* = 2.4 Hz, H-10), 6.98 (dd, 1H, *J* = 8.8, 2.3 Hz, Ar, H-5), 6.90 (s, 2H, NH_2_), 6.82 (d, 1H, *J* = 8.4 Hz, H-8), 6.81 (d, 1H, *J* = 2.0 Hz, Ar, H-2), 6.54 (dd, 1H, *J* = 8.3, 2.1 Hz, Ar, H-6), 4.96 (s, 1H, H-1), 3.69 (s, 3H, OMe), 3.67 (s, 3H, OMe); ^13^C NMR *δ*: 160.0 (C-3), 156.7 (C-2, Ar), 149.1 (C-9), 147.9 (C-3, Ar), 147.7 (C-4 Ar), 138.6 (C-4a), 132.7 (C-1, Ar), 130.6 (C-10a), 129.6 (C-7), 125.7 (C-6), 121.2 (C-6a), 119.3 (C-10b, C-6, Ar), 117.5 (CN), 114.3 (C-8), 113.6 (C-5), 112.5 (C-2, Ar), 111.3 (C-5, Ar), 106.3 (C-10), 58.6 (Me), 55.9 (C-2), 38.5 (C-1); MS *m*/*z* (%): 375 (M^+^ + 1, 100); Anal. Calcd for C_22_H_18_N_2_O_4_ (374.39): C, 70.58; H, 4.85; N, 7.48. Found: C, 70.64; H, 4.89; N, 7.55%.

#### 3.2.13. 3-Amino-1-(2,3,4-Trimethoxyphenyl)-9-Hydroxy-1*H*-Benzo[*f*]chromene-2-Carbonitrile (**4m**)

Pale yellow crystals; yield 90%; m.p. 265–266 °C; IR (KBr) *υ* (cm^−1^): 3438, 3414, 3222, 3197 (NH_2_ and OH), 2203 (CN); ^1^H NMR *δ*: 9.82 (s, 1H, OH), 7.74 (d, 1H, *j* = 8.9 Hz, H-7), 7.72 (d, 1H, *j* = 8.7 Hz, H-6), 7.05 (s, 1H, H-10), 7.04 (d, 1H, *j* = 6.5 Hz, H-5), 6.98 (d, 1H, *j* = 8.5 Hz, H-8), 6.88 (s, 2H, NH_2_), 6.67, 6.66 (dd, 2H, Ar H-5,6), 5.18 (s, 1H, H-1), 3.78 (s, 3H, OMe), 3.74 (s, 3H, OMe), 3.70 (s, 3H, OMe); ^13^C NMR *δ*: 160.6 (C-3), 156.7 (C-9), 152.6 (C-4a), 150.8 (C-4, Ar), 147.8 (C-2, Ar), 141.7 (C-3 Ar), 132.7 (C-10a), 131.2 (C-7), 130.5 (C-6), 129.3 (C-6, Ar), 125.6 (C-6a), 123.6 (C-10b), 121.5 (C-5, Ar), 117.4 (CN), 114.8 (C-8), 113.7 (C-5), 108.5 (C-1, Ar), 106.0 (C-10, Ar), 61.6 (Me), 60.7 (Me), 57.7 (Me), 56.5 (C-2), 33.6 (C-1); MS *m*/*z* (%): 405 (M^+^ + 1, 100); Anal. Calcd for C_23_H_20_N_2_O_5_ (404.14): C, 68.31; H, 4.98; N, 6.93. Found: C, 68.38; H, 5.05; N, 6.99%.

#### 3.2.14. 3-Amino-1-(2,4,5-Trimethoxyphenyl)-9-Hydroxy-1*H*-Benzo[*f*]chromene-2-Carbonitrile (**4n**)

Colorless crystals; yield 87%; m.p. 235–236 °C; IR (KBr) *υ* (cm^−1^): 3452, 3419, 3325, 3227, 3197 (NH_2_ and OH), 2192 (CN); ^1^H NMR *δ*: 9.82 (s, 1H, OH), 7.74 (d, 1H, *J* = 8.9 Hz, H-7), 7.72 (d, 1H, *J* = 8.6 Hz, H-6), 7.05 (d, 1H, *J* = 8.8 Hz, H-5), 6.98 (d, 1H, *J* = 6.0 Hz, H-8), 6.82 (s, 2H, NH_2_), 6.71 (s, 1H, H-10), 6.36 (s, 1H, Ar, H-3,6), 5.30 (s, 1H, H-1), 3.84 (s, 3H, OMe), 3.76 (s, 3H, OMe), 3.48 (s, 3H, OMe); ^13^C NMR *δ*: 160.6 (C-3), 156.7 (C-2, Ar), 150.9 (C-9), 149.1 (C-4, Ar), 148.0 (C-4a), 143.3 (C-5, Ar), 132.7 (C-10a), 130.5 (C-7), 129.3 (C-6), 125.6 (C-6a), 125.3 (C-6, Ar), 121.1 (C-10b), 117.5 (CN), 114.6 (C-8), 113.6 (C-5), 106.0 (C-10), 99.2 (C-3, Ar), 57.6 (Me), 57.3 (Me), 56.9 (Me), 56.2 (C-2), 31.2 (C-1); MS *m*/*z* (%): 405 (M^+^ + 1, 100); Anal. Calcd for C_23_H_20_N_2_O_5_ (404.14): C, 68.31; H, 4.98; N, 6.93. Found: C, 68.37; H, 5.05; N, 6.99%.

#### 3.2.15. 3-Amino-1-(3,4,5-Trimethoxyphenyl)-9-Hydroxy-1*H*-Benzo[*f*]chromene-2-Carbonitrile (**4o**)

Colorless crystals; yield 89%; m.p. 252–253 °C; IR (KBr) *υ* (cm^−1^): 3461, 3427, 3294, 3177 (NH_2_ and OH), 2204 (CN); ^1^H NMR *δ*: 9.90 (s, 1H, OH), 7.80 (d, 1H, *j* = 8.9 Hz, H-7), 7.77 (d, 1H, *j* = 8.7 Hz, H-6), 7.09 (d, 1H, *j* = 8.8 Hz, H-5), 7.05 (d, 1H, *j* = 8.5 Hz, H-8), 7.00 (s, 1H, H-10), 6.94 (dd, 2H, *J* = 8.7, 2.3 Hz, Ar, H-2,6,), 6.41 (s, 2H, NH_2_), 4.98 (s, 1H, H-1), 3.66 (s, 6H, 2OMe), 3.60 (s, 3H, OMe); ^13^C NMR *δ*: 160.2 (C-3), 156.8 (C-3,5 Ar), 153.4 (C-9), 147.7 (C-4a), 141.8 (C-4, Ar), 136.6 (C-1, Ar), 132.7 (C-10a), 130.6 (C-7), 129.8 (C-6), 125.7 (C-6a), 121.1 (C-10b), 117.6 (CN), 114.0 (C-8), 113.7 (C-5, Ar), 106.2 (C-2,6, Ar), 104.6 (C-10), 60.4 (Me), 58.3 (Me), 56.2 (C-2), 39.1 (C-1); MS *m*/*z* (%): 405 (M^+^ + 1, 100); Anal. Calcd for C_23_H_20_N_2_O_5_ (404.14): C, 68.31; H, 4.98; N, 6.93. Found: C, 68.26; H, 4.93; N, 6.89%.

#### 3.2.16. 3-Amino-9-Hydroxy-1-(Perfluorophenyl)-1*H*-Benzo[*f*]chromene-2-Carbonitrile (**4p**)

Yellow crystals; yield 79%; m.p. 299–300 °C; IR (KBr) *υ* (cm^−1^): 3485, 3421, 3353, 3203 (NH_2_ and OH), 2192 (CN); ^1^H NMR *δ*: 10.10 (s, 1H, OH), 7.82 (d, 1H, *J* = 8.9 Hz, H-7), 7.79 (d, 1H, *j* = 8.8, Hz, H-6), 7.27 (s, 2H, NH_2_), 7.05 (d, 1H, *j* = 8.8 Hz, H-5), 7.00 (q, 1H, *J* = 3.66 Hz, H-10), 6.82 (d, 1H, *j* = 2.2, H-8), 5.57 (s, 1H, H-1); ^13^C NMR *δ*: 161.1 (C-3), 157.4 (C-9), 157.3 (C-4a), 148.3 (C-3,5 Ar), 132.3 (C-4, Ar), 132.2 (C-2,6 Ar), 131.1 (C-10a), 130.6 (C-7), 130.3 (C-6), 125.5 (C-6a), 120.3 (C-10b), 117.9 (CN), 113.5 (C-8), 110.6 (C-5), 109.9 (C-1, Ar), 104.2 (C-10), 53.3 (C-2), 29.8 (C-1); MS *m*/*z* (%): 405 (M^+^, 100) with a base peak at 375 (100); Anal. Calcd for C_20_H_9_F_5_N_2_O_2_ (404.29): C, 59.42; H, 2.24; N, 6.93. Found: C, 59.49; H, 2.30; N, 6.99%.

### 3.3. Biological Screening

#### 3.3.1. Cell Culture

The tumor cell lines MCF-7, HepG-2, PC-3 MCF-7/ADR, HFL-1 and (WI-38) were obtained from the American Type Culture Collection (ATCC, Rockville, MD, USA). The cells were grown on RPMI-1640 medium supplemented with 10% inactivated fetal calf serum and 50 µg/mL gentamycin. The cells were maintained at 37 ^ο^C in a humidified atmosphere with 5% CO_2_ and were subcultured two to three times a week. 

#### 3.3.2. Cytotoxicity Evaluation using Viability Assay 

The cytotoxic activity was appraised using the 3-(4,5-dimethylthiazol-2-yl)-2,5-diphenyltetrazolium bromide (MTT) colorimetric assay for the antitumor cell lines, as reported previously [52], and the ELISA assay for MCF-7/ADR [69]. 

#### 3.3.3. In Vitro Analysis of *P*-gp Content

The content of *P*-gp in the MCF-7/ADR cell lysates after incubation with varying conc. (12.5–100 µM) of tested compounds **4a**, **4c**, **4d**, **4e**, **4g** and **4i** following exposure for 48 hr was determined using a commercial human *P*-gp (Permeability Glycoprotein) ELISA Kit (MBS2506188, MyBioSource Inc., San Diego, CA, USA). Absorption was recorded at 450 nm with a Spectramax Gemini fluorescence microplate reader (Molecular Devices, Sunnyvale, CA, USA) [70].

#### 3.3.4. Rhodamine 123 Accumulation Assay

*P*-gp activity was determined by measuring intracellular accumulation of Rhodamine 123 in MCF-7/ADR cells in the absence or presence of compounds **4a**, **4c**, **4d**, **4e**, **4g** and **4i**, according to the commercial Rhodamine Competitive ELISA Kit (AKR-5142, Cell Biolabs Inc., San Diego, CA, USA) which provides a convenient method for the detection of total rhodamine in extracts from cells [71]. Briefly, resistant cancer cells were harvested, washed twice and counted. After dilution to 1 × 10^6^ cells/mL in each well of 6 well plates, 1 mL of fresh media containing different concentrations of compounds **4a**, **4c**, **4d**, **4e**, **4g** and **4i** were added and incubated at 37°C for 4 h in an atmosphere containing 5% CO_2_. Subsequently, 5.25 μM of Rho123 was added to each well and the wells were incubated for another 30 min at 37 °C. Finally, cells were washed and lysed as described before, and intracellular quantification levels of Rhodamine 123 were further analyzed according to the ELISA protocol kit. Absorbance at 450 nm of each well was measured using a Spectramax Gemini fluorescence microplate reader (Molecular Devices, Sunnyvale, CA, USA). The total content of Rhodamine in each sample was determined by comparison with a Rhodamine standard curve.

### 3.4. Molecular Docking

We have used a program similar to that used by Jaguar et al. [72] to generate all possible tautomeric and stereo-isomeric stats for the structures. Crystal structures of *P*-glycoprotein were taken from the protein data bank and bonded with 5-florouracil as the reference drug. All ligands were imported into Ligprep module and redocked into the appropriate binding sites using Glide’s module. The Glide tool was applied to perform the molecular docking, then a grid for protein was charged using the default aspects of force field. The (SP) scoring function was produced to study the binding affinity, and then charged with a Charm force field. The low root square devotion RMSD score was utilized to obtain the other poses. To draw, the Schrodinger builder was applied.

## 4. Conclusions

In summary, we have synthesized di/polysubstituted 1*H*-benzo[*f*]chromene derivatives and evaluated their cytotoxic activities against three cancer cell lines (PC-3, SKOV-3 and HeLa). Several target compounds showed cytotoxic activity against PC-3, SKOV-3 and HeLa cancer cell lines. In particular, compounds **4a**, **4c**, **4d**, **4e**, **4g**, **4i**, **4k** and **4o** displayed the highest activity against PC-3, SKOV-3 and HeLa, with IC_50_ values ranging from 0.8–3.7, 0.9–3.1 and 1.7–5.7 μM, respectively. In addition, compounds **4i**, **4o**, **4d**, **4a**, **4g** and **4c**, respectively, possessed good potency against MCF-7/ADR cells with IC_50_ ranging from 8.6–11.5 μM and a weak inhibitory activity against two normal cell lines, HFL-1 and WI-38, with IC_50_ = 19.1–30.1 μM. The active compounds **4a**, **4c**, **4d**, **4e**, **4g** and **4i** against MCF-7/ADR lines were tested as *P*-gp expression and function inhibitors, and compounds **4a**, **4c**, **4e** and **4g** were found to have good inhibitory potency against *P*-gp levels in MCF-7/ADR lysate with IC_50_ ranging from 18.7 to 34.6 μM as compared with Doxorubicin (IC_50_ = 50.9 μM). Rho123 accumulation assays showed that compounds **4a**, **4c** and **4e** effectively inhibited *P*-pg expression and efflux function, while compounds **4d** and **4e** induced apoptosis and showed cell cyclic arrest at G1 phase, while other compounds showed this at S phase (**4a** and **4c**) and finally showed compounds with both cell cycle arrest at G1/S phases (**4g** and **4i**). 

The introduction of halogen atoms in 1-position on the pendant phenyl group plays a more important role in enhancing antitumor activities than methoxy groups, according to structure activity relationships (SARs).

## Figures and Tables

**Table 1:** IC_50_ values of the target compounds **4a–p** against PC-3, SKOV-3 and HeLa and the active compounds **4a**, **4c**, **4d**, **4e**, **4g**, **4i**, **4k** and **4o** against MCF-7/ADR, HFL-1 and WI-38 cell lines.

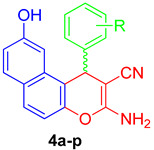							
Cancer Toxicity				IC_50_ µM ^a^			
				Normal Toxicity			
Cpd.	R	PC-3	SKOV-3	HeLa	MCF-7/ADR	HFL-1	WI-38
**4a**	2,4-F_2_	1.1 ± 0.4	1.4 ± 0.2	2.8 ± 0.3	10.8 ± 0.4	26.5 ± 0.3	34.3 ± 1.2
**4b**	2,6-F_2_	9.4 ± 0.5	2.0 ± 0.2	6.7 ± 0.2	-	-	-
**4c**	2,3-Cl_2_	2.9 ± 0.7	4.0 ± 0.6	1.7 ± 0.1	12.4 ± 0.1	22.1 ± 0.2	29.4 ± 0.1
**4d**	2,4-Cl_2_	2.0 ± 0.7	1.5 ± 0.1	1.8 ± 0.1	10.4 ± 0.3	20.1 ± 0.4	39.1 ± 0.2
**4e**	2,5-Cl_2_	2.1 ± 0.5	1.1 ± 0.4	2.7 ± 0.6	10.0 ± 0.3	24.1 ± 0.3	25.4 ± 1.1
**4f**	2,6-Cl_2_	26.6 ± 3.1	7.0 ± 0.4	4.1 ± 0.3	-	-	-
**4g**	3,4-Cl_2_	2.4 ± 0.1	0.9 ± 0.3	2.2 ± 0.2	11.5 ± 0.4	27.4 ± 0.1	26.3 ± 0.2
**4h**	2-Cl-6-F	11.6 ± 0.8	3.1 ± 0.2	4.7 ± 0.5	-	-	-
**4i**	3,5-Br_2_	0.8 ± 0.1	1.1 ± 0.1	3.4 ± 0.6	8.6 ± 0.7	38.1 ± 0.4	29.2 ± 1.2
**4j**	2-HO-3-MeO	33.5 ± 3.5	97.4 ± 0.8	50.4 ± 1.3	-	-	-
**4k**	2,4-(MeO)_2_	3.7 ± 0.3	1.7 ± 0.3	6.7 ± 0.1	36.8 ± 0.2	24.2 ± 0.1	25.3 ± 1.3
**4l**	3,4-(MeO)_2_	4.1 ± 0.9	4.0 ± 1.3	5.3 ± 0.2	-	-	-
**4m**	2,3,4-(MeO)_3_	6.5 ± 1.3	3.4 ± 0.5	8.8 ± 0.5	-	-	-
**4n**	2,4,5-(MeO)_3_	7.6 ± 1.2	2.4 ± 0.4	4.9 ± 0.5	-	-	-
**4o**	3,4,5-(MeO)_3_	2.1 ± 0.8	3.1 ± 0.1	5.7 ± 1.8	23.7 ± 0.4	22.2 ± 0.4	20.4 ± 0.3
**4p**	2,3,4,5,6-F_5_	4.5 ± 1.2	4.2 ± 0.3	3.9 ± 0.4	-	-	-
**A**	-	7.5 ± 1.3	3.2 ± 1.2	5.7 ± 1.1	-	-	-
**B**	-	1.3 ± 0.3	1.1 ± 0.2	1.5 ± 0.3	18.6 ± 0.2	-	-

^a^ IC_50_ values are expressed in (μM) as the mean values of triplicate wells from at least three experiments and are reported as the mean ± standard error, **A** = Vinblastine and **B** = Doxorubicin.

**Table 2:** IC_50_ values of the active compounds **4a**, **4c–4e**, **4g** and **4i** against *P*-gp content in MCF-7/ADR cells.

	% Inhibition of *P*-Glycoprotein				IC_50_ µM
**Conc.**	100	50	25	12.5	
**4a**	80	68	37	25	32.0
**4c**	88	71	37	26	30.8
**4d**	69	43	25	10	65.9
**4e**	91	80	54	36	18.7
**4g**	83	66	34	23	34.6
**4i**	76	48	30	15	53.6
**Doxorubicin**					50.9

**Table 3:** *P*-gp inhibitory potential (IC_50_ values) based on *P*-gp content in MCF-7/ADR cell lysate and Rhodamine 123 accumulation assay.

Cpd.	MCF-7/ADR IC_50_ (µM)	*P*-gp IC_50_ (µM)	Rho123 IC_50_ (µM)
**4a**	10.8 ± 0.4	32.0	23.3
**4c**	12.4 ± 0.1	30.8	28.7
**4d**	10.4 ± 0.3	65.9	46.6
**4e**	10.0 ± 0.3	18.7	14.6
**4g**	11.5 ± 0.4	34.6	36.7
**4i**	8.6 ± 0.7	53.6	55.3
**Doxorubicin**	18.6 ± 0.2	50.9	-
**Verapamil**	-	-	14.3

**Table 4:** The binding affinity (kcal/mol) of **4a–p** hybrids against *P-gp* receptor.

Compd.	BE	RMSD	E_place_	E_conf_	E_ele_	LE	Ki	LE	FQ
**4a**	−6.30	1.56	−13.58	−20.98	−8.30	−11.83	2.47	−2.86	−8.97
**4b**	−7.28	1.48	−23.55	−19.33	−8.32	−7.24	1.98	−2.91	−4.33
**4c**	−7.17	1.00	−35.75	−18.87	−8.61	−5.85	1.77	−3.25	−2.60
**4d**	−7.10	1.55	−36.41	−21.56	−8.64	−4.11	1.41	−2.87	−1.24
**4e**	−8.12	0.88	−42.75	−17.62	−8.17	−6.93	1.94	−3.36	−3.57
**4f**	−6.47	2.74	−54.14	−14.72	−8.16	−5.66	1.73	−2.37	−3.28
**4g**	−6.37	1.07	−49.18	−20.96	−8.57	−4.77	1.56	−3.19	−1.58
**4h**	−6.23	1.90	−50.19	−22.77	−9.35	−1.17	0.16	−2.69	1.52
**4i**	−6.07	1.12	−55.13	−29.86	−8.38	−6.14	1.81	−3.15	−2.98
**4j**	−6.45	0.95	−33.24	−21.72	−9.92	−4.19	1.43	−3.30	−0.89
**4k**	−6.81	0.60	−17.16	−18.71	−8.05	−4.42	1.49	−3.69	−0.73
**4l**	−6.78	1.20	−23.59	−22.38	−8.54	−2.56	0.94	−3.09	0.53
**4m**	−6.65	1.19	−45.68	−18.53	−10.14	−6.58	1.88	−3.10	−3.49
**4n**	−5.55	0.95	−23.07	−25.30	−8.74	−7.61	2.03	−3.30	−4.31
**4o**	−5.79	1.18	−56.73	−18.25	−8.24	−4.67	1.54	−3.10	−1.56
**4p**	−6.53	0.72	−44.91	−23.71	−8.93	−8.65	2.16	−3.53	−5.12
**Zosuquidar**	−8.22	1.25	252.14	−44.03	−11.75	−3.88	1.36	−3.06	−0.83

BE, E_conf_ and E_place_ are free binding energy for the ligand from a given pose, conformer and receptor; RMSD: Root Mean Square Deviation; E_ele_: Electrostatic interaction with the receptor; LE: Ligand efficiency. Ki—inhibition constant, FQ—Fit quality.

## Data Availability

Not applicable.

## Supplementary Materials

The following spectral data supporting information can be downloaded at https://www.mdpi.com/article/10.3390/ijms24010049/s1,
