# Supplementary material for: Synthesis of 9-Hydroxy-1H-Benzo[f]chromene Derivatives with Effective Cytotoxic Activity on MCF7/ADR, P-Glycoprotein Inhibitors, Cell Cycle Arrest and Apoptosis Effects
Source: Int J Mol Sci. 2022 Dec 20;24(1):49. doi: 10.3390/ijms24010049 (PMC9820082; doi:10.3390/ijms24010049)
Supplement: Supplementary file 1 [file ijms-24-00049-s001.zip › ijms-2016381-supplementary.pdf]

Supplementary Material

Synthesis of 9-Hydroxy-1*H*-Benzo[*f*]chromene Derivatives with Effective Cytotoxic Activity on MCF7/ADR, *P*-Glycoprotein Inhibitors, Cell Cycle Arrest and Apoptosis

Fawzia F. Albalawi 1,*, Mohammed A. A. El-Nassag 2, Raafat A. El-Eisawy 2,3, Mahmoud Basseem I. Mohamed 2 , Ahmed M. Fouda 4 , Tarek H. Afifi 1 , Ahmed A. Elhenawy 2,5 , Ahmed Mora 2 , Ahmed M. El-Agrody 2,* and Heba K. A. El-Mawgoud 6

1 Chemistry Department, Faculty of Science, Taibah University, Al-Madinah Al-Munawarah 30002, Saudi Arabia
2 Chemistry Department, Faculty of Science, Al-Azhar University, Cairo 11884, Egypt
3 Chemistry Department, Faculty of Science and Art, Al-Baha University, Al-Baha 65582, Saudi Arabia
4 Chemistry Department, Faculty of Science, King Khalid University, Abha 61413, Saudi Arabia
5 Chemistry Department, Faculty of Science and Art, Albaha University, Albahah 65731, Saudi Arabia
6 Chemistry Department, Faculty of Women for Arts, Science, and Education, Ain Shams University, Cairo 11757, Egypt
* Correspondence: ffs.chem443@gmail.com (F.F.A.); elagrody_am@azhar.edu.eg (A.M.E.-A.)

Table of Contents:	Page
**Figure S1:** ^{1}H NMR 8.5-6.5 ppm of cpd. **(4a)**.	S4
**Figure S2:** ^{1}H NMR of cpd. **(4a)**.	S5
**Figure S3:** ^{13}C NMR of cpd. **(4a)**.	S6
**Figure S4:** ^{1}H NMR 8.5-6.5 ppm of cpd. **(4b)**.	S7
**Figure S5:** ^{1}H NMR of cpd. **(4b)**.	S8
**Figure S6:** ^{13}C NMR of cpd. **(4b)**.	S9
**Figure S7:** ^{1}H NMR of cpd. **(4c)**.	S10
**Figure S8:** ^{13}C NMR ppm of cpd. **(4c)**.	S11
**Figure S9:** ^{1}H NMR 8.5-6.5 ppm of cpd. **(4d)**.	S12
**Figure S10:** ^{1}H NMR of cpd. **(4d)**.	S13
**Figure S11:** ^{13}C NMR of cpd. **(4d)**.	S14
**Figure S12:** ^{1}H NMR of cpd. **(4e)**.	S15
**Figure S13:** ^{13}C NMR ppm of cpd. **(4e)**.	S16
**Figure S14:** ^{1}H NMR 8.5-6.5 ppm of cpd. **(4f)**.	S17
**Figure S15:** ^{1}H NMR of cpd. **(4f)**.	S18
**Figure S16:** ^{13}C NMR of cpd. **(4f)**.	S19
**Figure S17:** ^{1}H NMR 8.5-6.5 ppm of cpd. **(4g)**.	S20
**Figure S18:** ^{1}H NMR of cpd. **(4g)**.	S21
**Figure S19:** ^{13}C NMR of cpd. **(4g)**.	S22
**Figure S20:** ^{1}H NMR 8.5-6.5 ppm of cpd. **(4h)**.	S23
**Figure S21:** ^{1}H NMR of cpd. **(4h)**.	S24
**Figure S22:** ^{13}C NMR of cpd. **(4h)**.	S25
**Figure S23:** ^{1}H NMR 8.5-6.5 ppm of cpd. **(4i)**.	S26
**Figure S24:** ^{1}H NMR of cpd. **(4i)**.	S27
**Figure S25:** ^{13}C NMR of cpd. **(4i)**.	S28
**Figure S26:** ^{1}H NMR of cpd. **(4j)**.	S29
**Figure S27:** ^{13}C NMR of cpd. **(4j)**.	S30
**Figure S28:** ^{1}H NMR 8.5-6.5 ppm of cpd. **(4k)**.	S31
**Figure S29:** ^{1}H NMR of cpd. **(4k)**.	S32
**Figure S30:** ^{13}C NMR of cpd. **(4k)**.	S33
**Figure S31:** ^{1}H NMR 8.5-6.5 ppm of cpd. **(4l)**.	S34
**Figure S32:** ^{1}H NMR of cpd. **(4l)**.	S35
**Figure S33:** ^{13}C NMR of cpd. **(4l)**.	S36

Figure S34: ^{1}H NMR 8.5-6.5 ppm of cpd. **(4m)**. S37
Figure S35: ^{1}H NMR of cpd. **(4m)**. S38
Figure S36: ^{13}C NMR of cpd. **(4m)**. S39
Figure S37: ^{1}H NMR 8.5-6.5 ppm of cpd. **(4n)**. S40
Figure S38: ^{1}H NMR of cpd. **(4n)**. S41
Figure S39: ^{13}C NMR of cpd. **(4n)**. S42
Figure S40: ^{1}H NMR 8.5-6.5 ppm of cpd. **(4o)**. S43
Figure S41: ^{1}H NMR of cpd. **(4o)**. S44
Figure S42: ^{13}C NMR ppm of cpd. **(4o)**. S45
Figure S43: ^{1}H NMR 8.5-6.5 ppm of cpd. **(4p)**. S46
Figure S44: ^{1}H NMR 10.5-6.5 ppm of cpd. **(4p)**. S47
Figure S45: ^{1}H NMR of cpd. **(4p)**. S48
Figure S46: ^{13}C NMR of cpd. **(4p)**. S49
Figure S47: ^{1}H NMR 12-0 ppm of cpd. **(4c)**. S50
Figure S48: ^{13}C NMR 200-0 ppm of cpd. **(4c)**. S51
Figure S49: ^{1}H NMR 12-0 ppm of cpd. **(4e)**. S52
Figure S50: ^{13}C NMR 200-0 ppm of cpd. **(4e)**. S53
Figure S51: ^{1}H NMR 12-0 ppm of cpd. **(4f)**. S54
Figure S52: ^{1}H NMR 12-0 ppm of cpd. **(4o)**. S55
Table S1: The optimization Microwave irradiation condition for Synthesis of **4a-p**. S56

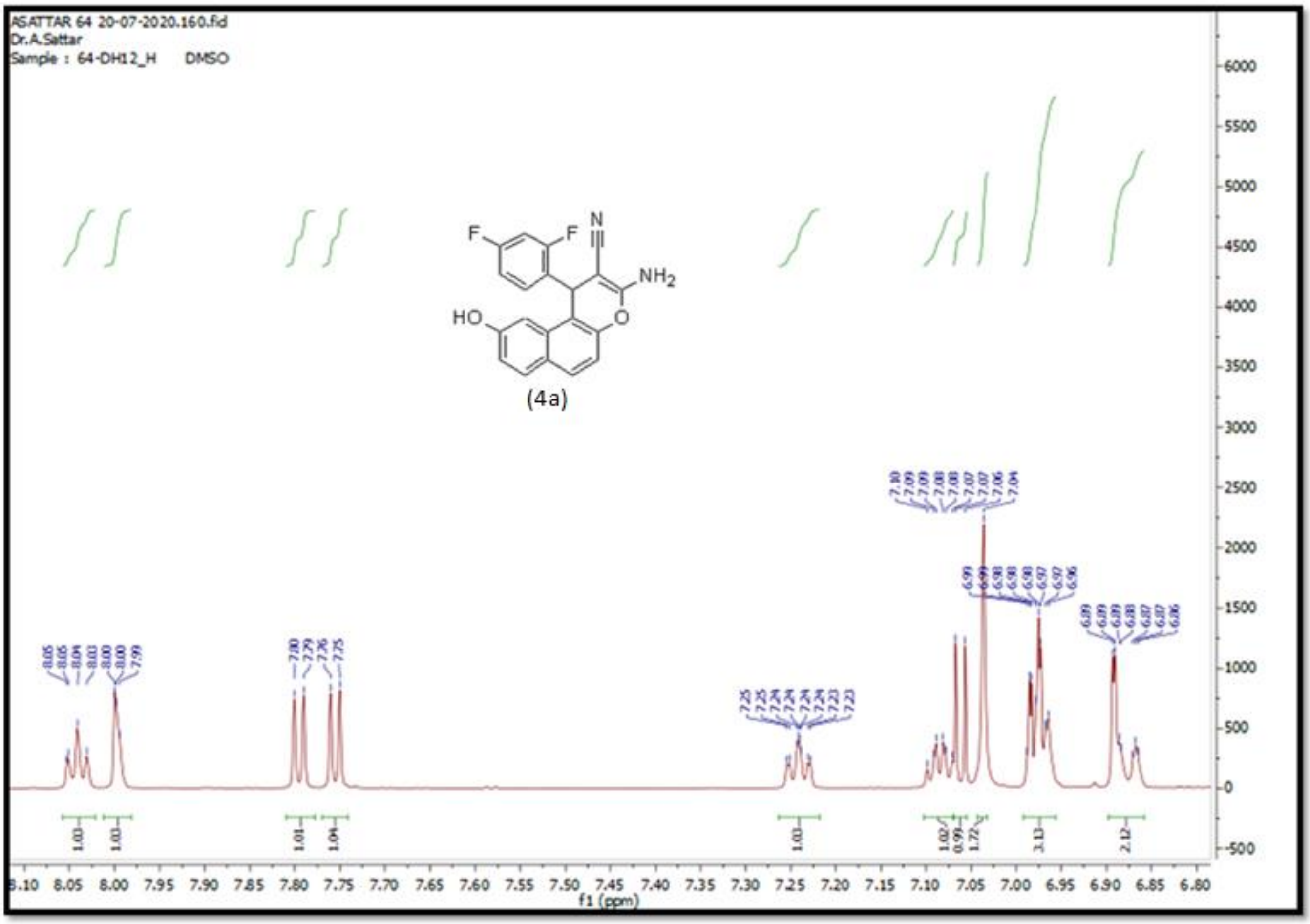

Figure S1: ^{1}H NMR 8.5-6.5 ppm of cpd. **(4a)**.

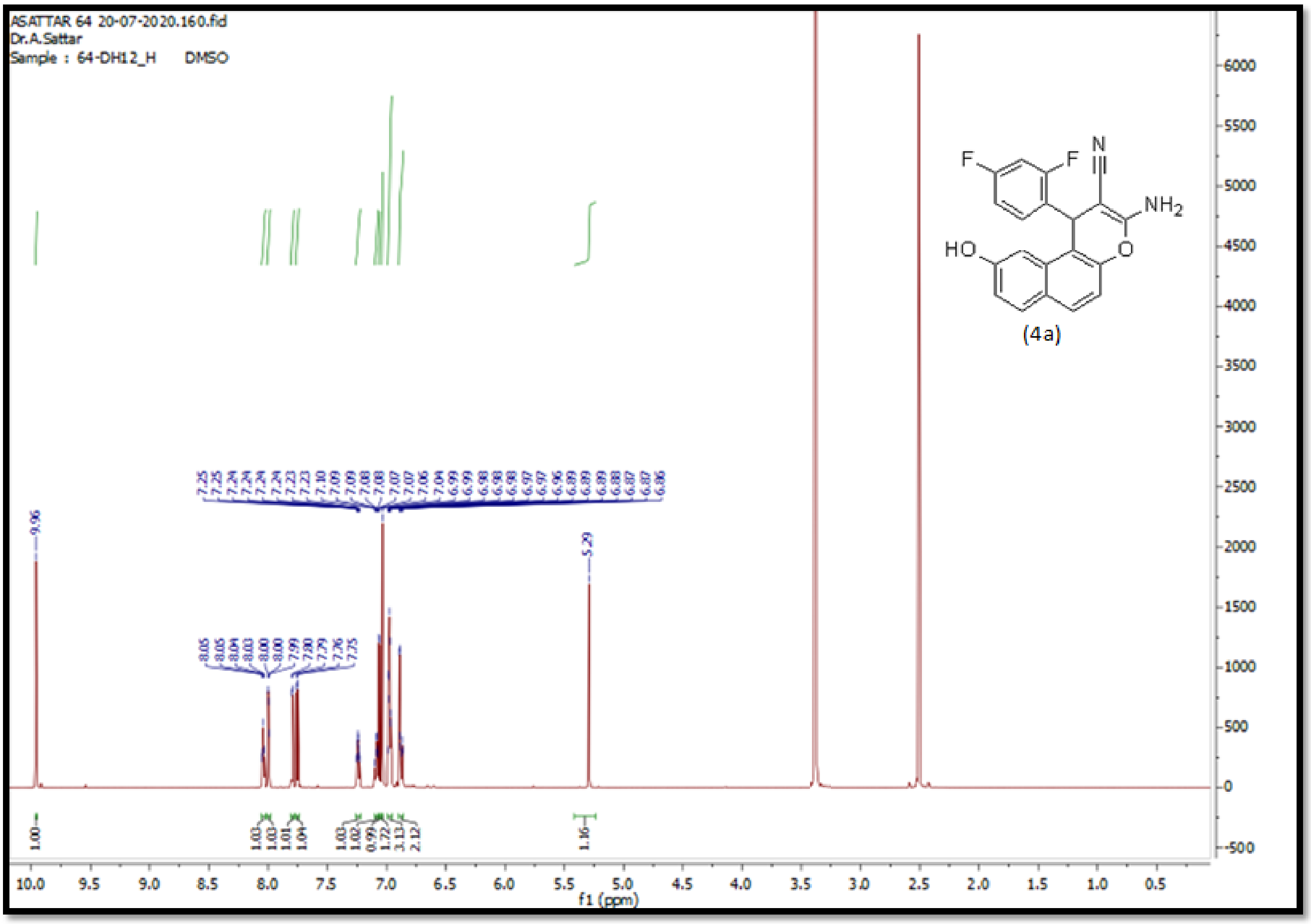

Figure S2: ^{1}H NMR of cpd. **(4a).**

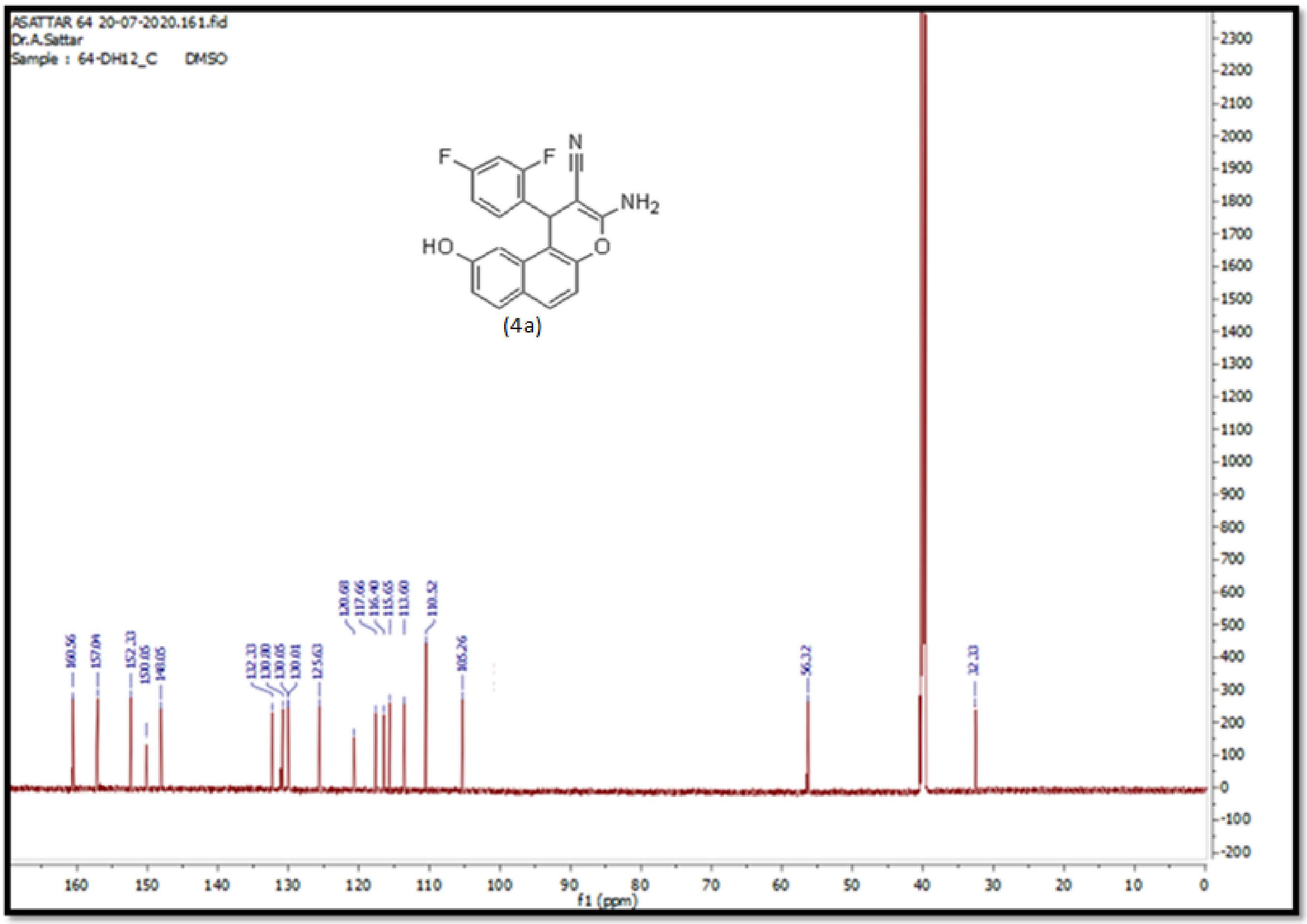

Figure S3: ^{13}C NMR of cpd. **(4a)**.

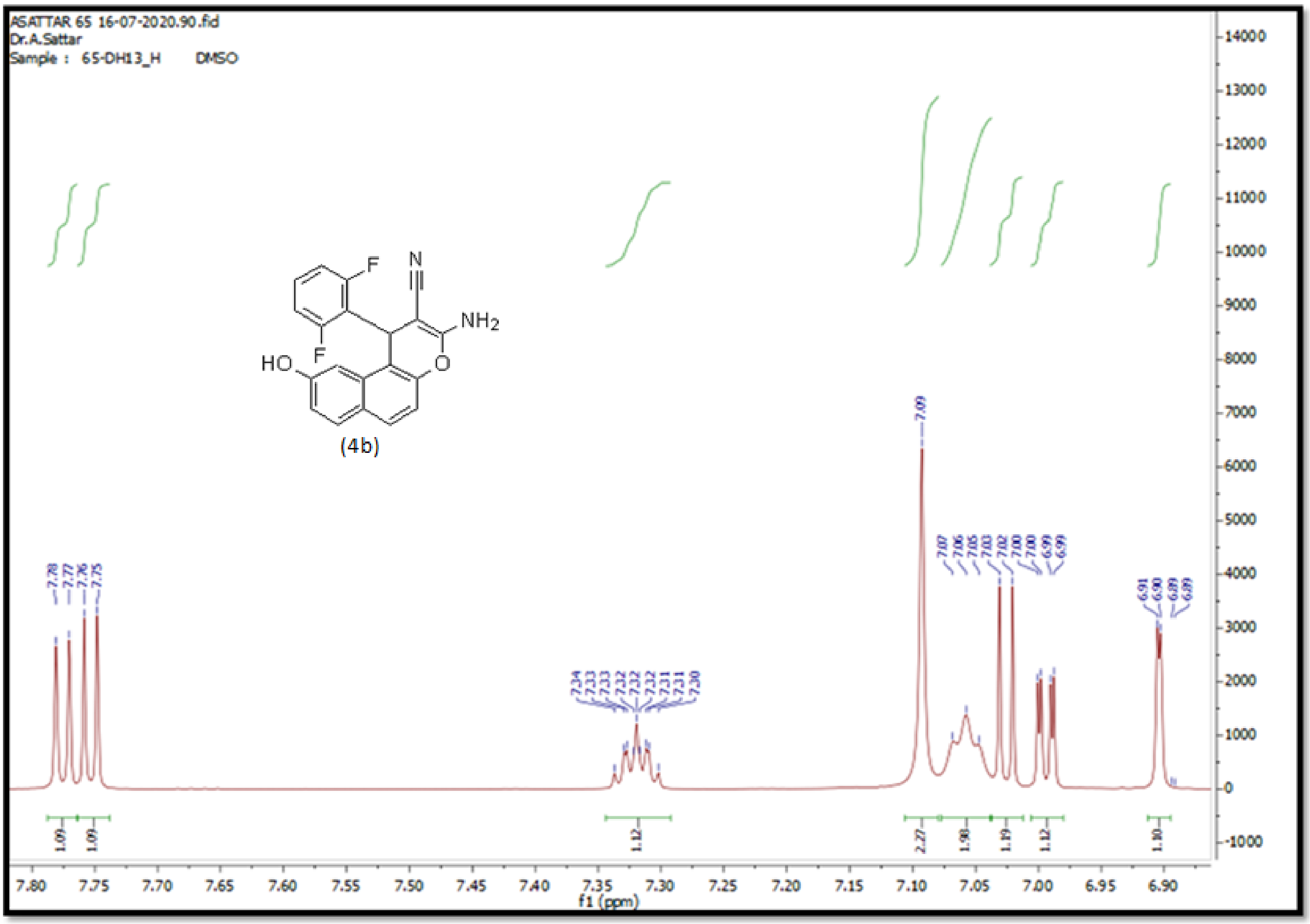

Figure S4: ^{1}H NMR 8.5-6.5 ppm of cpd. **(4b)**.

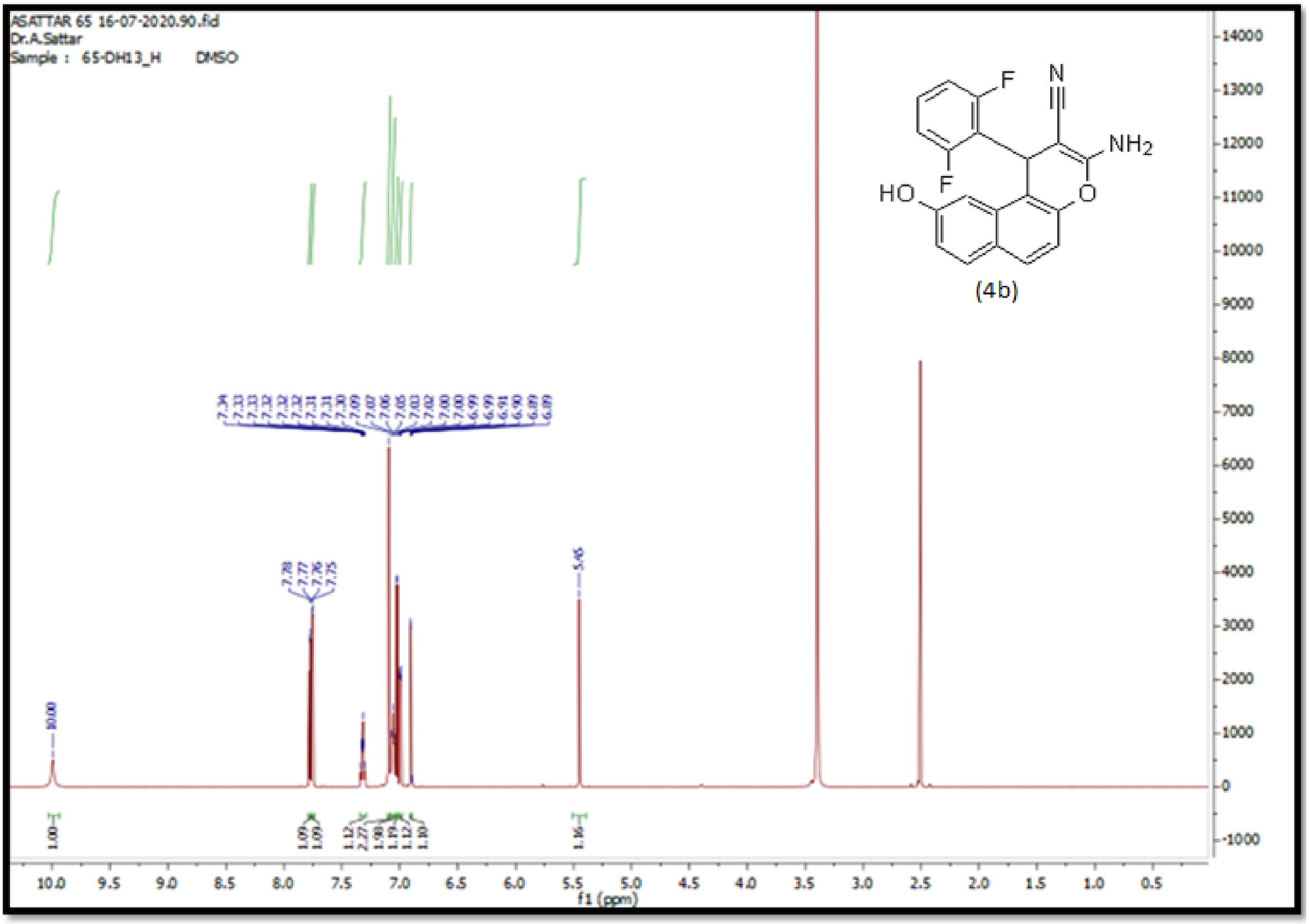

Figure S5: ^{1}H NMR of cpd. **(4b)**.

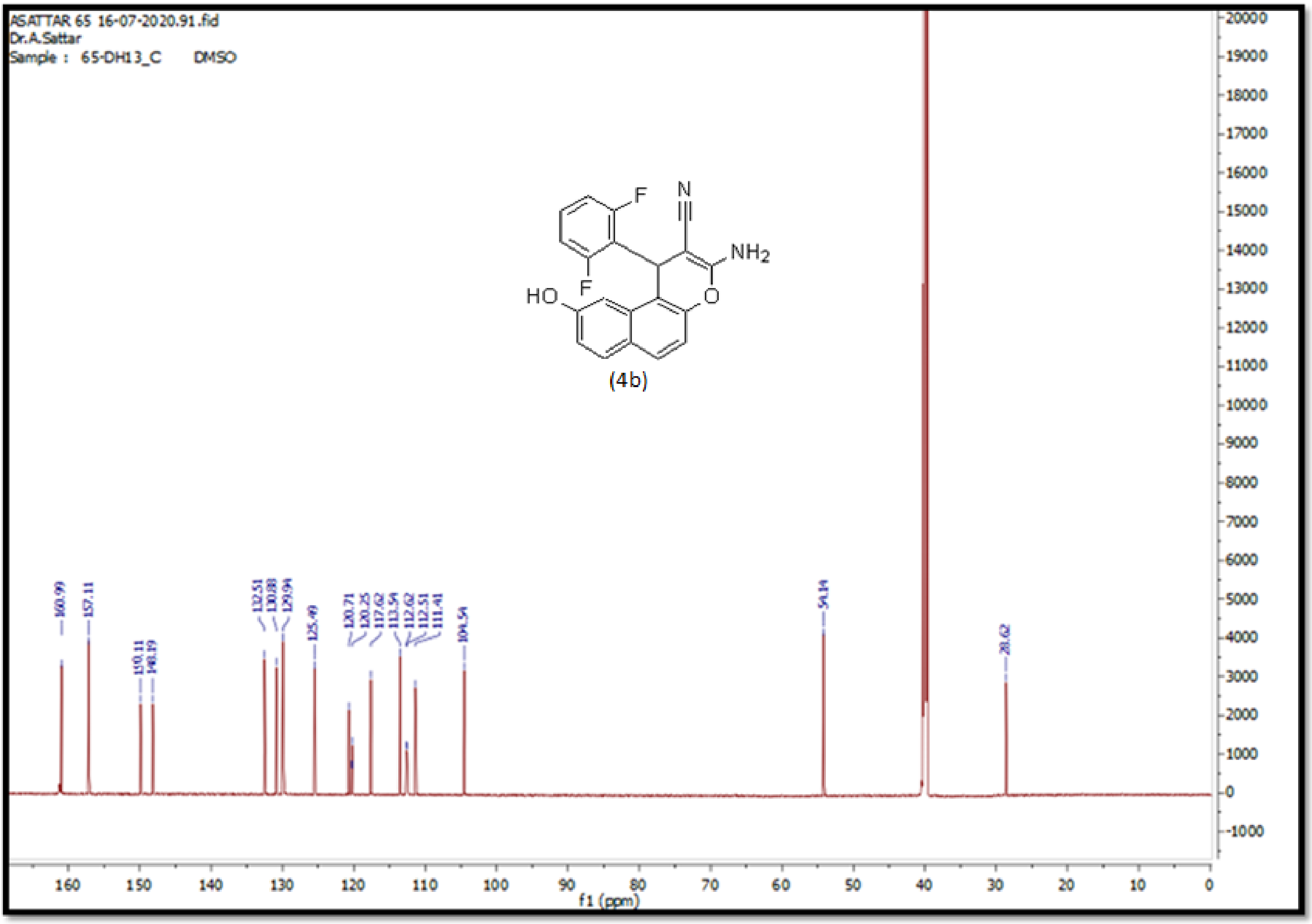

Figure S6: ^{13}C NMR of cpd. **(4b)**.

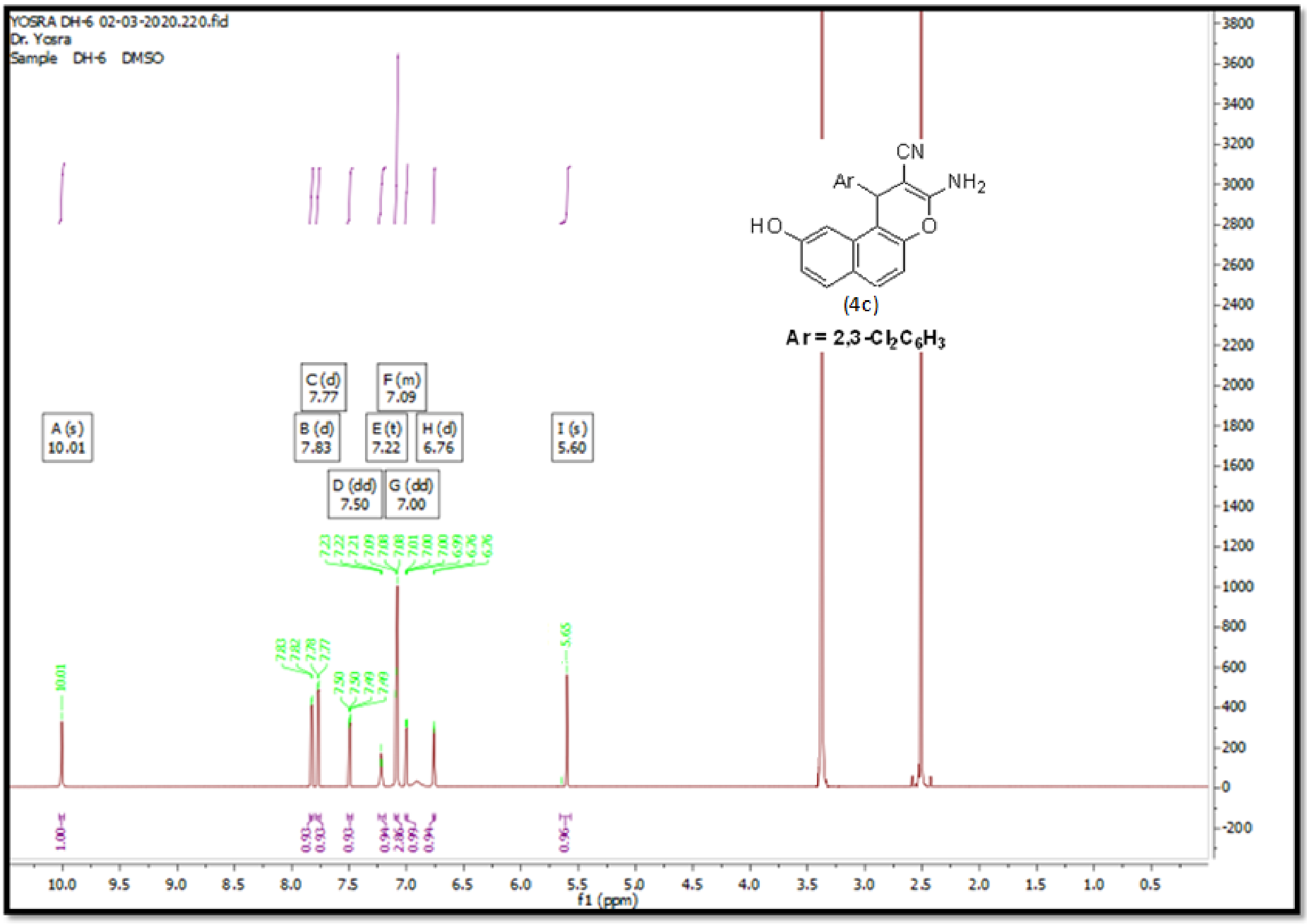

Figure S7: ^{1}H NMR of cpd. **(4c)**.

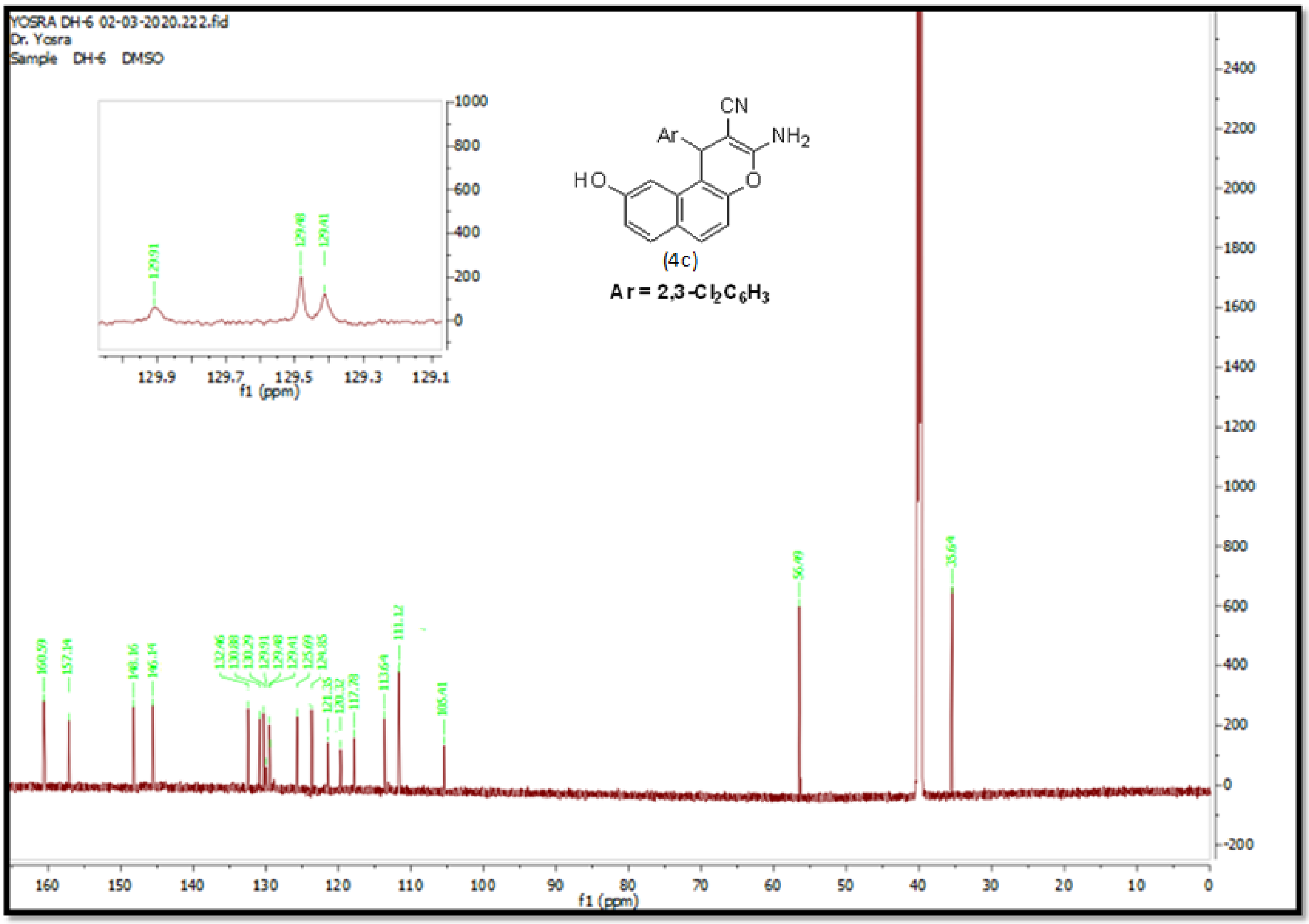

Figure S8: ^{13}C NMR of cpd. **(4c)**.

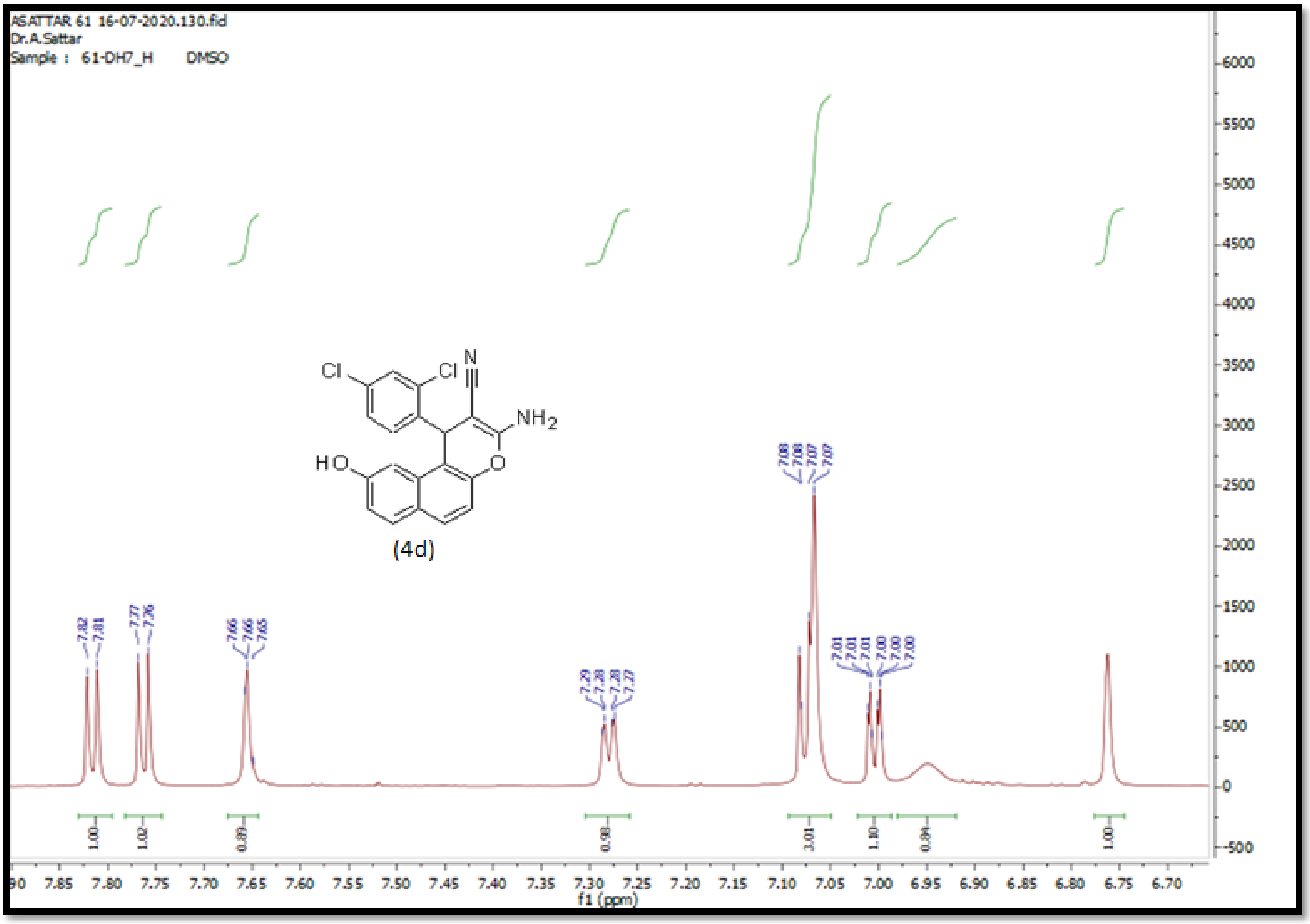

Figure S9: ^{1}H NMR 8.5-6.5 ppm of cpd. **(4d)**.

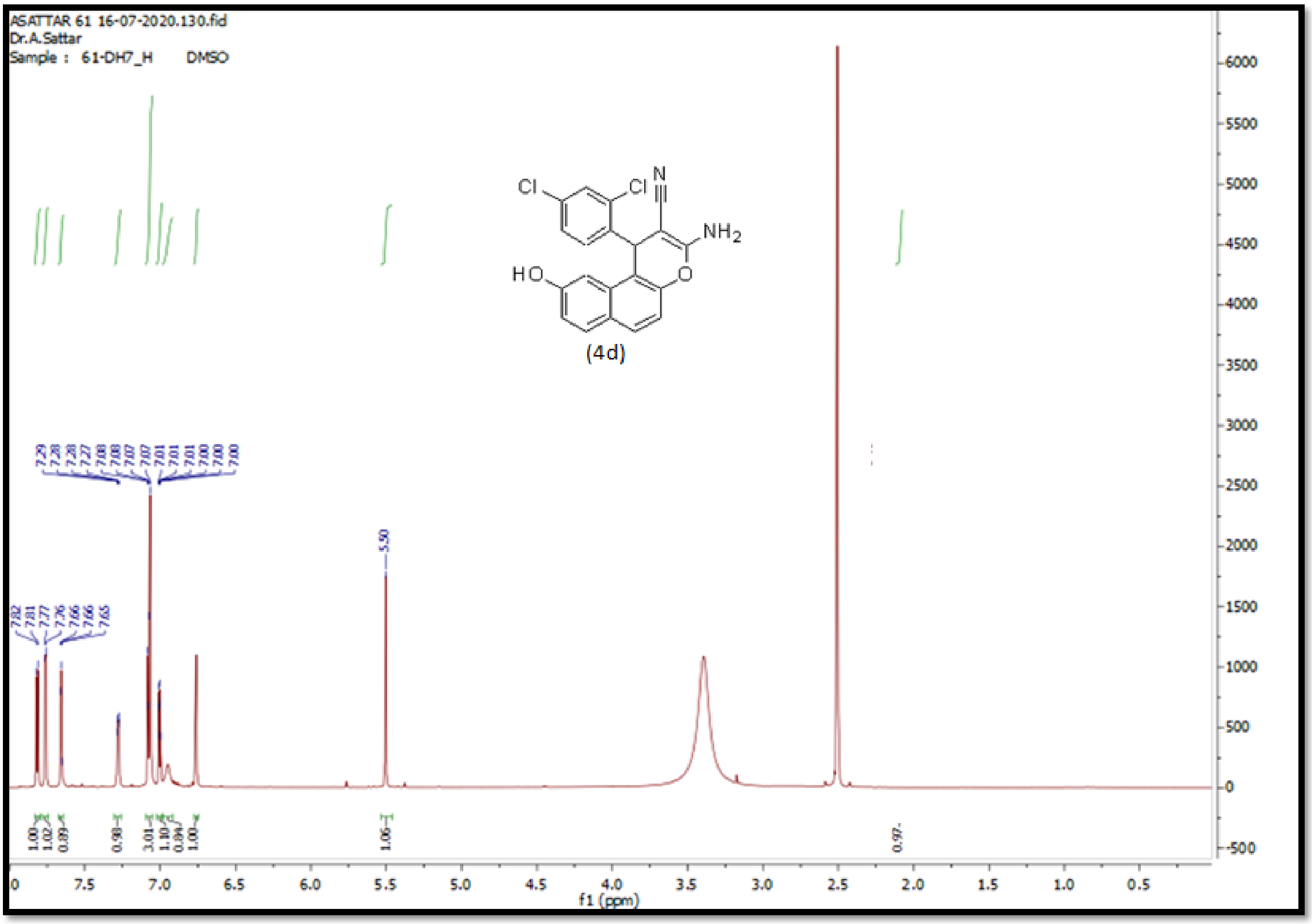

Figure S10: ^{1}H NMR of cpd **(4d)**.

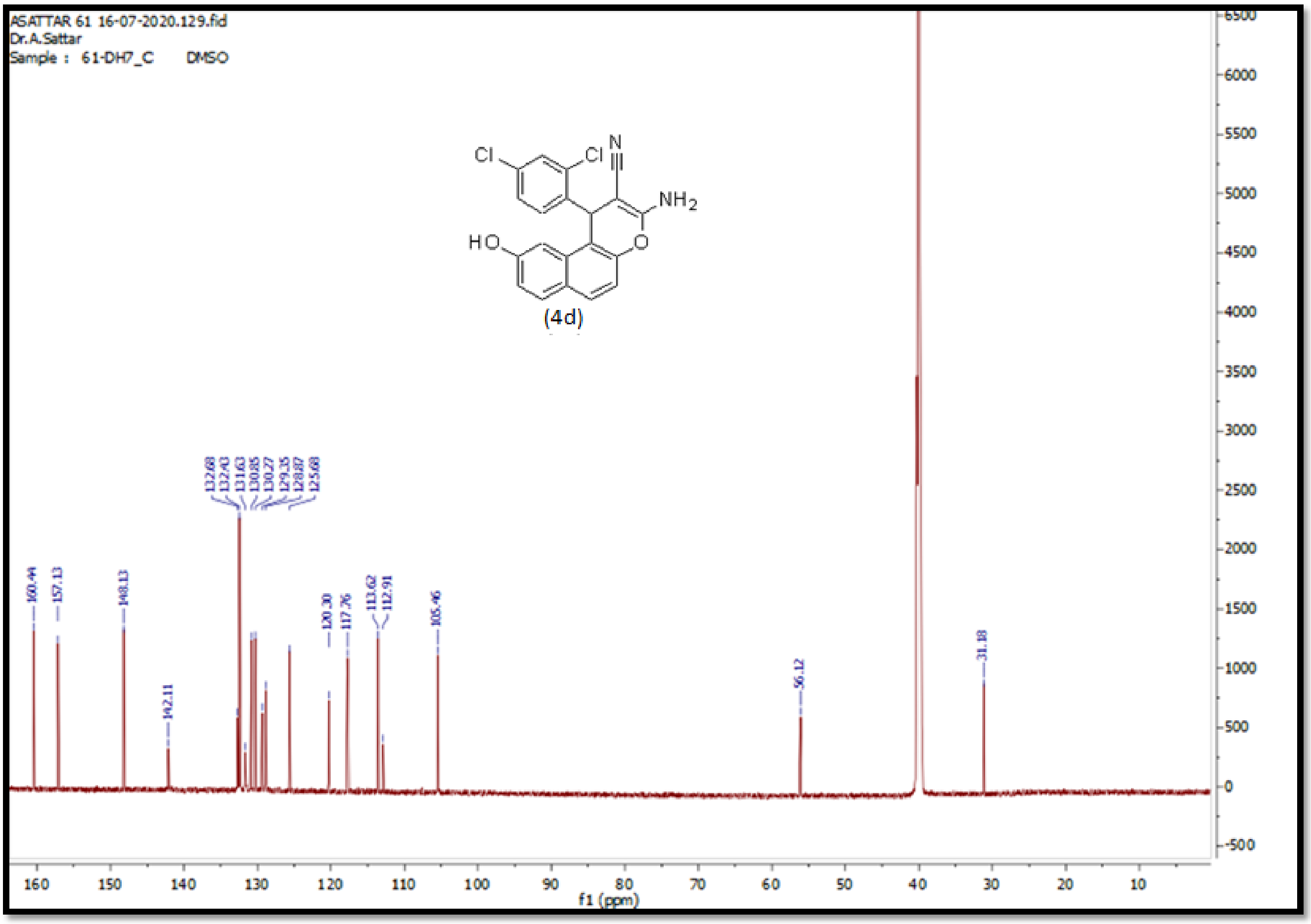

Figure S11: ^{13}C NMR of cpd. **(4d)**.

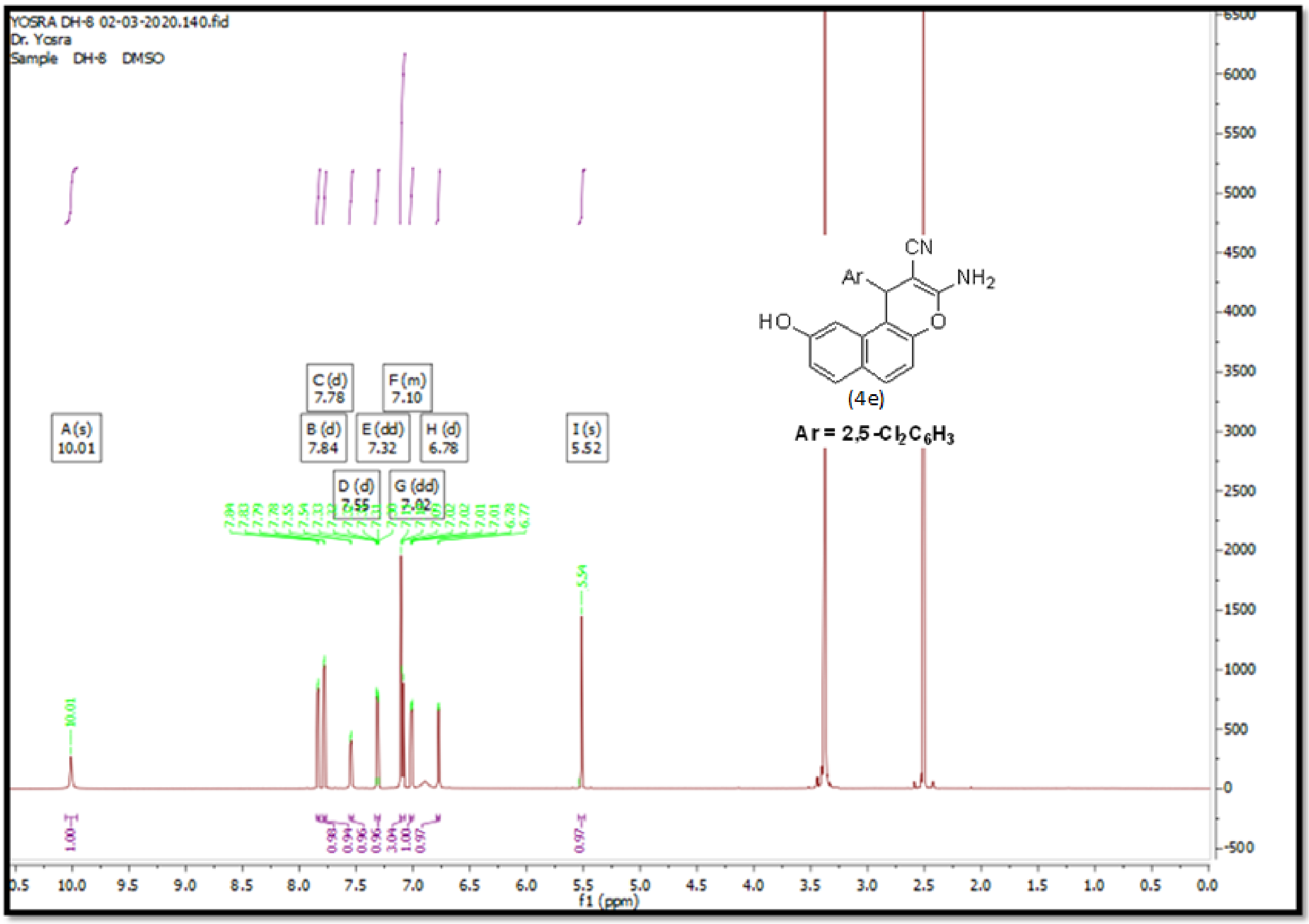

Figure S12: ^{1}H NMR of cpd **(4e)**.

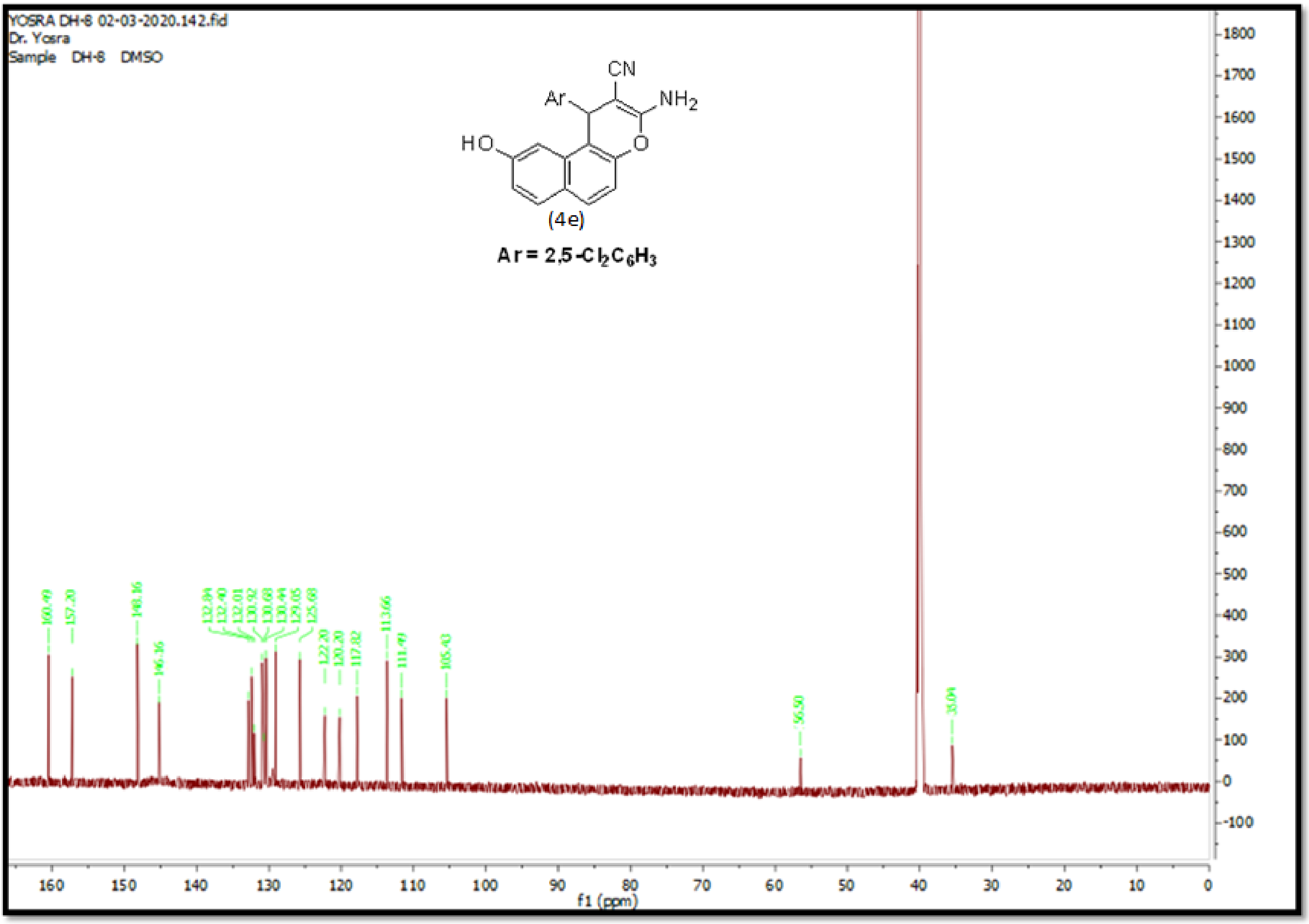

Figure S13: ^{13}C NMR of cpd. **(4e)**.

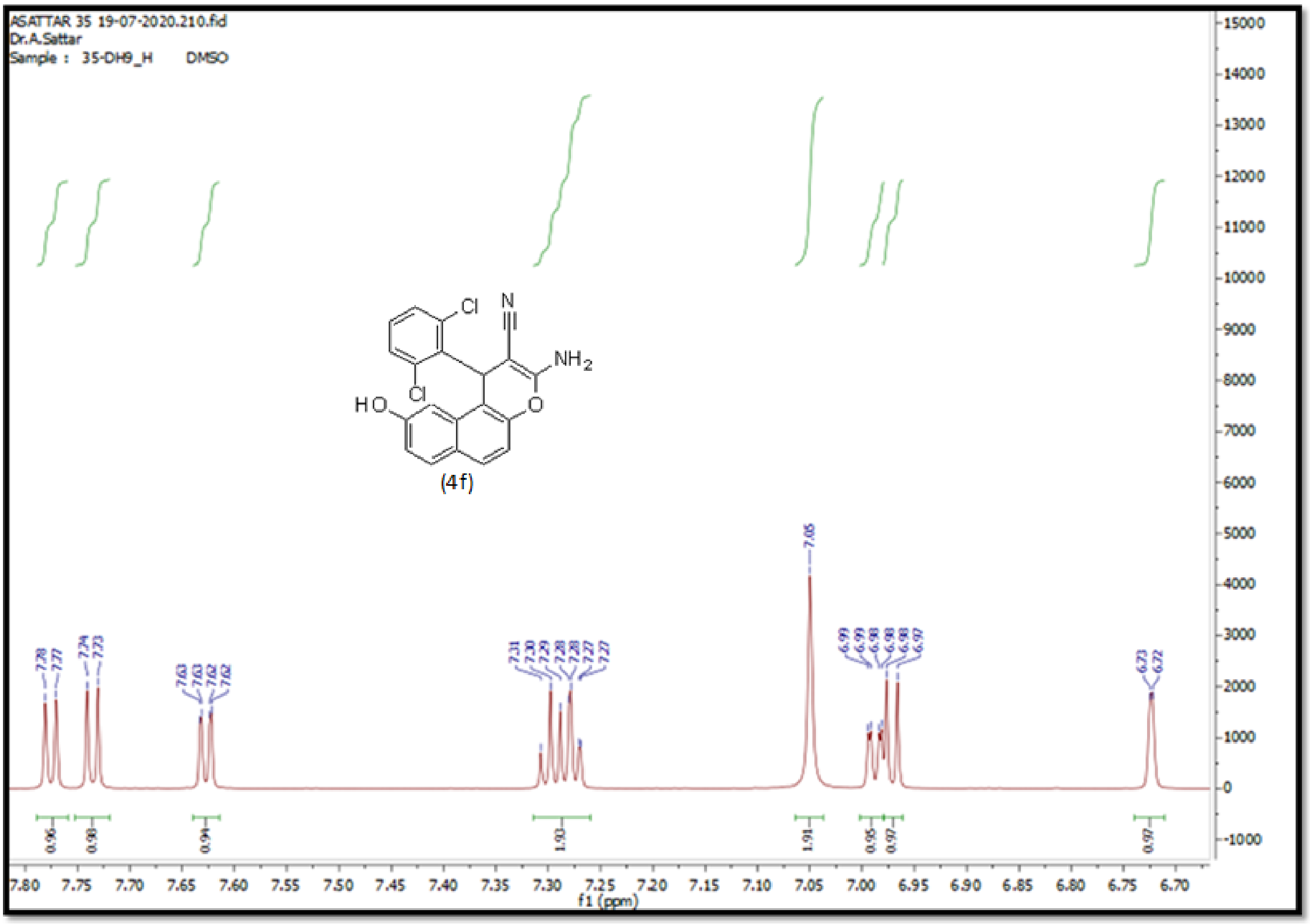

Figure S14: ^{1}H NMR 8.5-6.5 ppm of cpd. **(4f)**.

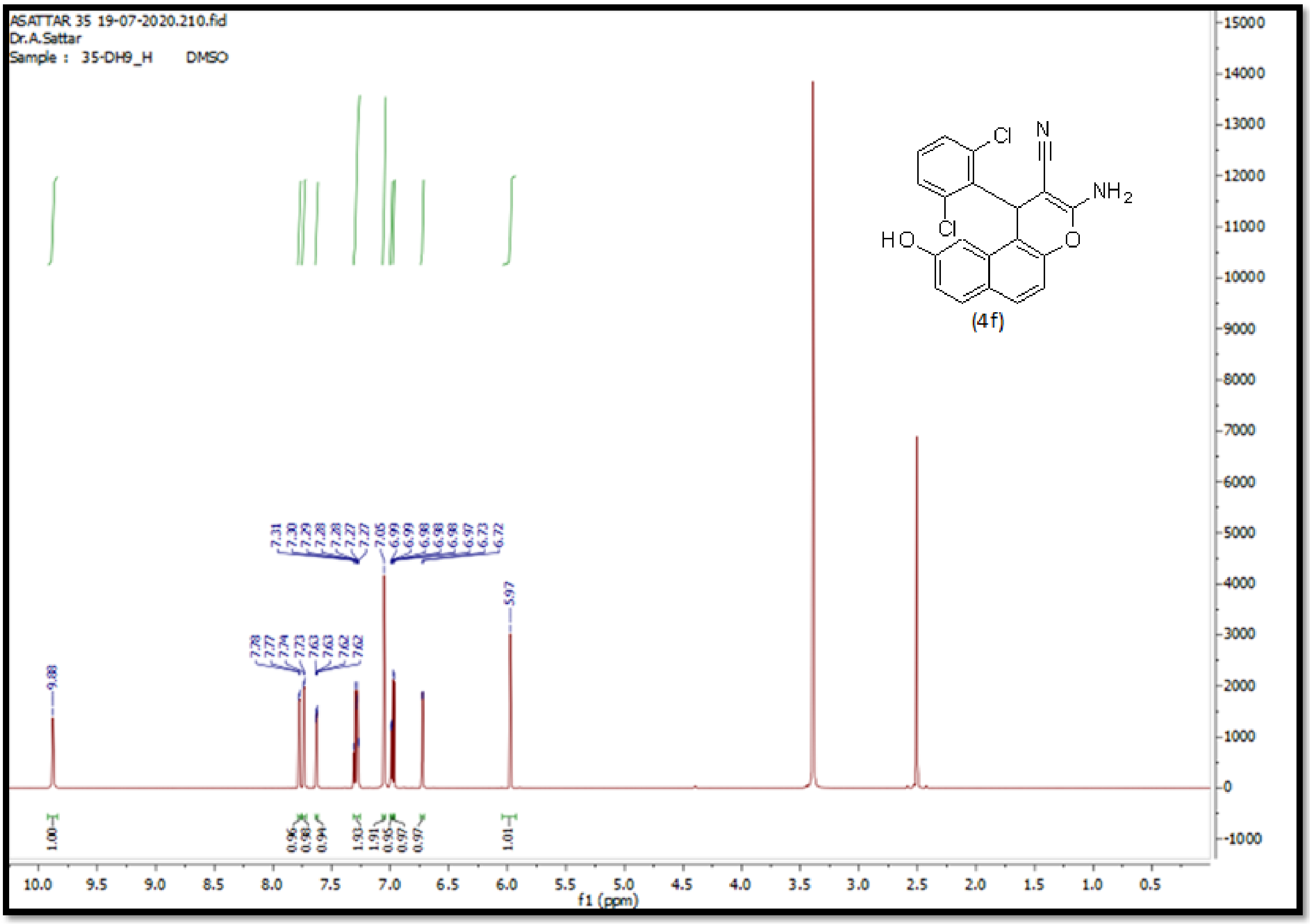

Figure S15: ^{1}H NMR of cpd. **(4f)**.

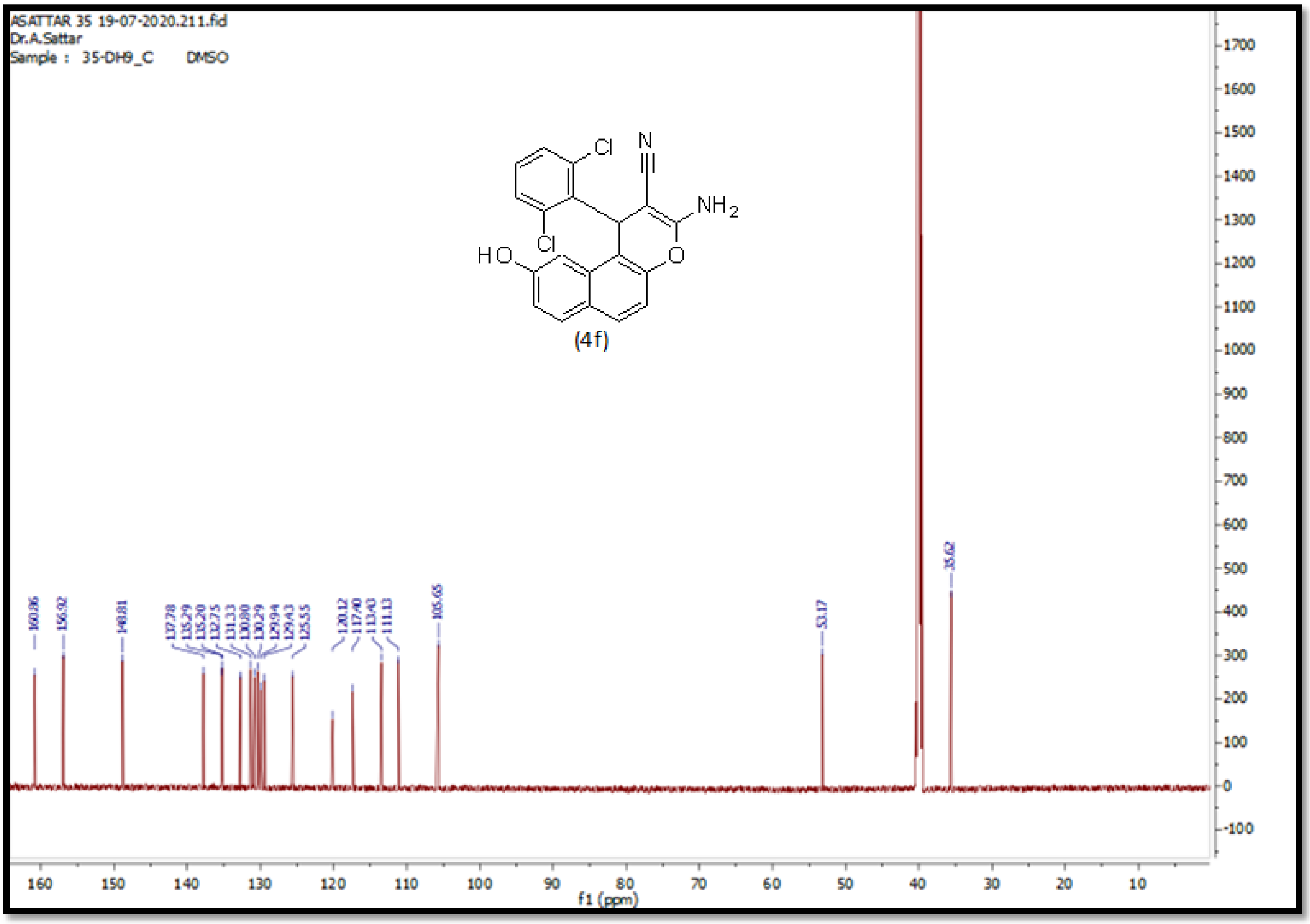

Figure S16: ^{13}C NMR of cpd. **(4f)**.

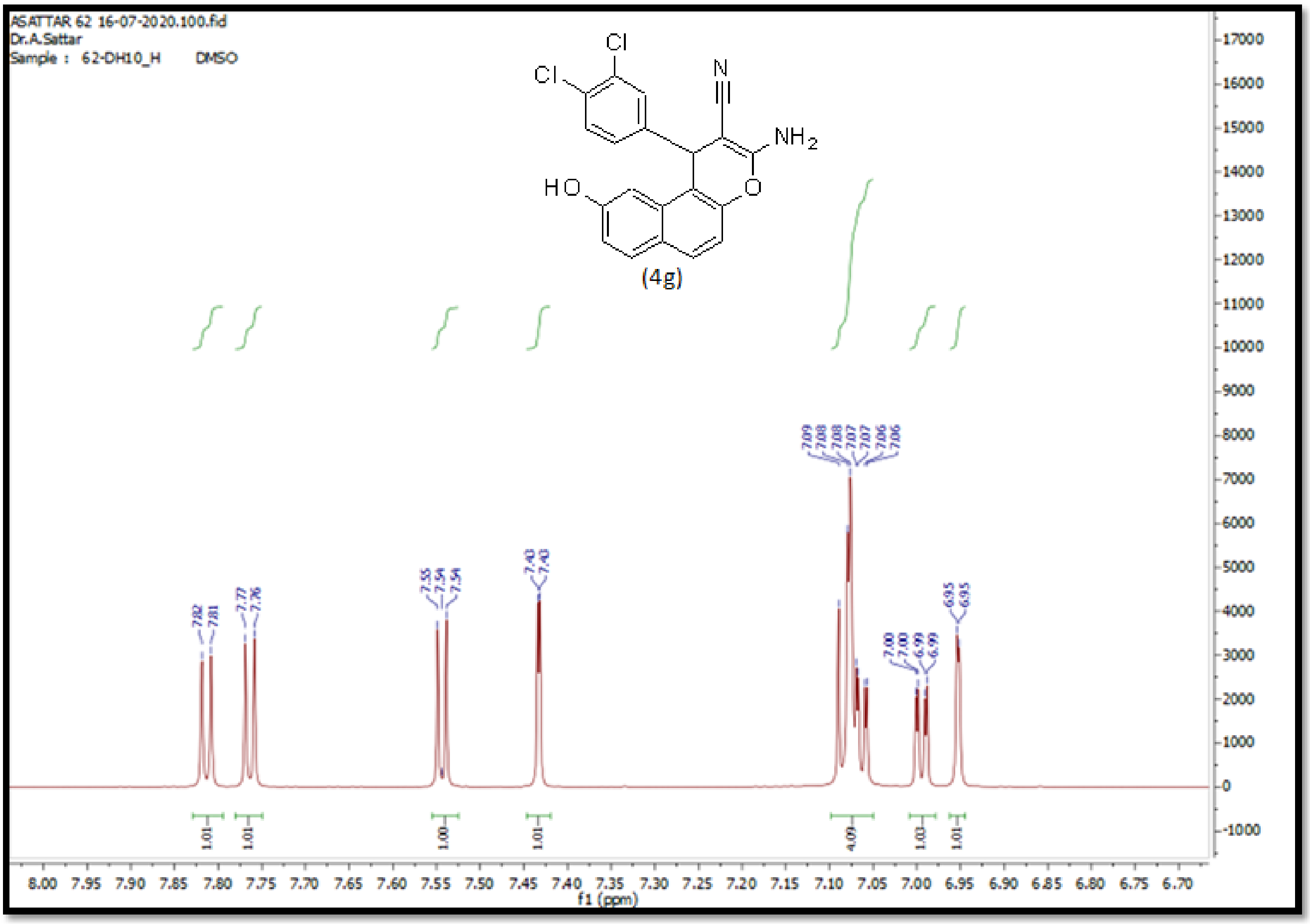

Figure S17: ^{1}H NMR 8.5-6.5 ppm of cpd. **(4g)**.

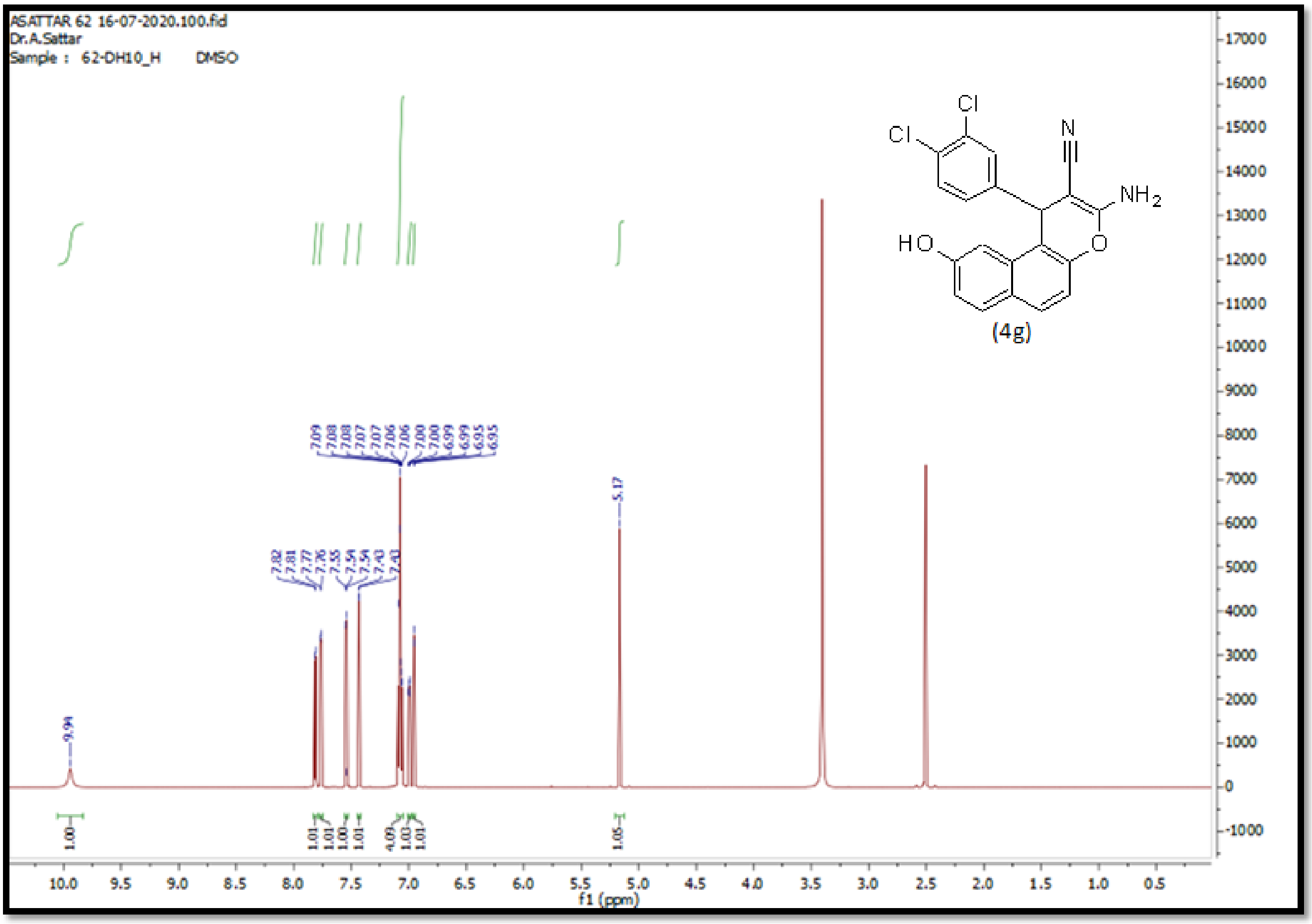

Figure S18: ^{1}H NMR of cpd. **(4g)**.

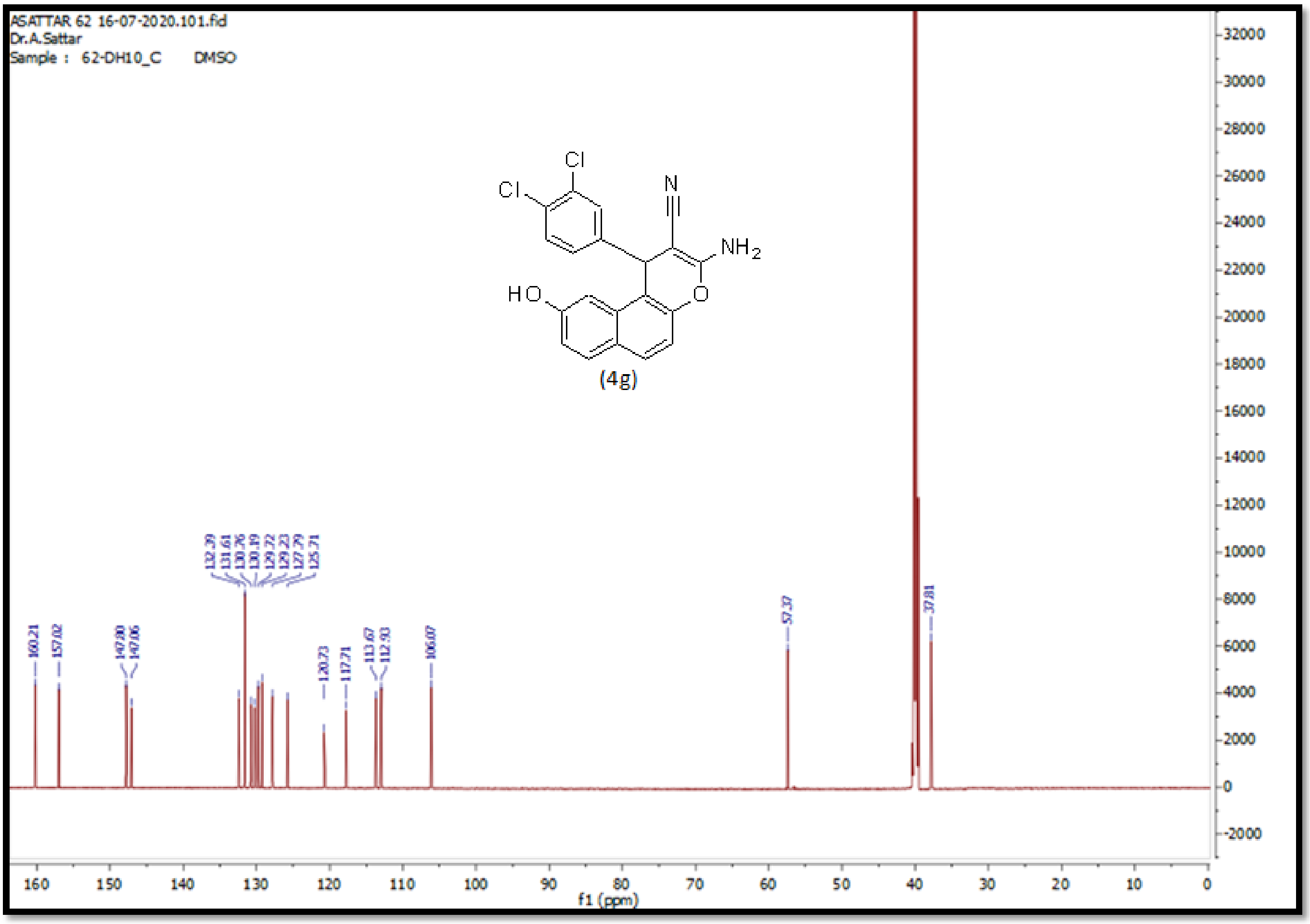

Figure S19: ^{13}C NMR of cpd. **(4g)**.

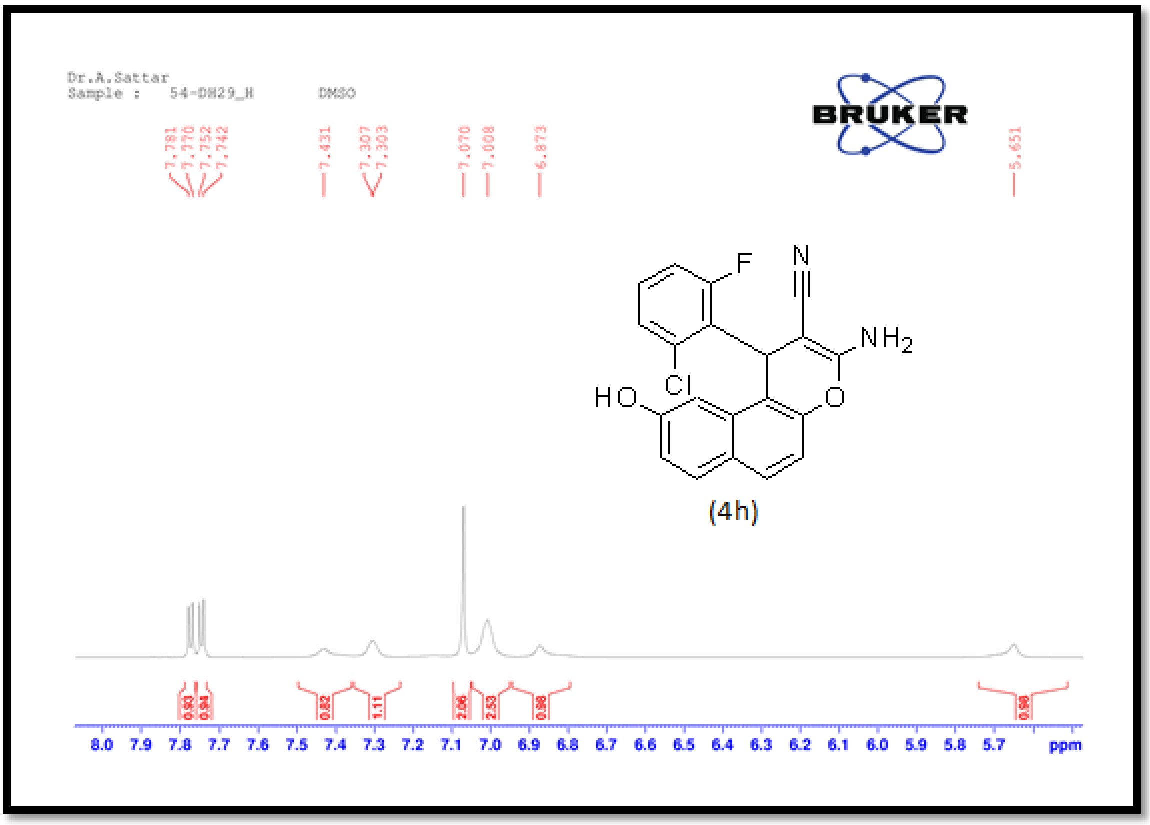

Figure S20: ^{1}H NMR 8.5-6.5 ppm of cpd. **(4h)**.

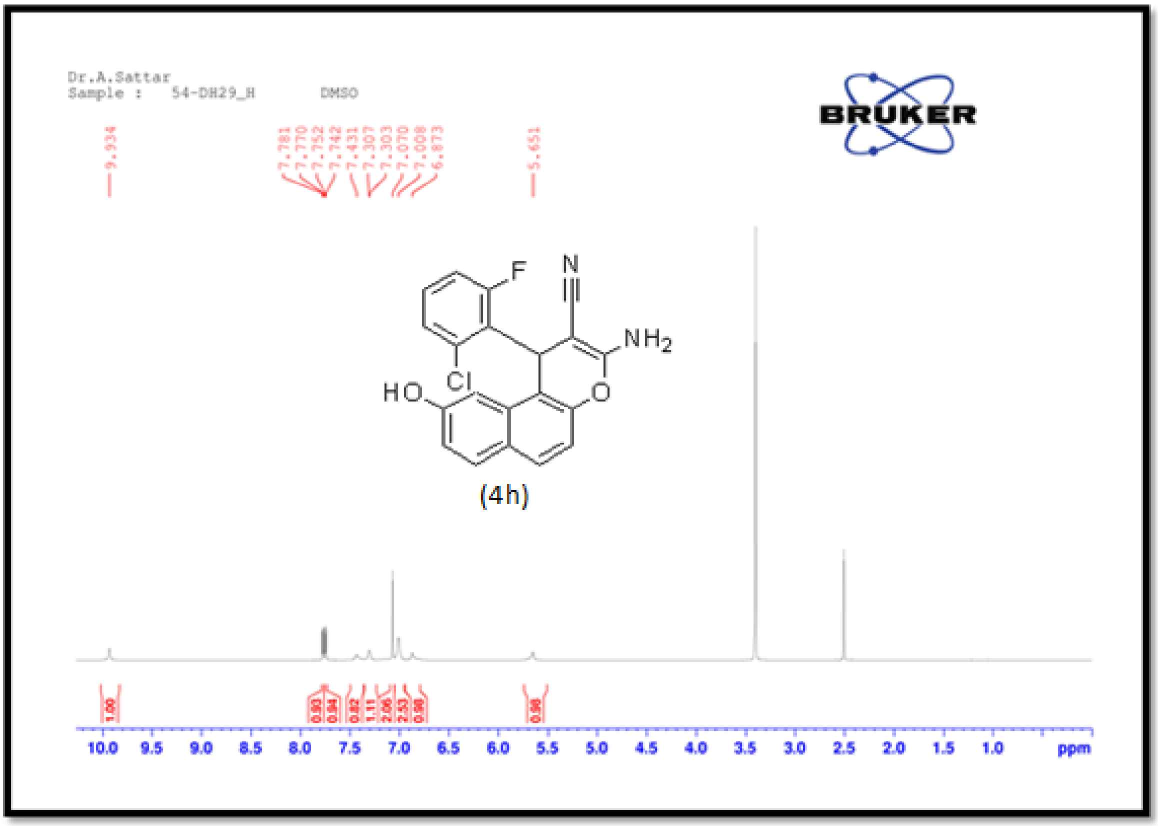

Figure S21: ^{1}H NMR of cpd. **(4h)**.

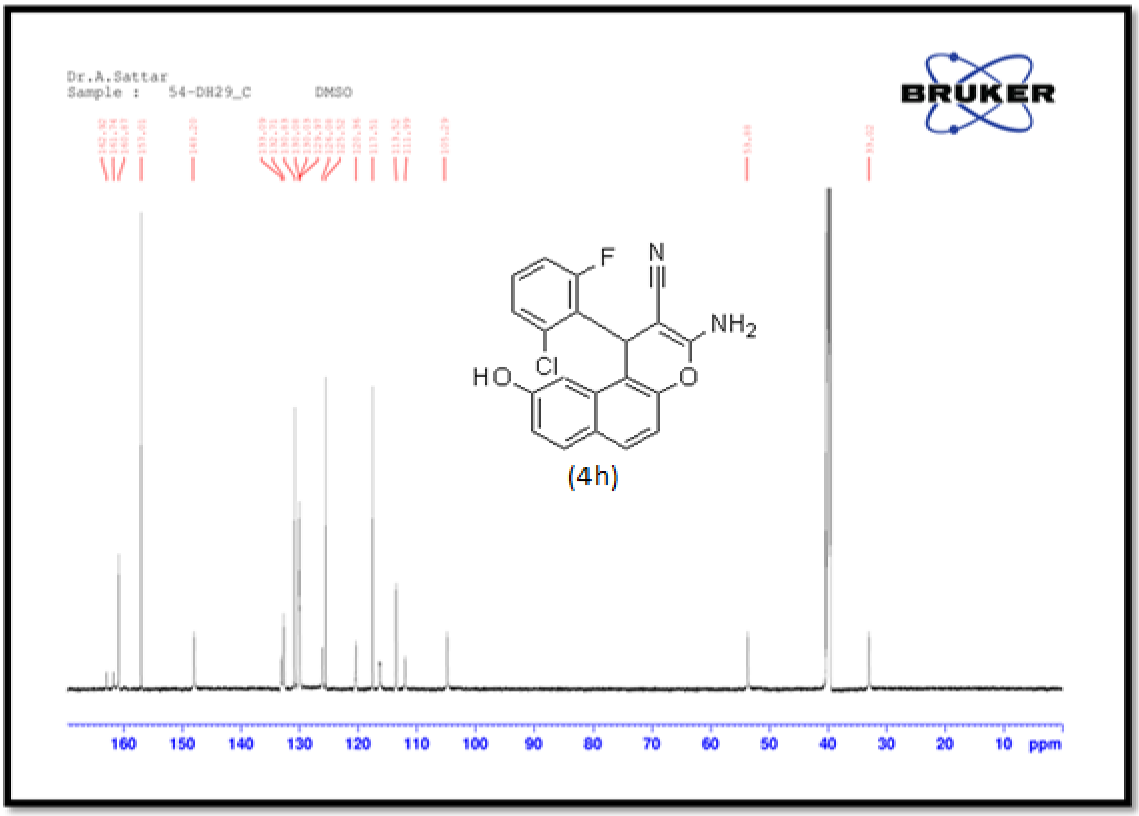

Figure S22: ^{13}C NMR of cpd. **(4h)**.

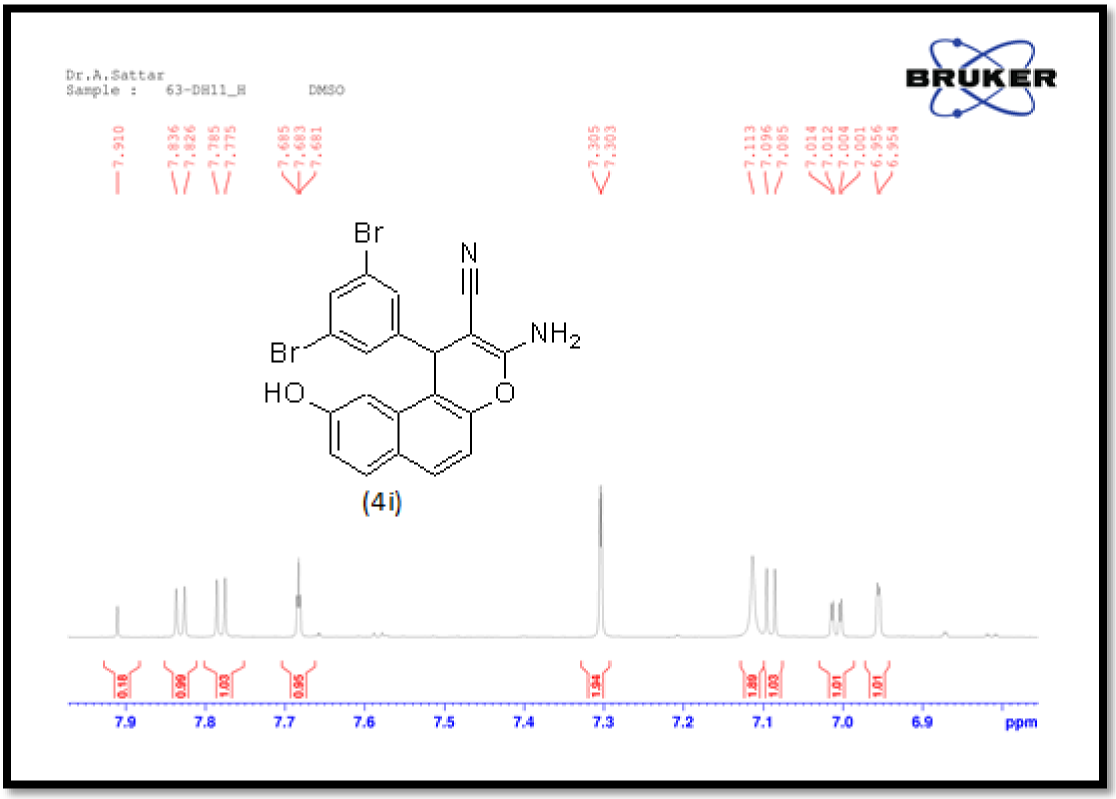

Figure S23: ^{1}H NMR 8.5-6.5 ppm of cpd. **(4i)**.

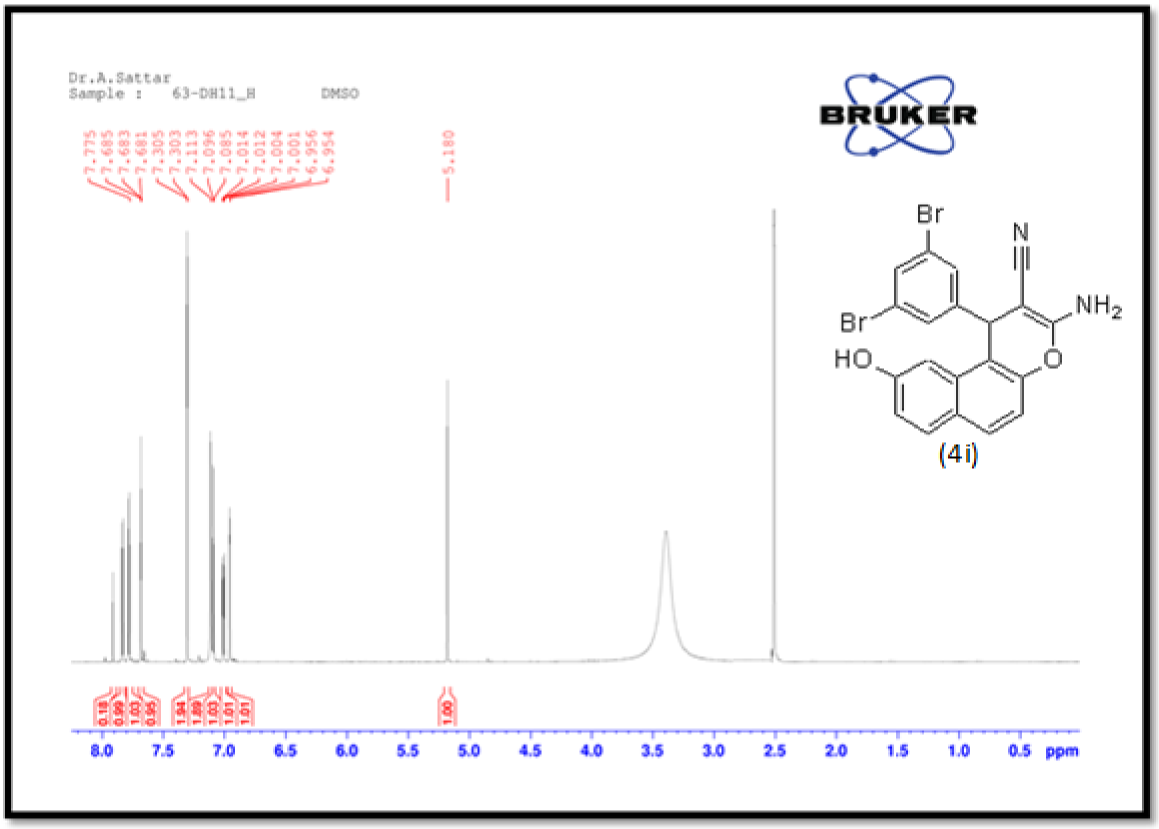

Figure S24: ^{1}H NMR of cpd. **(4i)**.

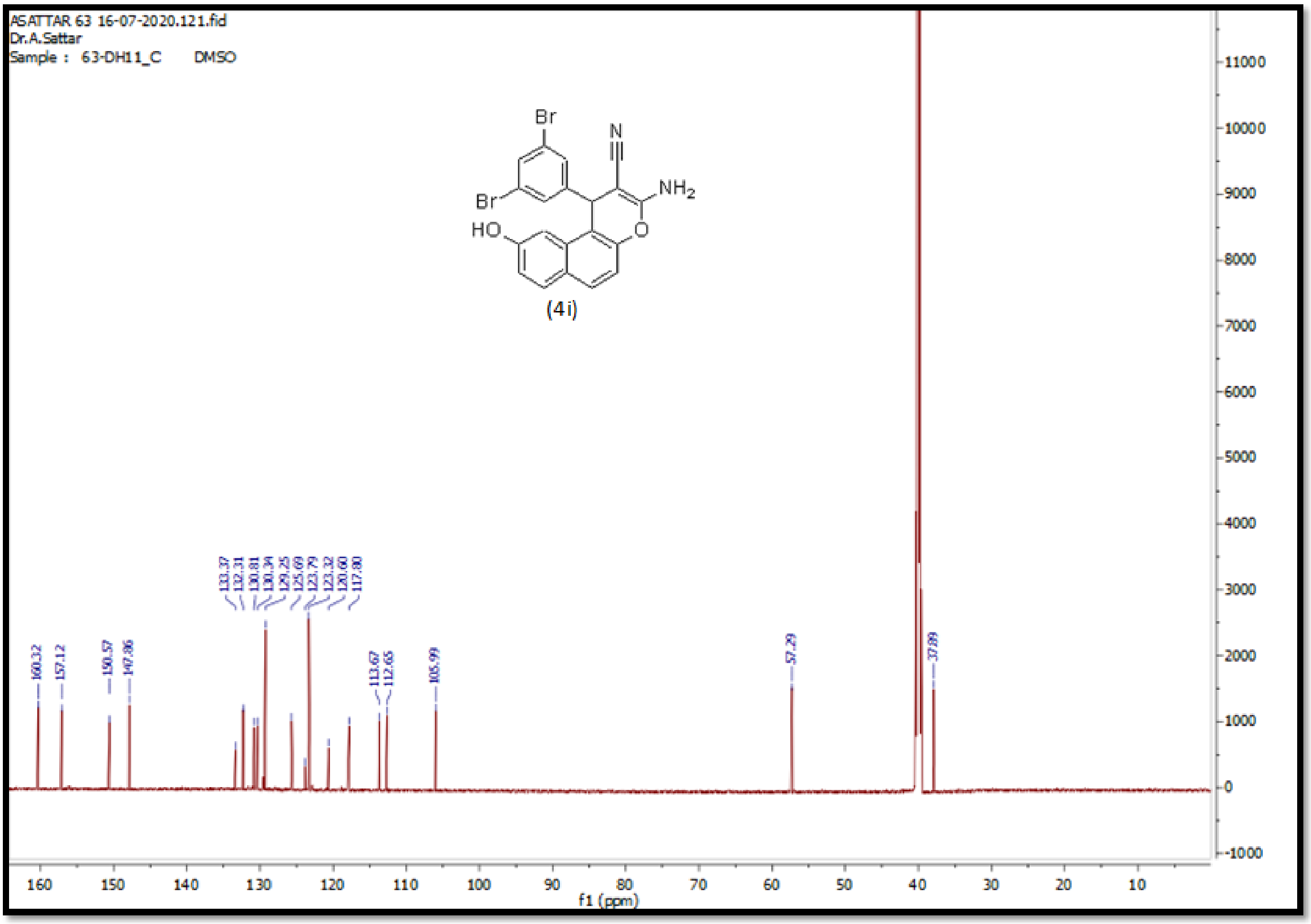

Figure S25: ^{13}C NMR of cpd. **(4i)**.

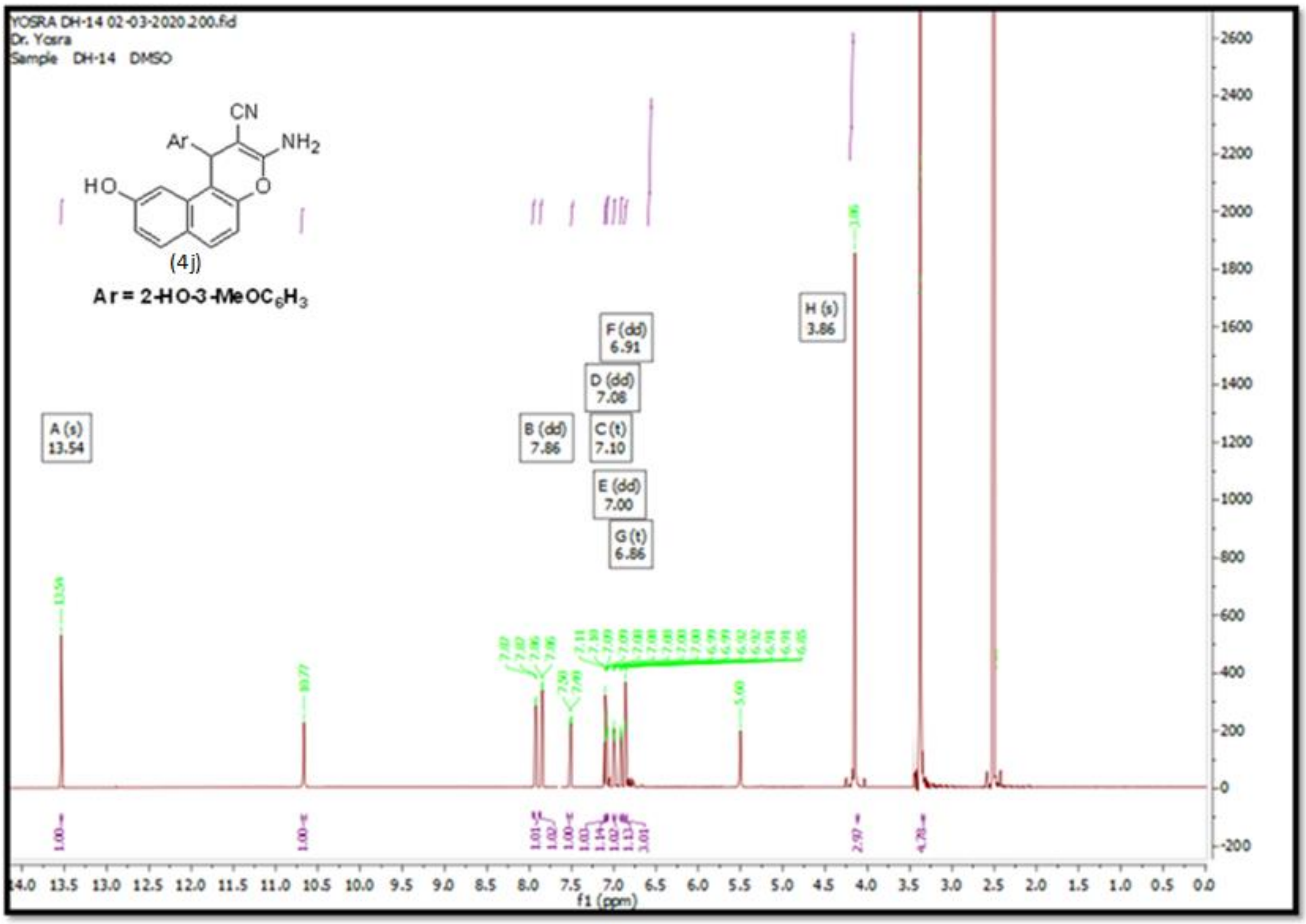

Figure S26: ^{1}H NMR of cpd. **(4j)**.

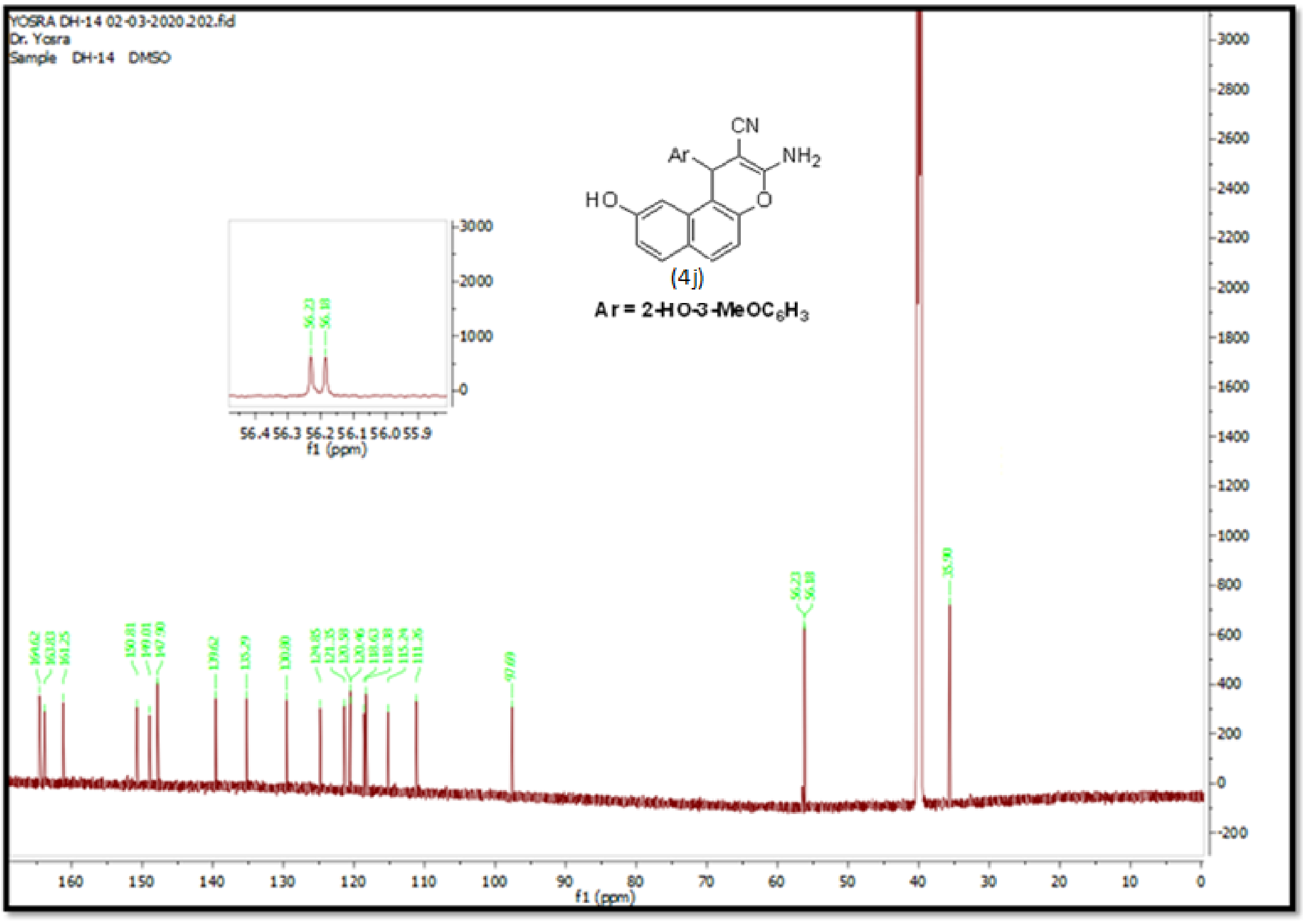

Figure S27: ^{13}C NMR of cpd. **(4j)**.

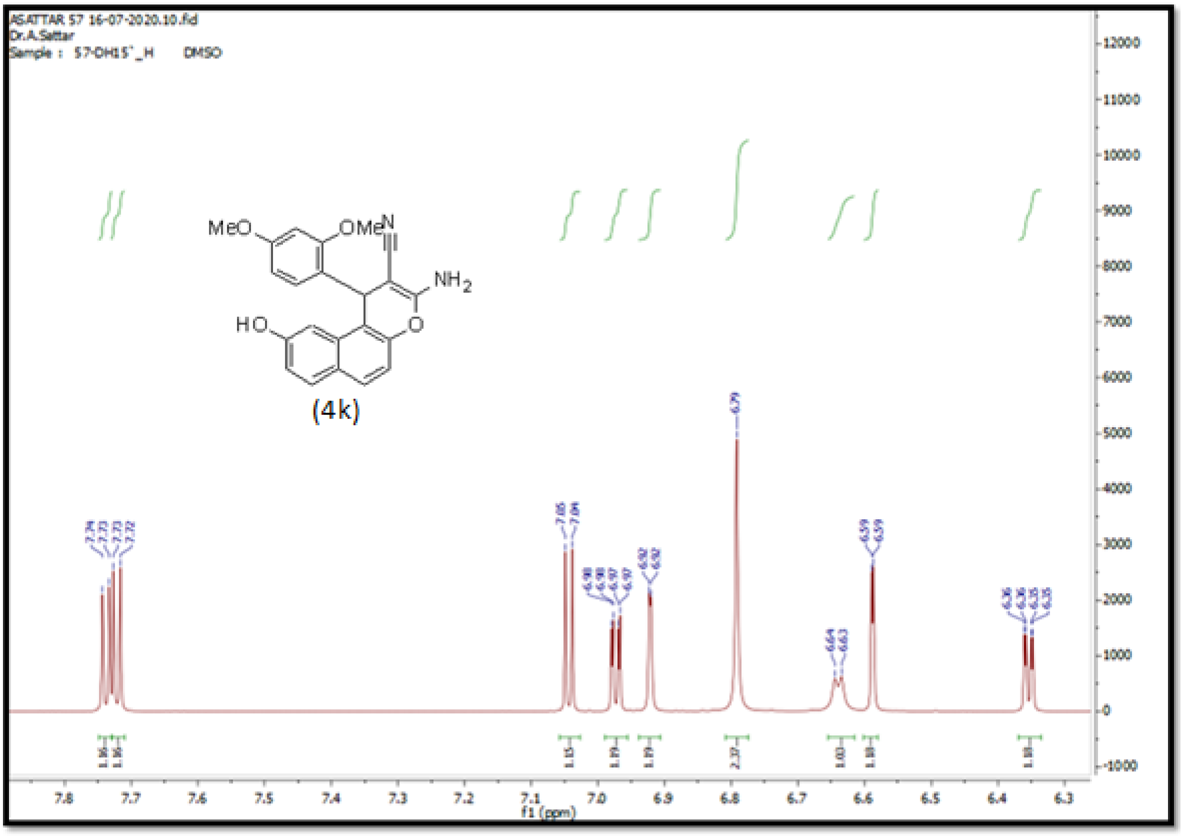

Figure S28: ^{1}H NMR 8.5-6.5 ppm of cpd. **(4k)**.

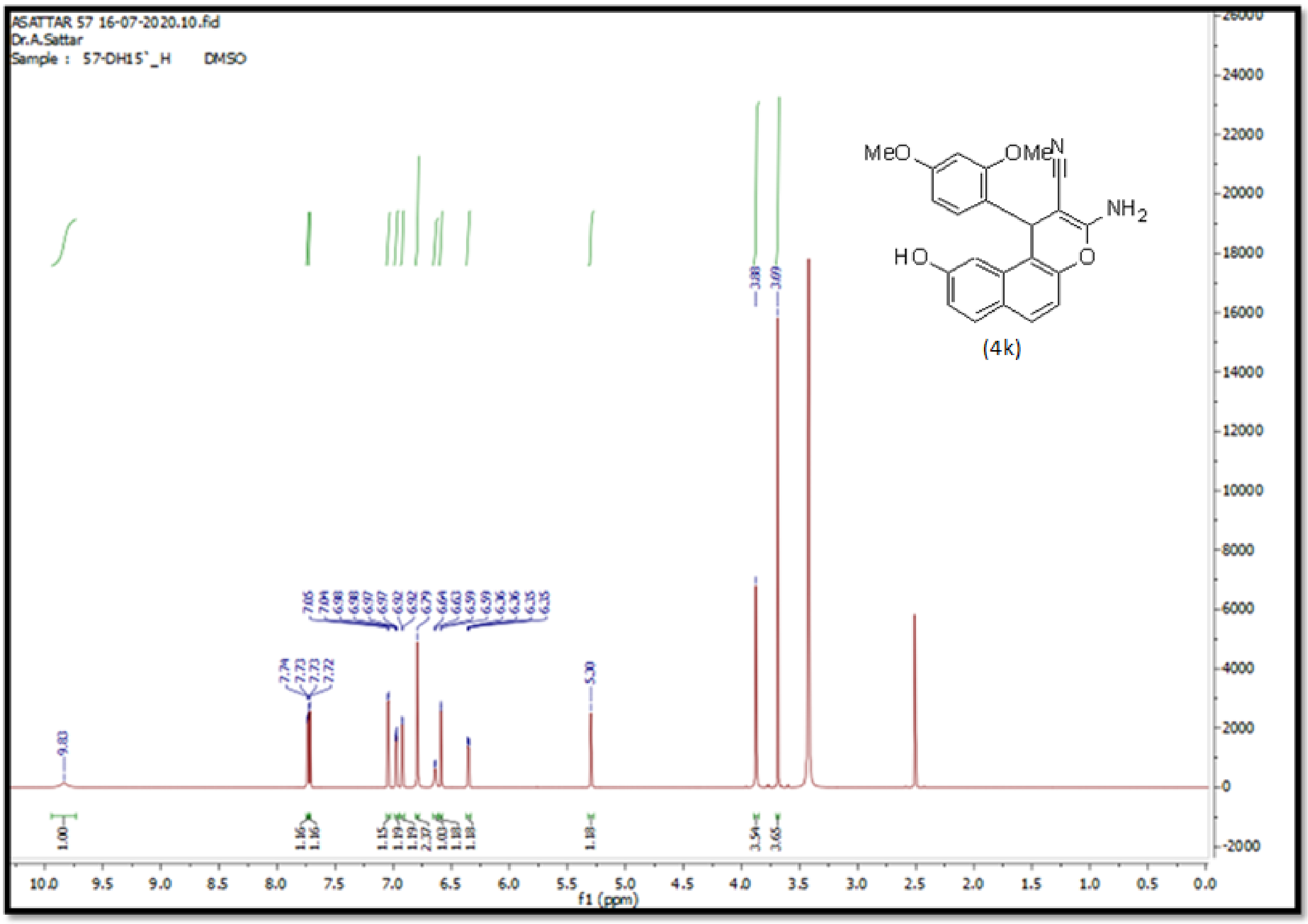

Figure S29: ^{1}H NMR of cpd. **(4k)**.

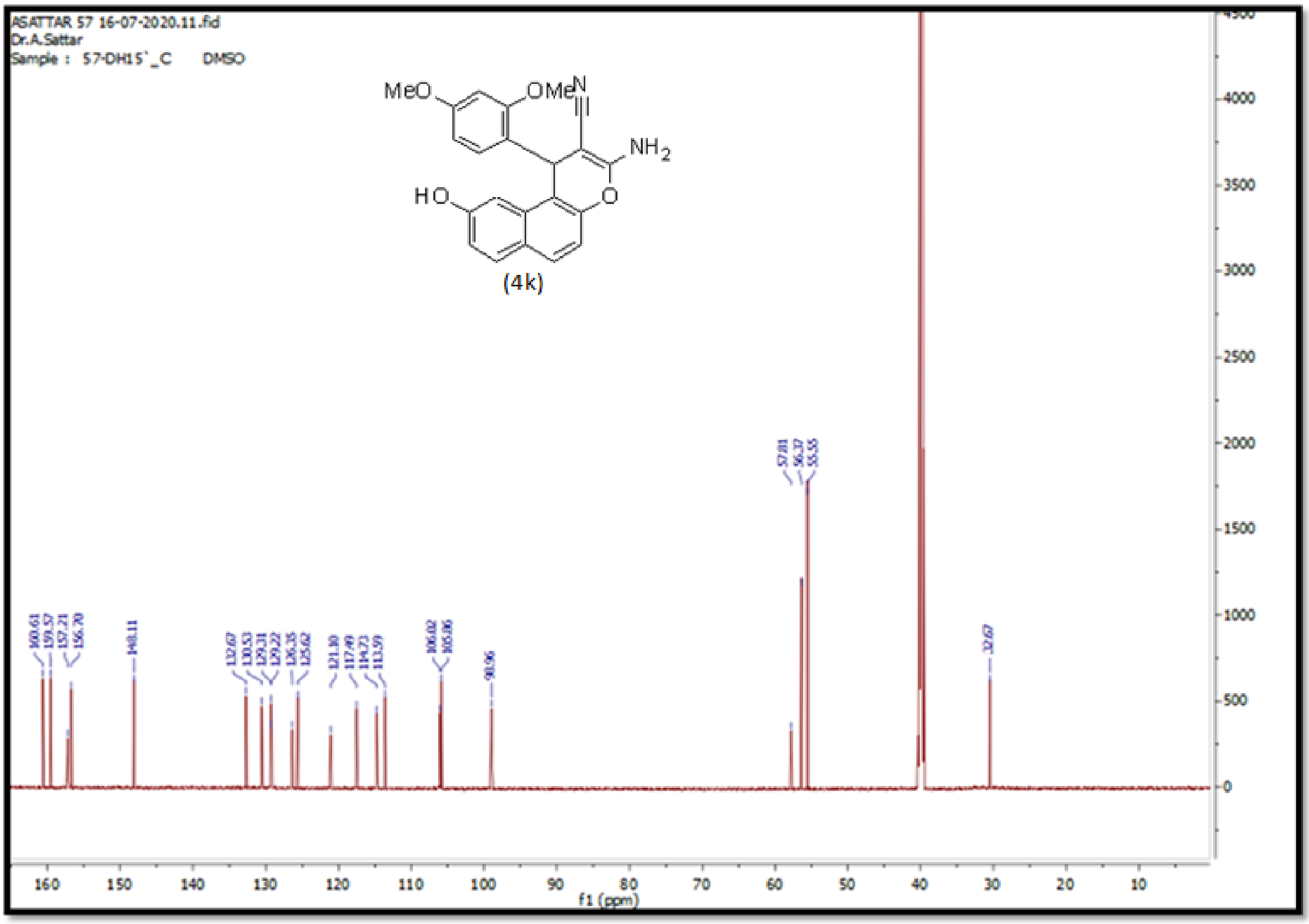

Figure S30: ^{13}C NMR of cpd. **(4k)**.

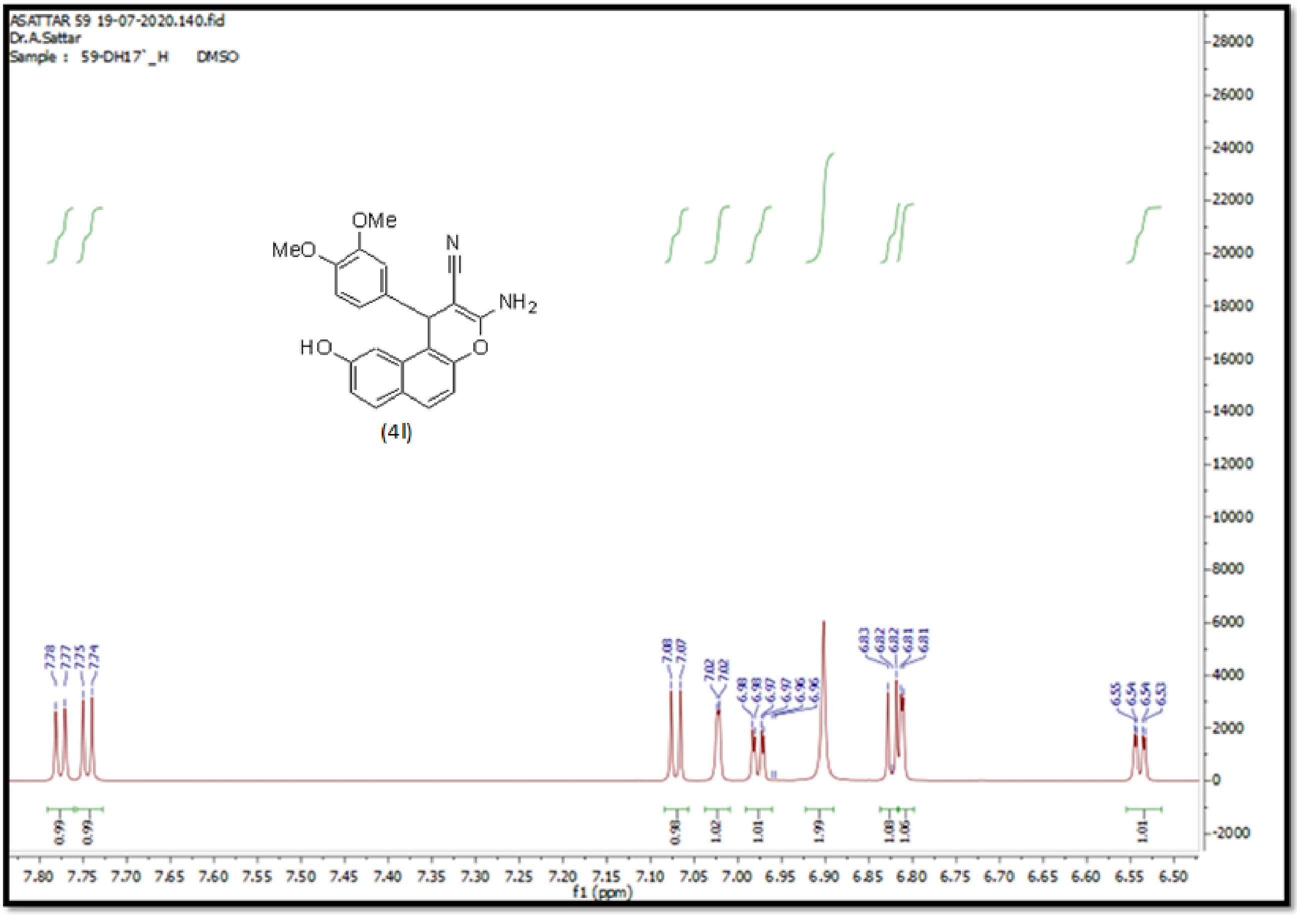

Figure S31: ^{1}H NMR 8.5-6.5 ppm of cpd. **(4l)**.

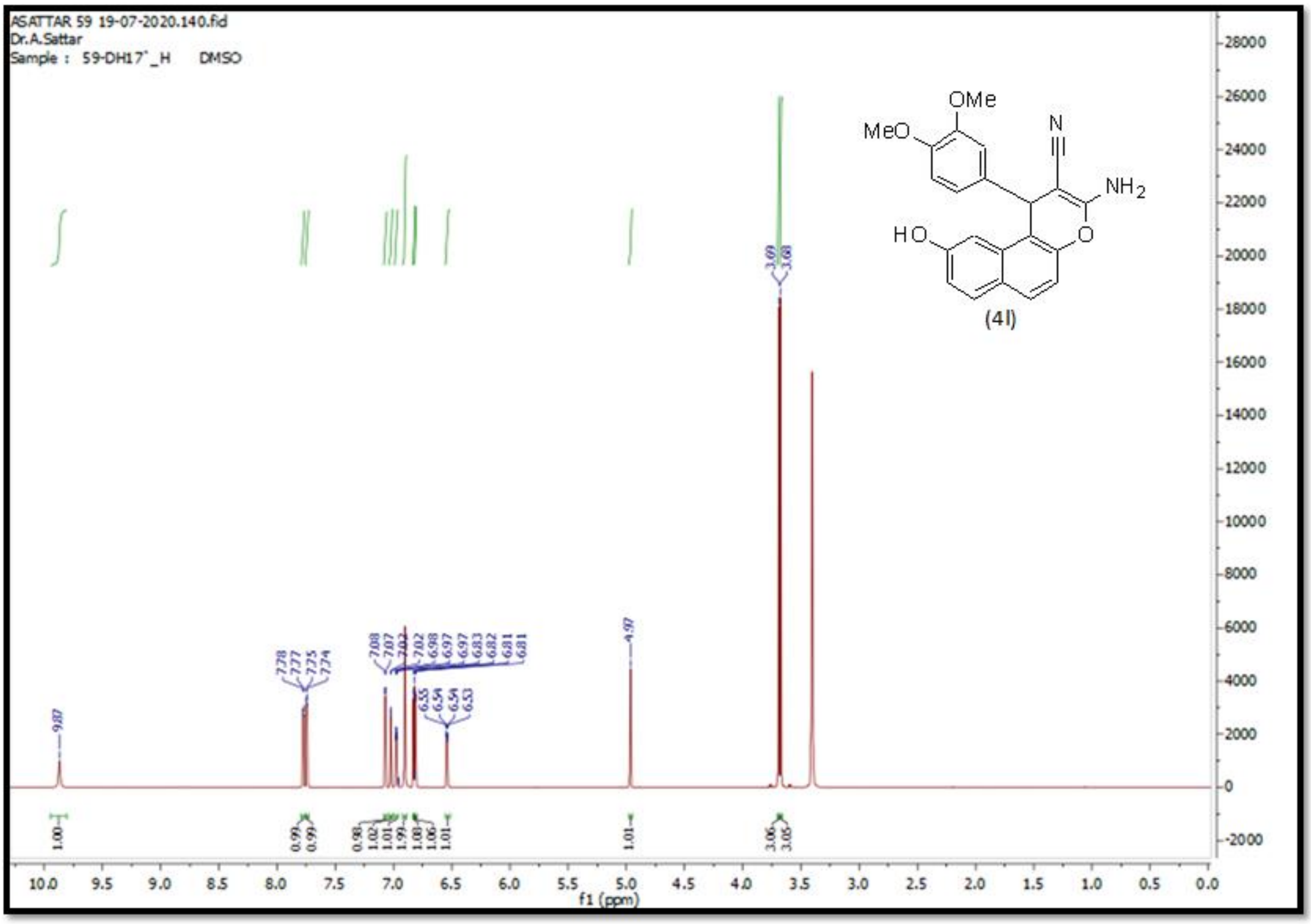

Figure S32: ^{1}H NMR of cpd. **(4l)**.

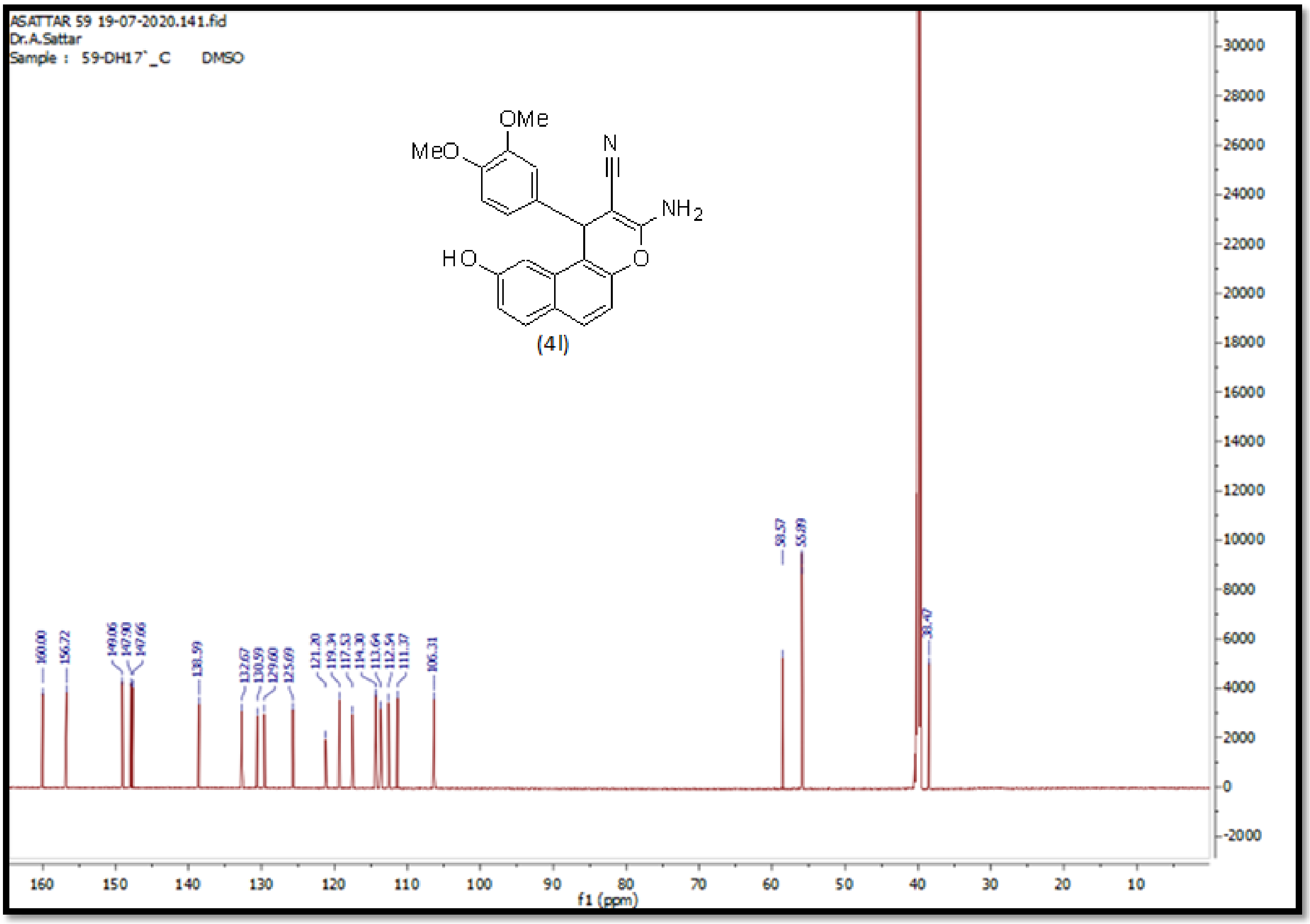

Figure S33: ^{13}C NMR of cpd. **(4l)**.

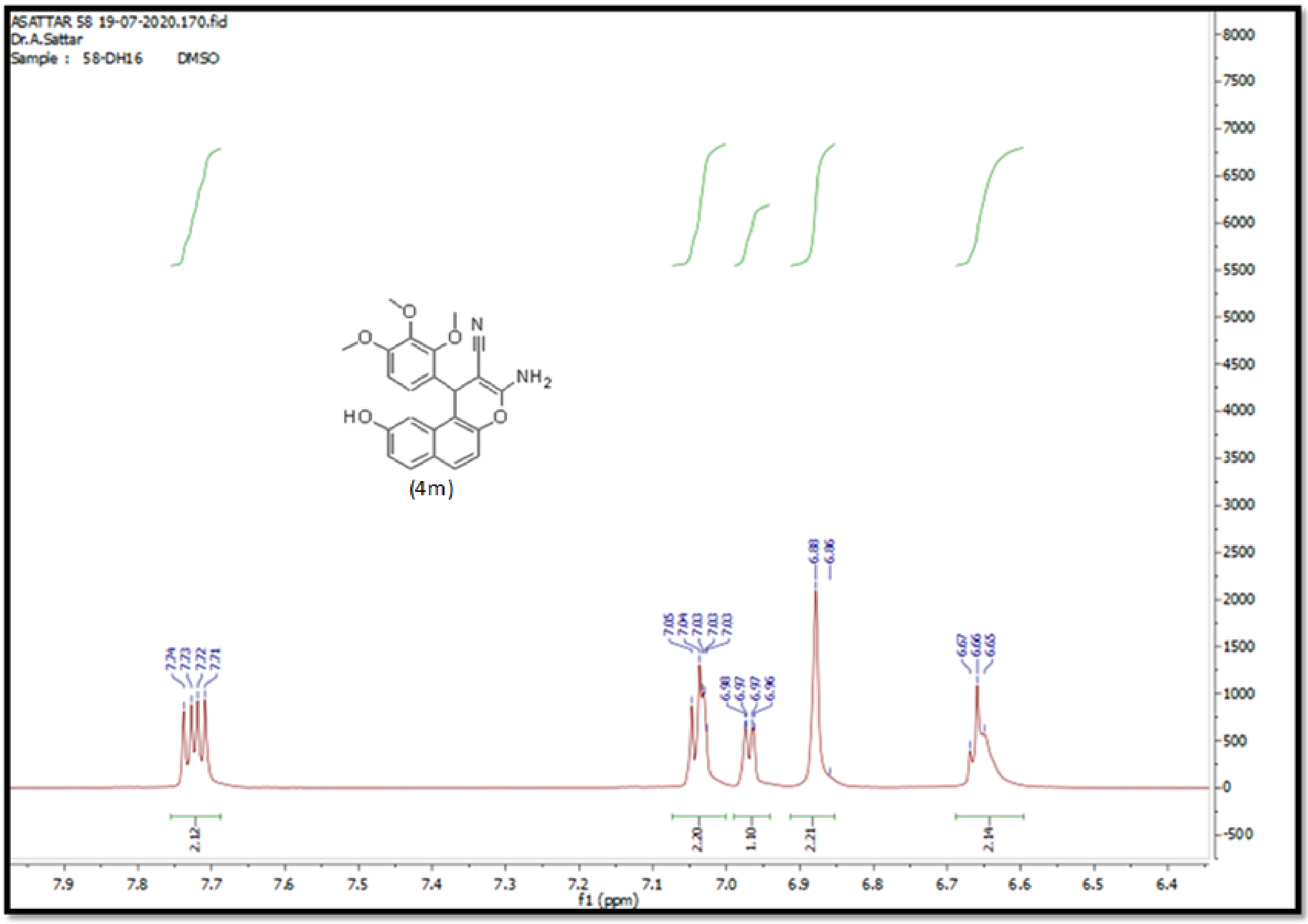

Figure S34: ^{1}H NMR 8.5-6.5 ppm of cpd. **(4m)**.

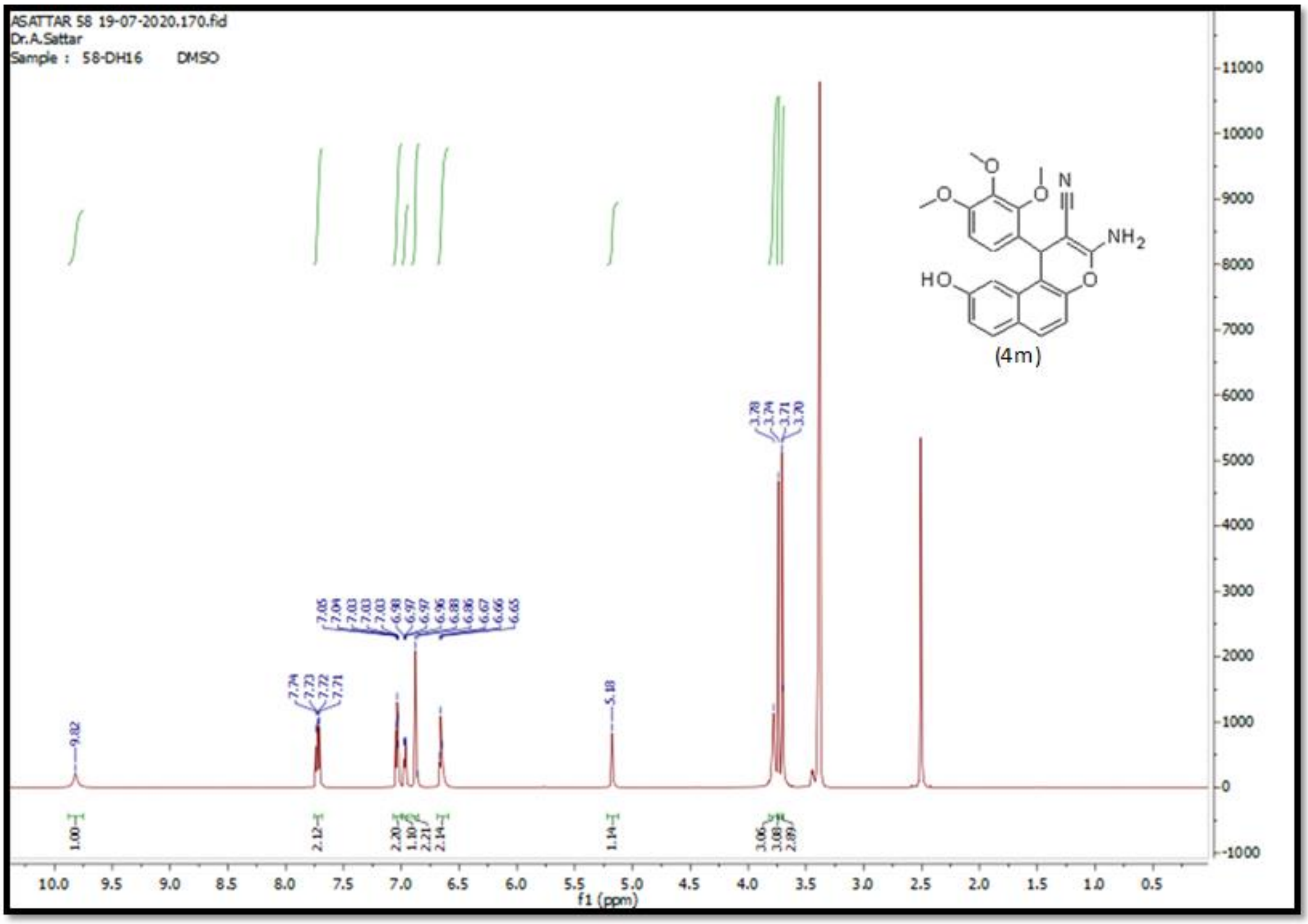

Figure S35: ^{1}H NMR of cpd. **(4m)**.

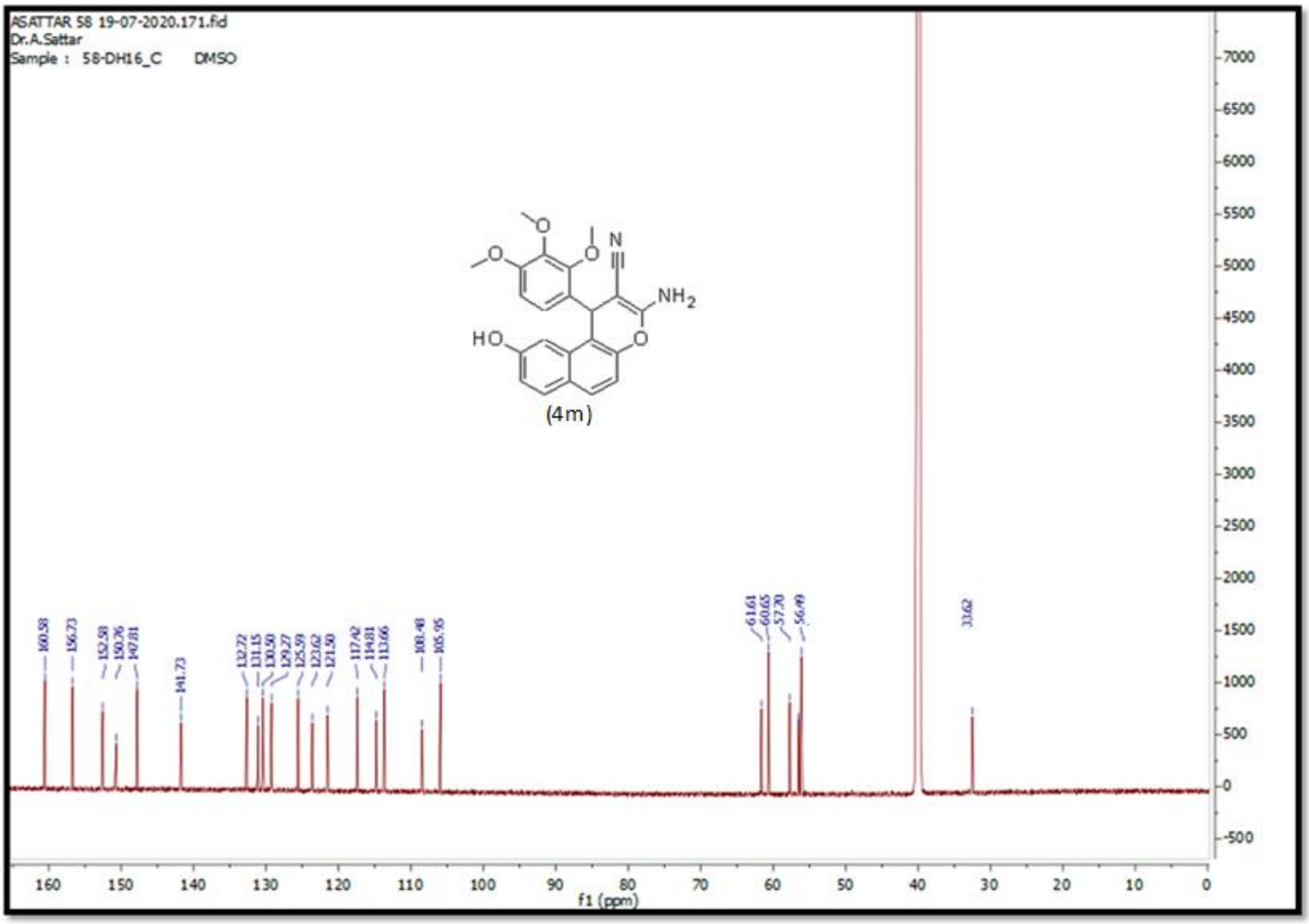

Figure S36: ^{13}C NMR of cpd. **(4m)**.

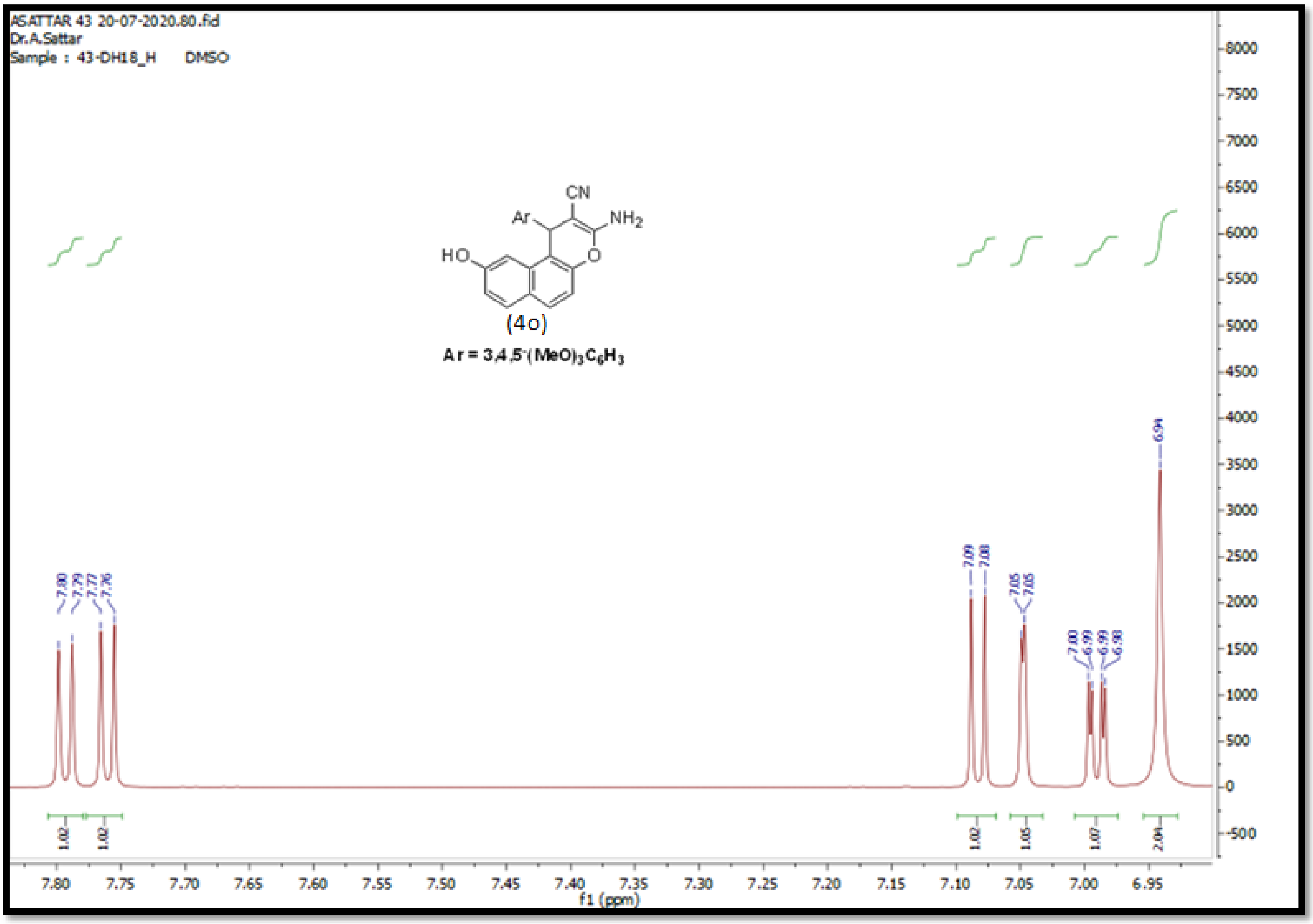

Figure S37: ^{1}H NMR 8.5-6.5 ppm of cpd. **(4n)**.

S41

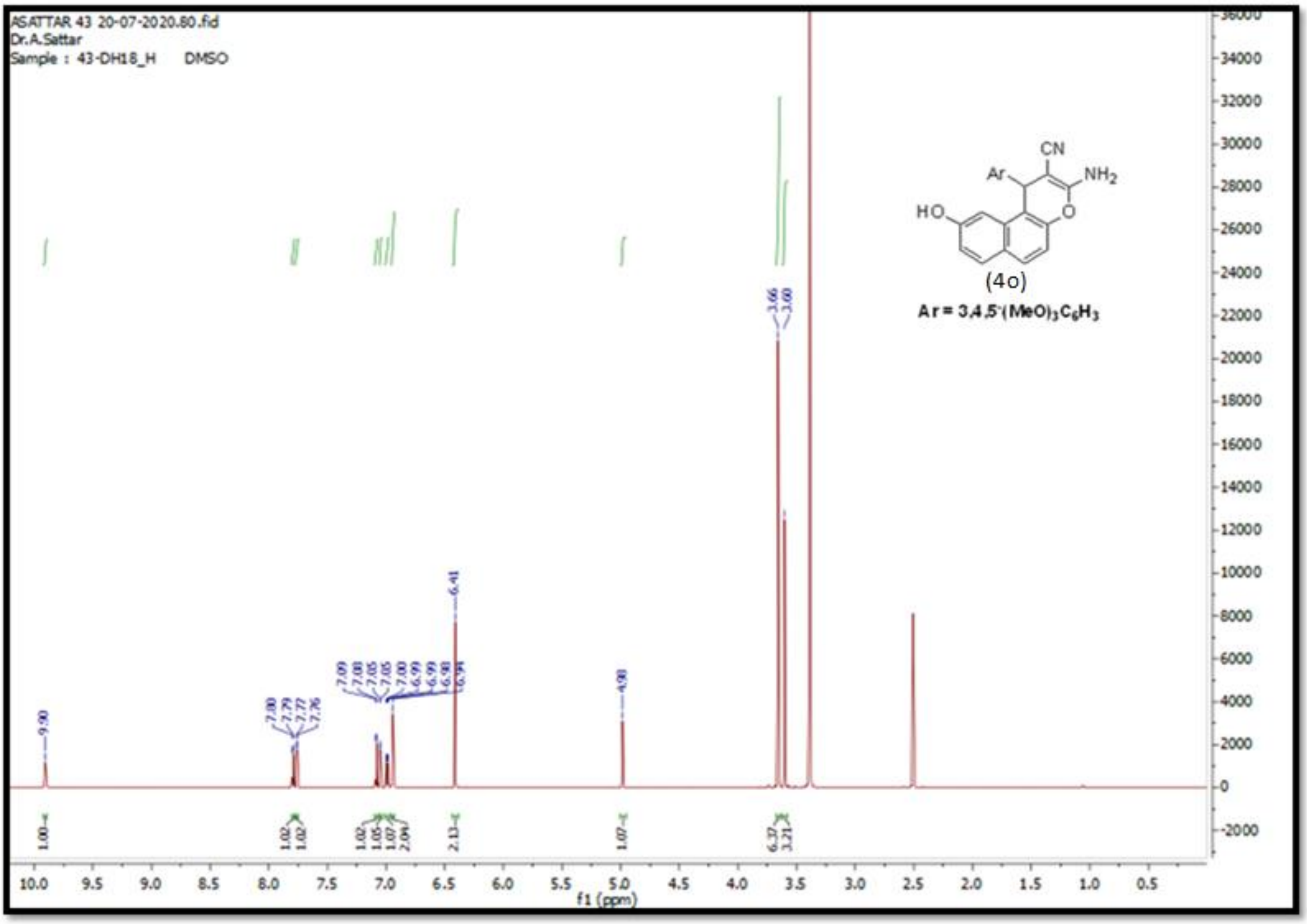

Figure S38: ^{1}H NMR of cpd. **(4n)**.

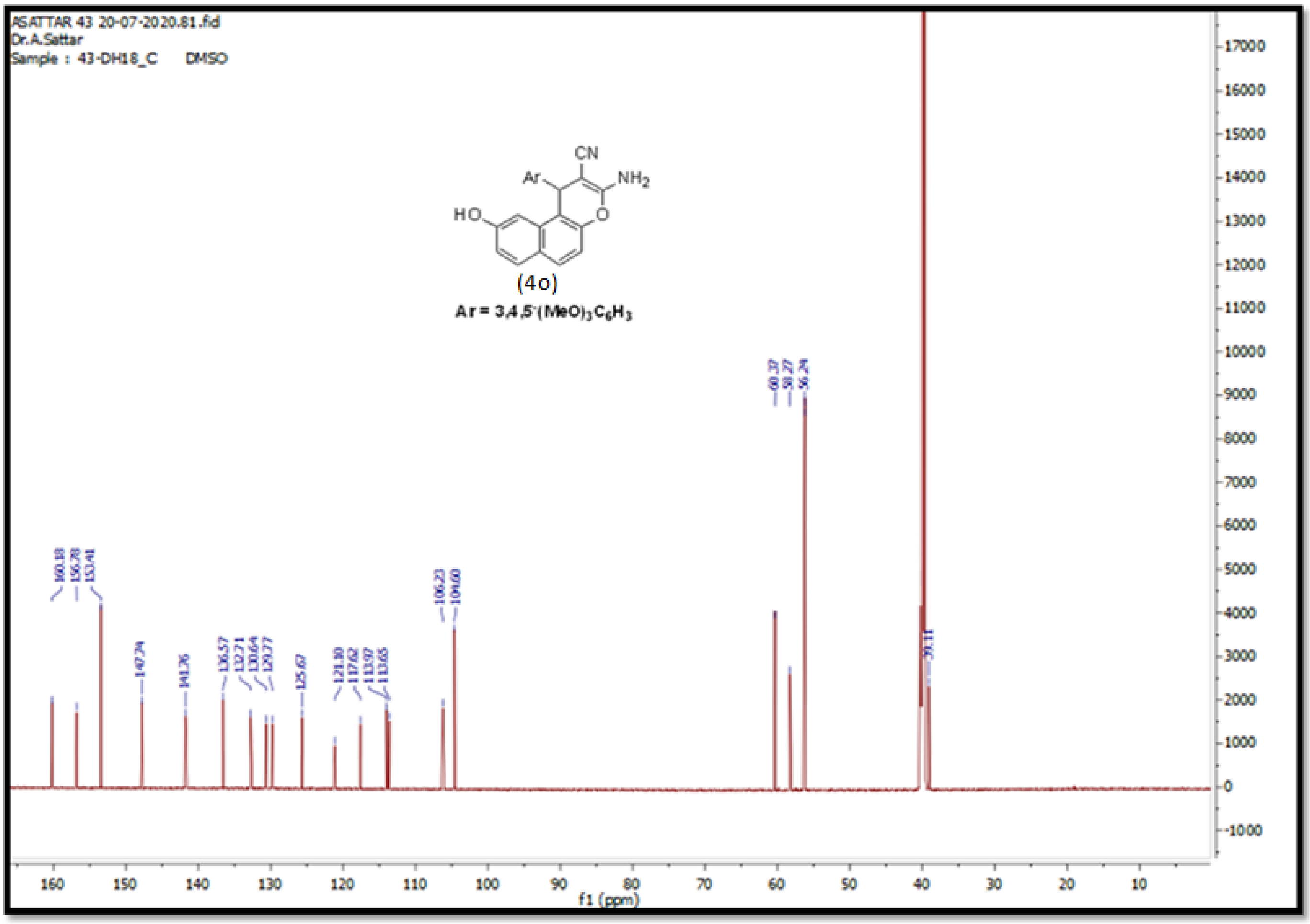

Figure S39: ^{13}C NMR of cpd. **(4n)**.

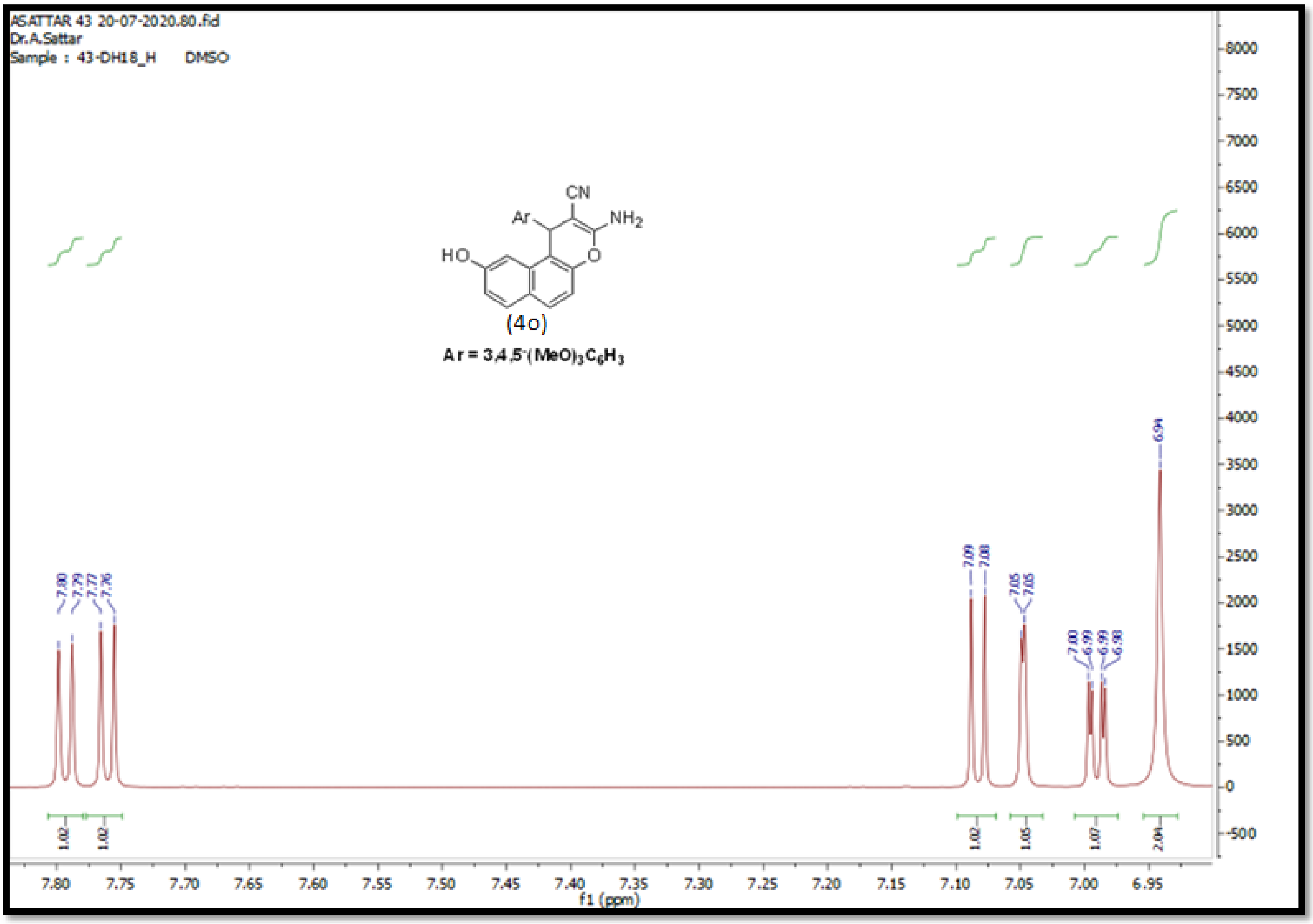

Figure S40: ^{1}H NMR 8.5-6.5 ppm of cpd. **(4o)**.

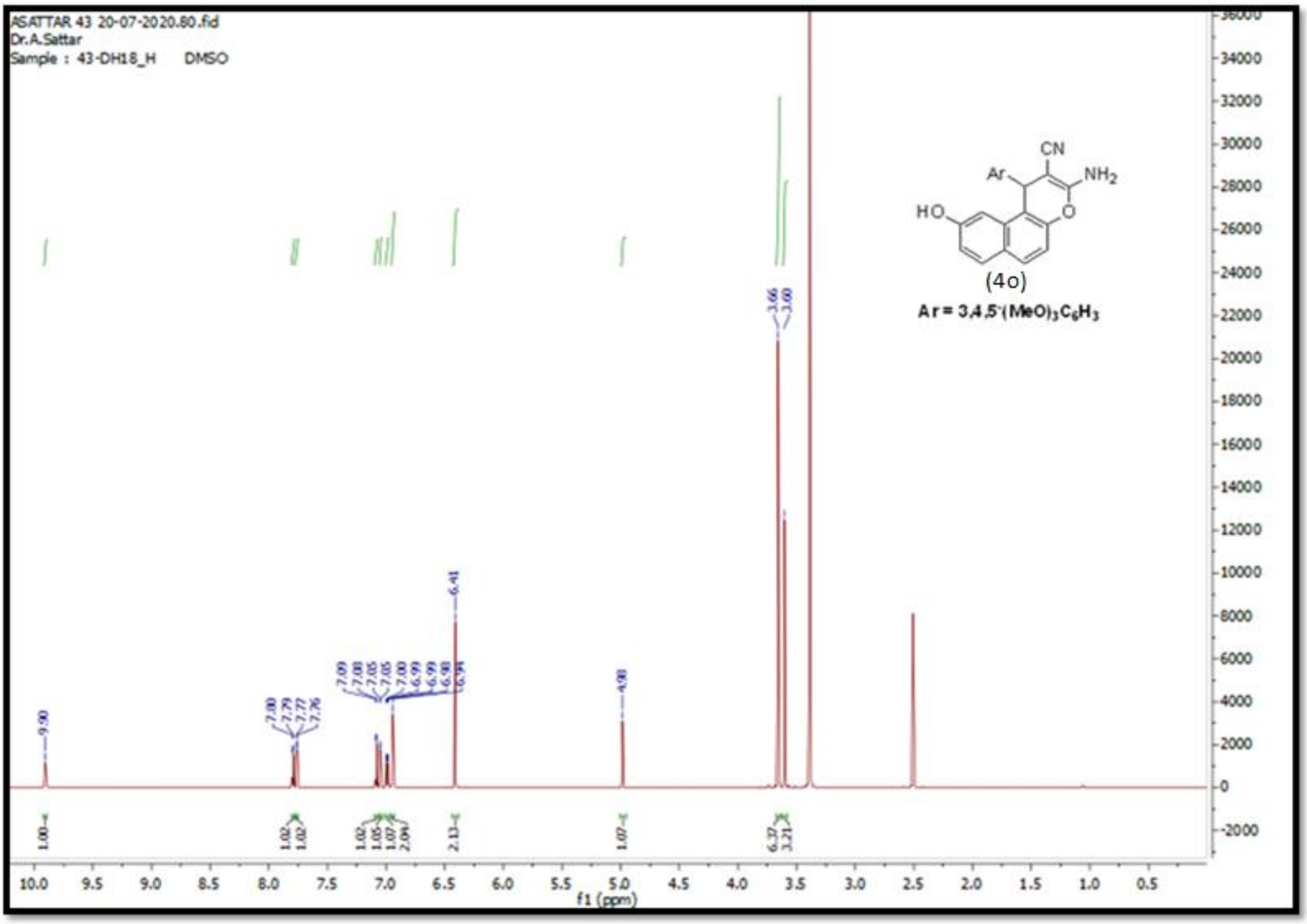

Figure S41: ^{1}H NMR of cpd. **(4o)**.

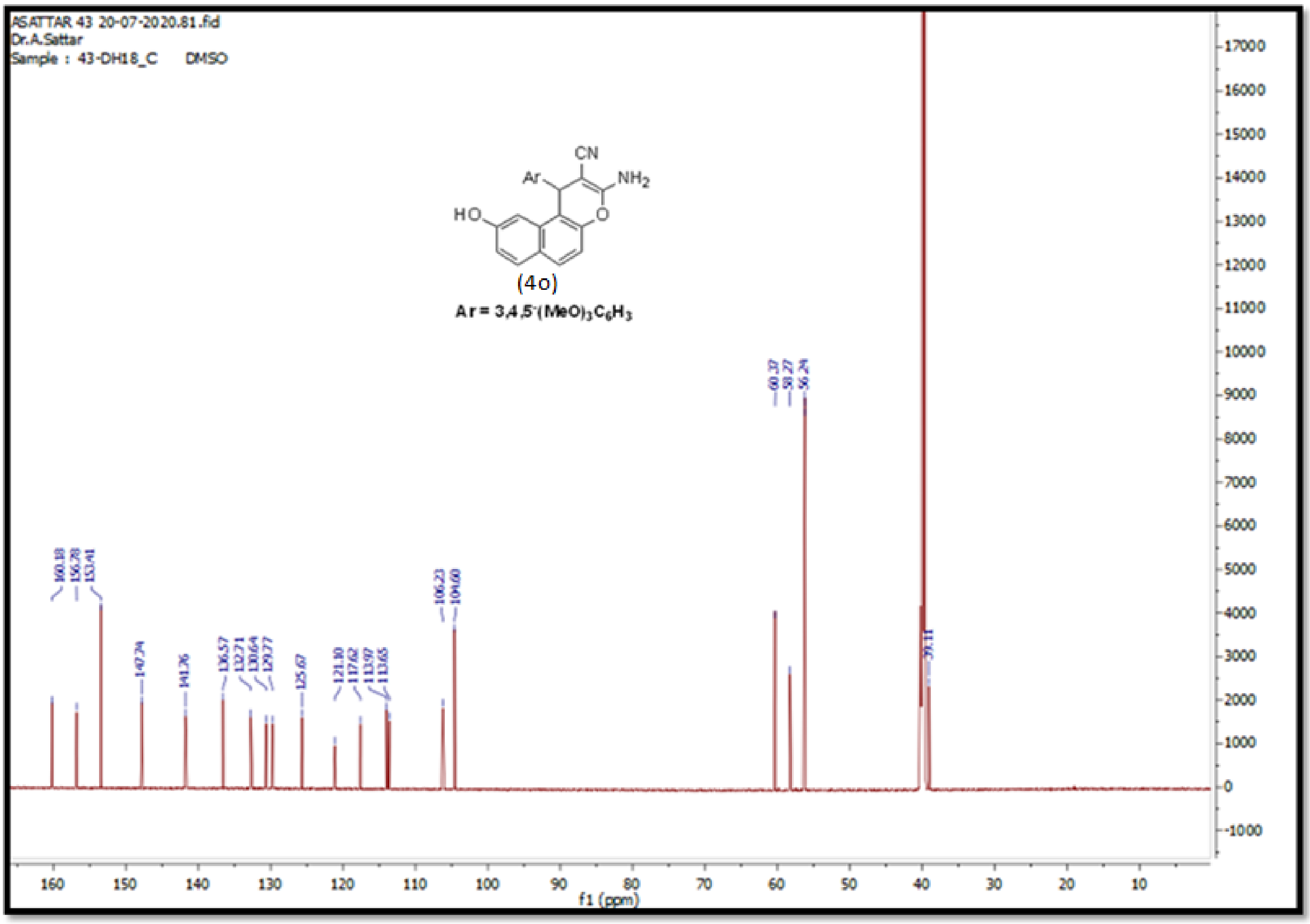

Figure S42: ^{13}C NMR of cpd. **(4o)**.

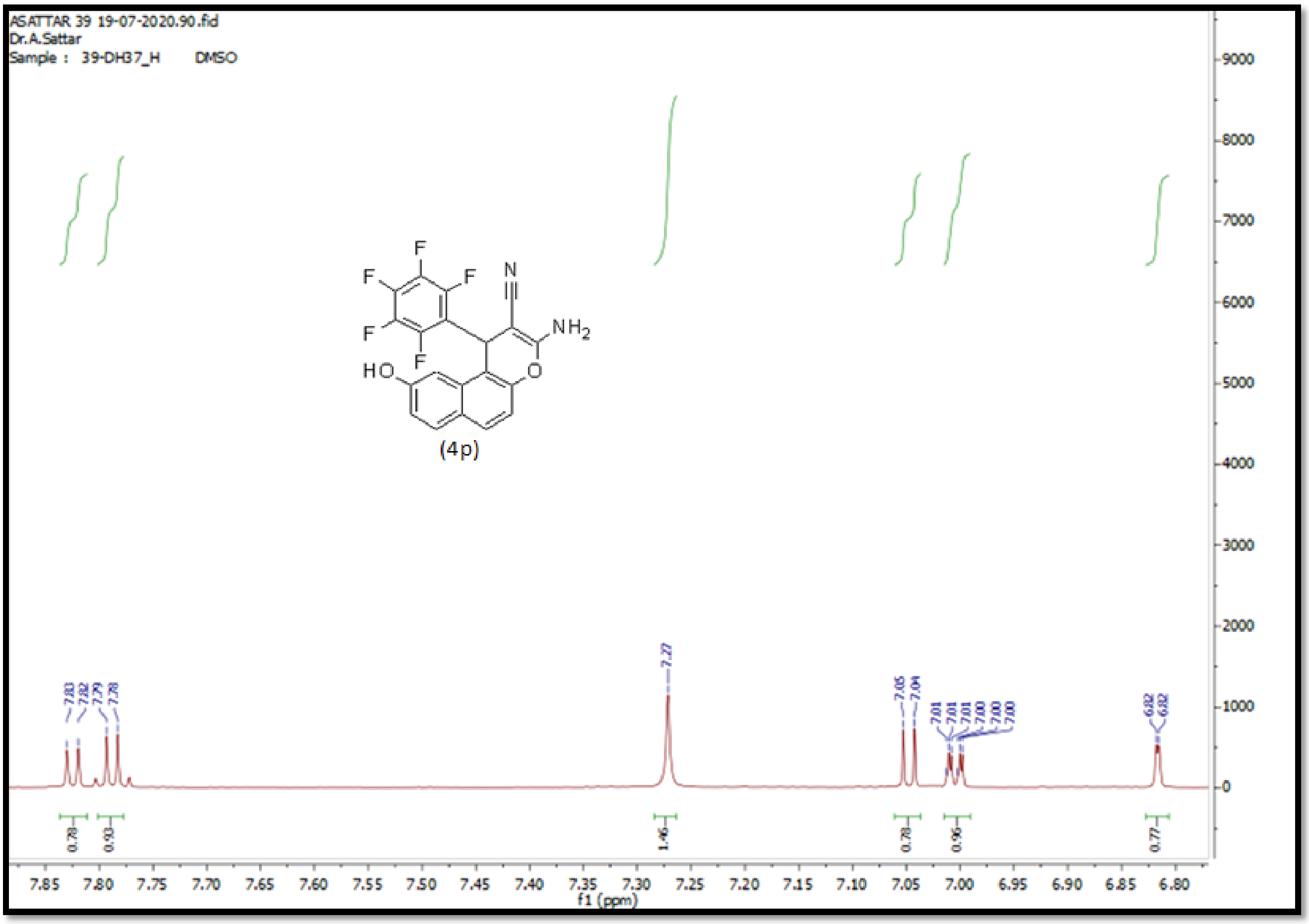

Figure S43: ^{1}H NMR 8.5-6.5 ppm of cpd. **(4p)**.

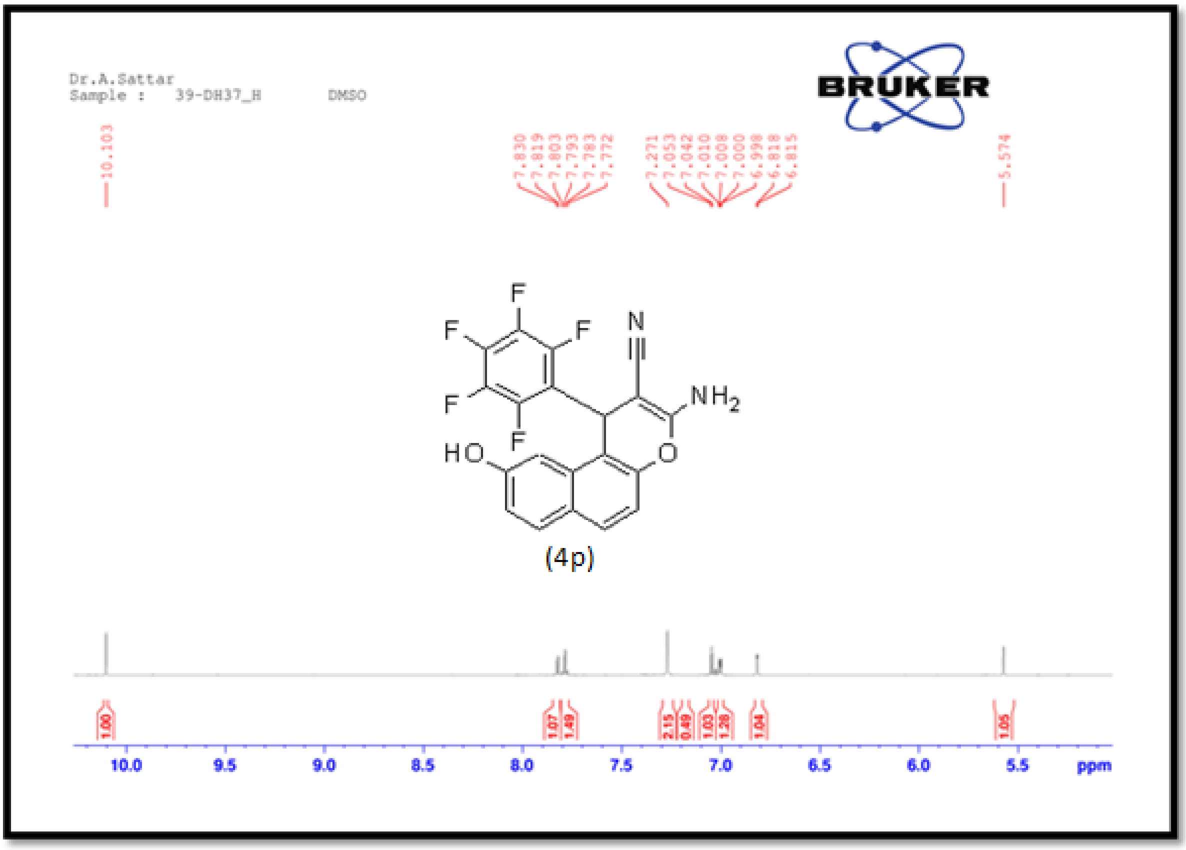

Figure S44: ^{1}H NMR 10.5-5.5 ppm of cpd. **(4p)**.

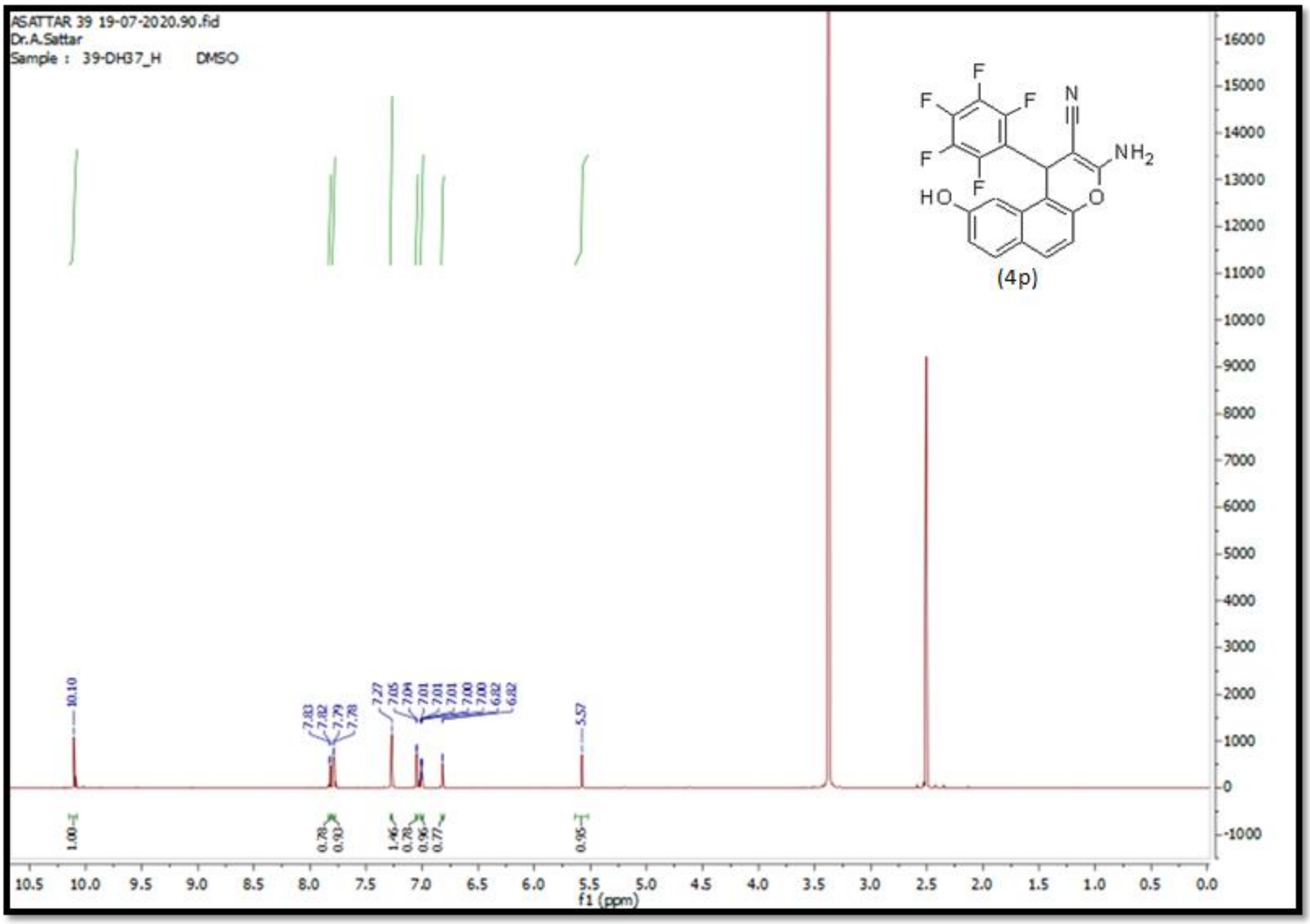

Figure S45: ^{1}H NMR of cpd. **(4p)**.

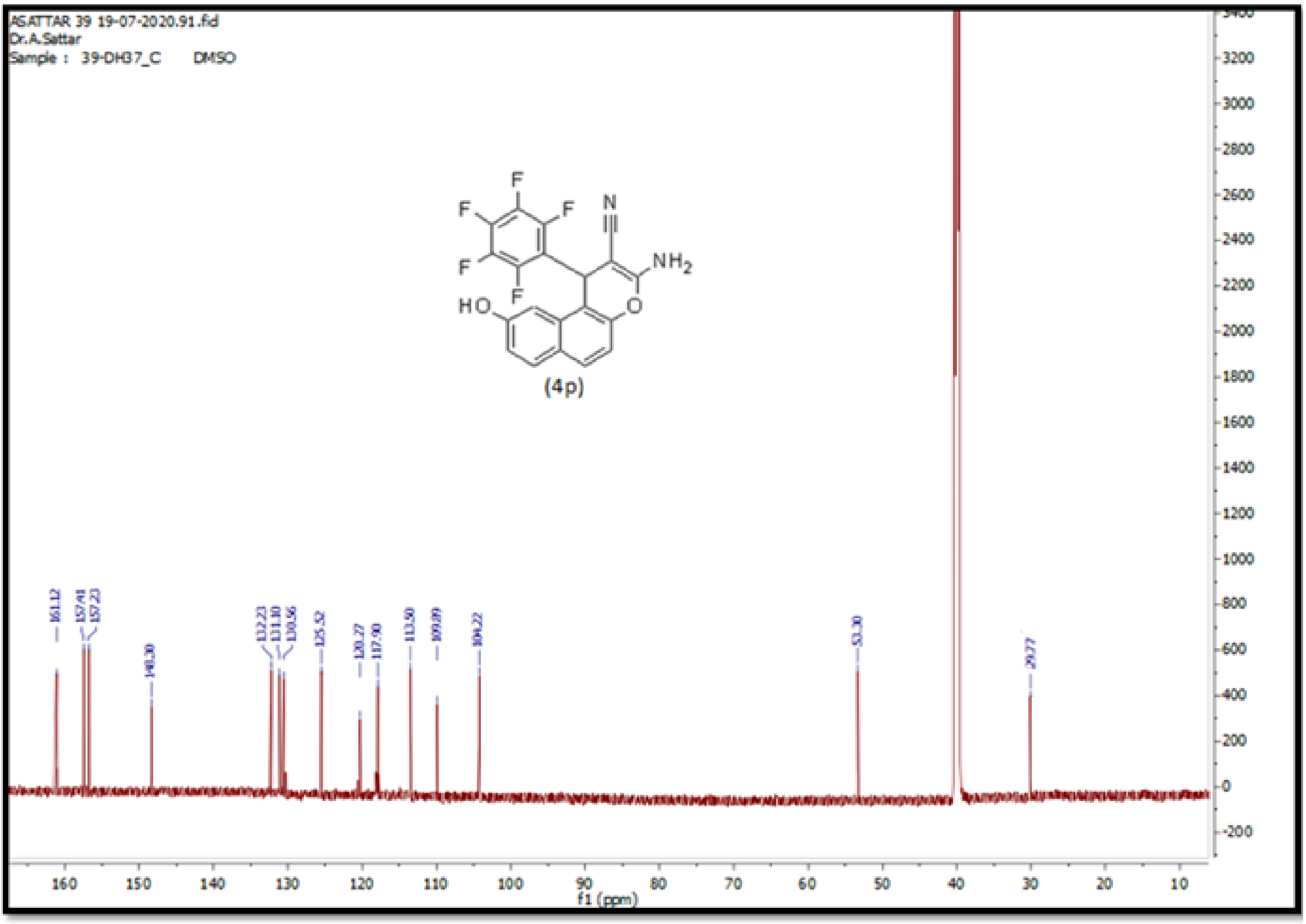

Figure S46: ^{13}C NMR of cpd. **(4p)**.

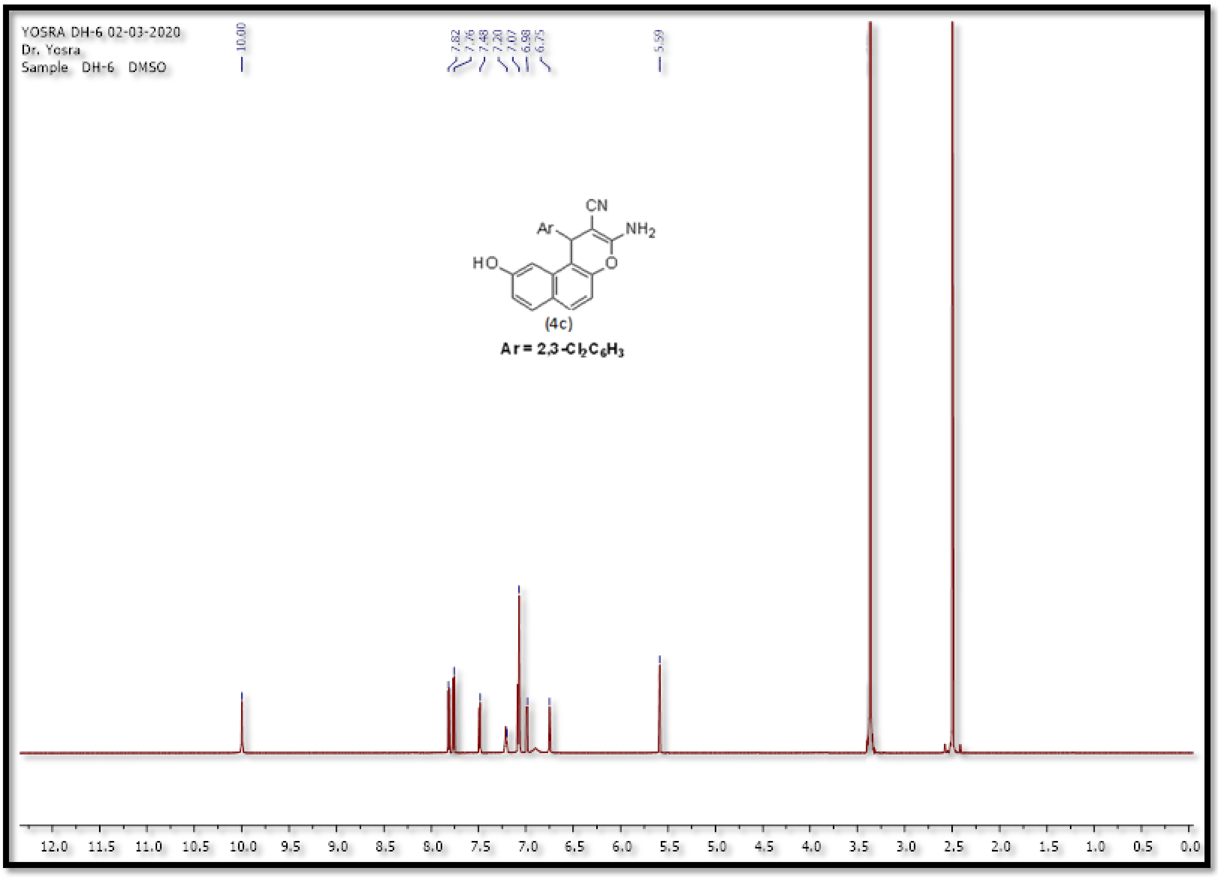

Figure S47: ^{1}H NMR of cpd. **(4c)**.

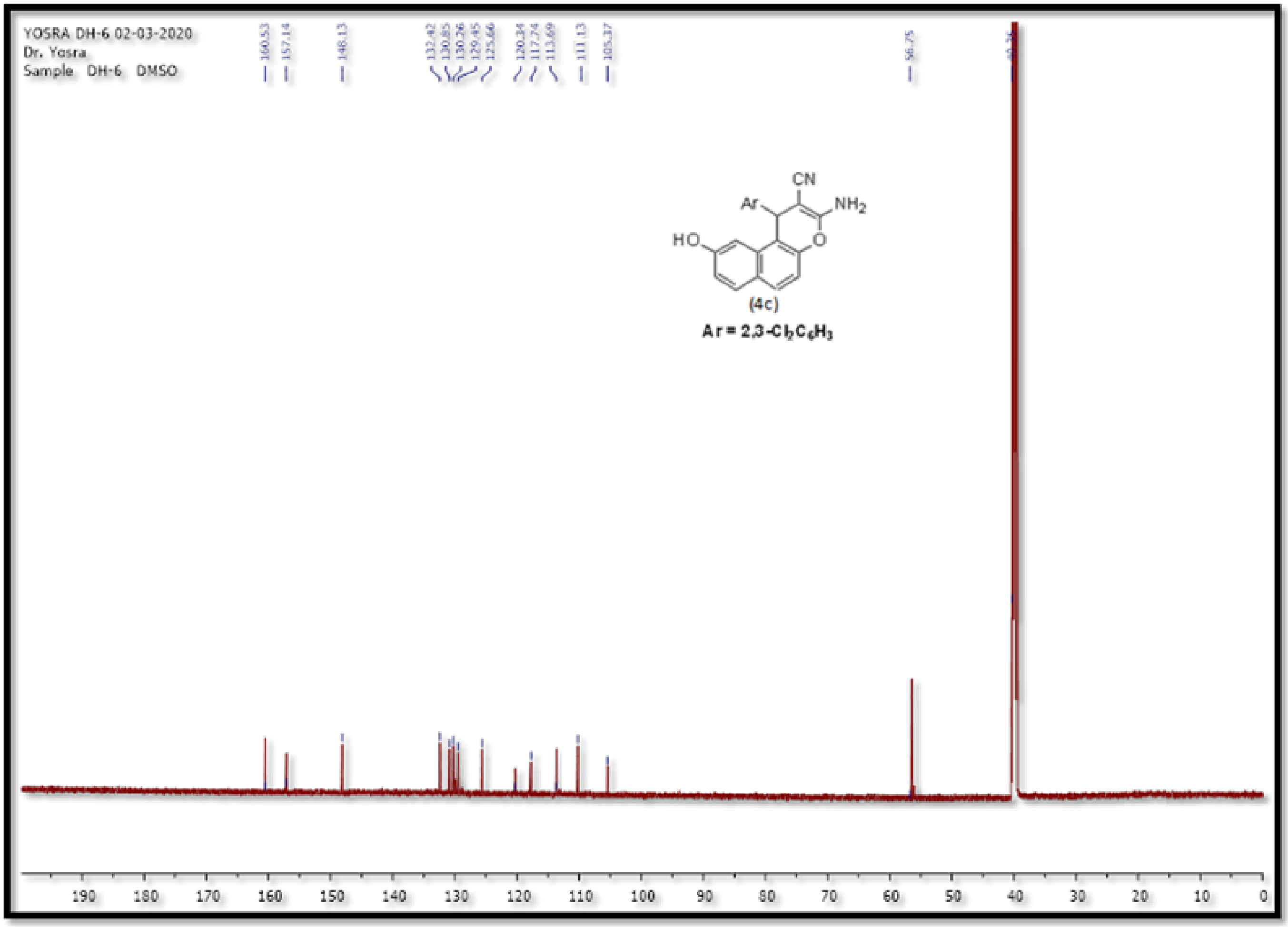

Figure S48: ^{13}C NMR of cpd. **(4c)**.

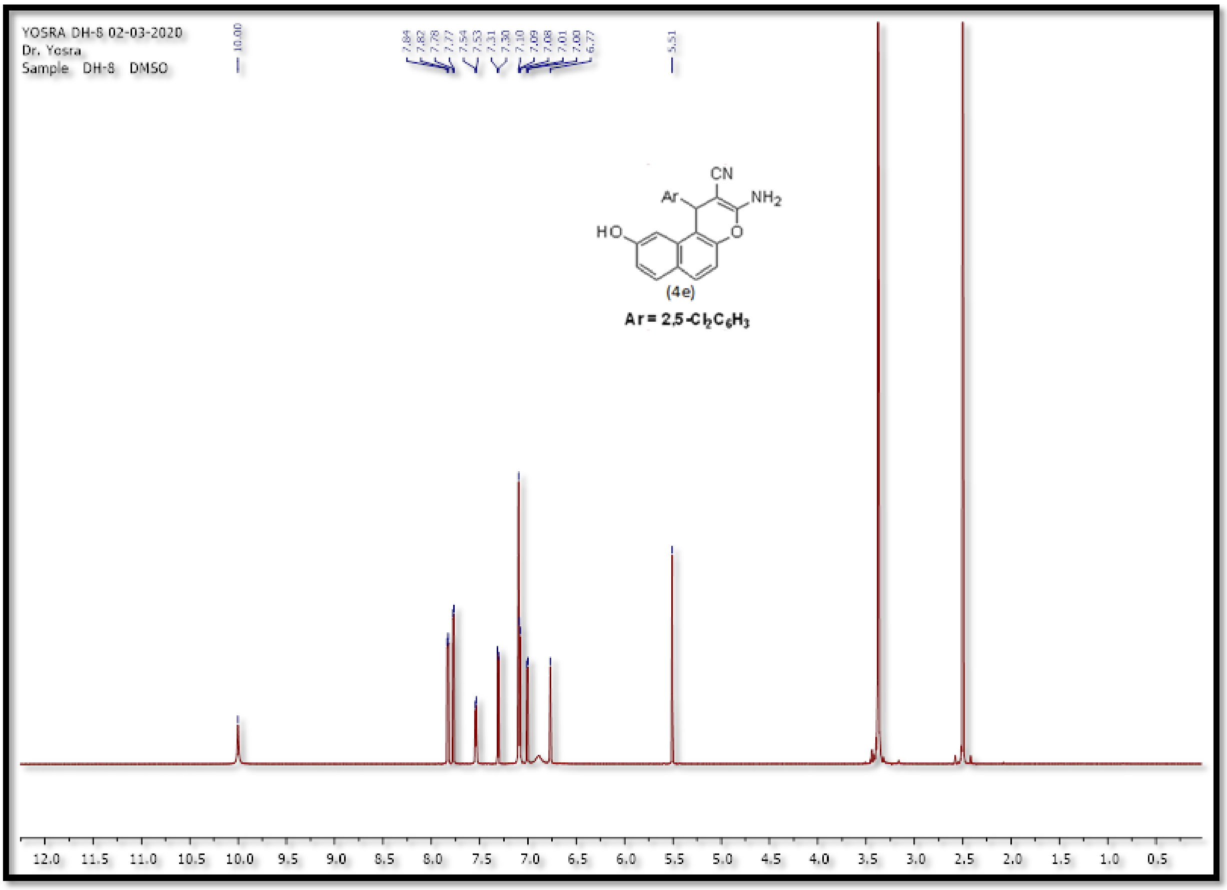

Figure S49: ^{1}H NMR of cpd. **(4e)**.

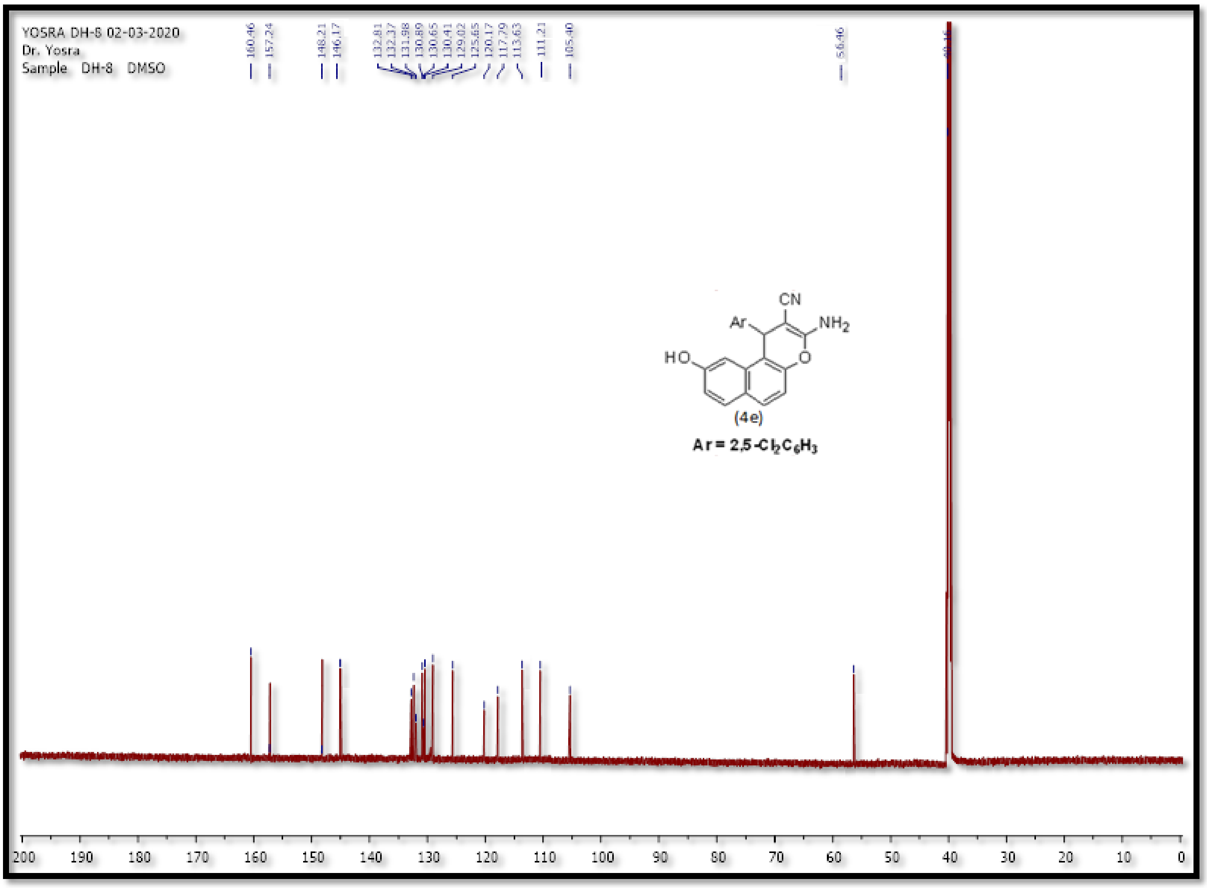

Figure S50: ^{13}C NMR of cpd. **(4e)**.

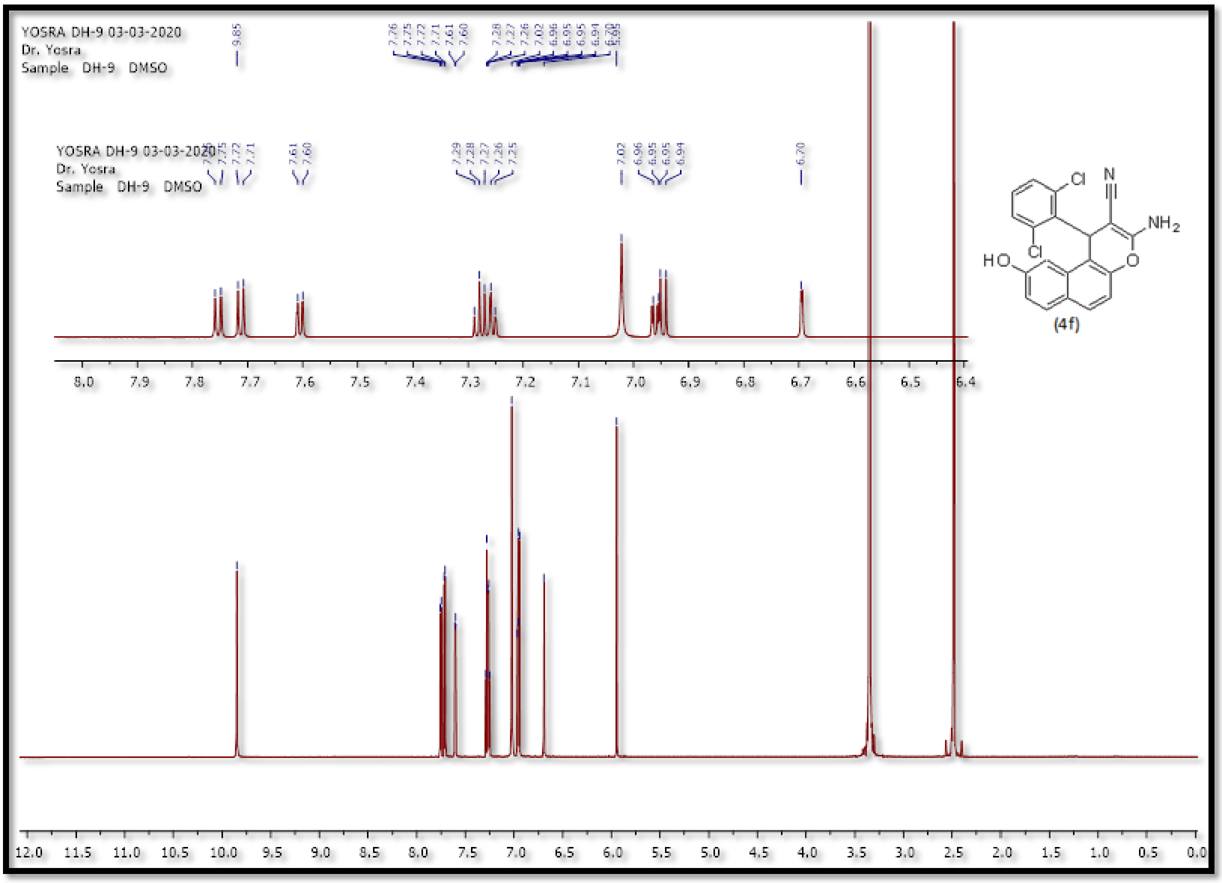

Figure S51: ^{1}H NMR of cpd. **(4f)**.

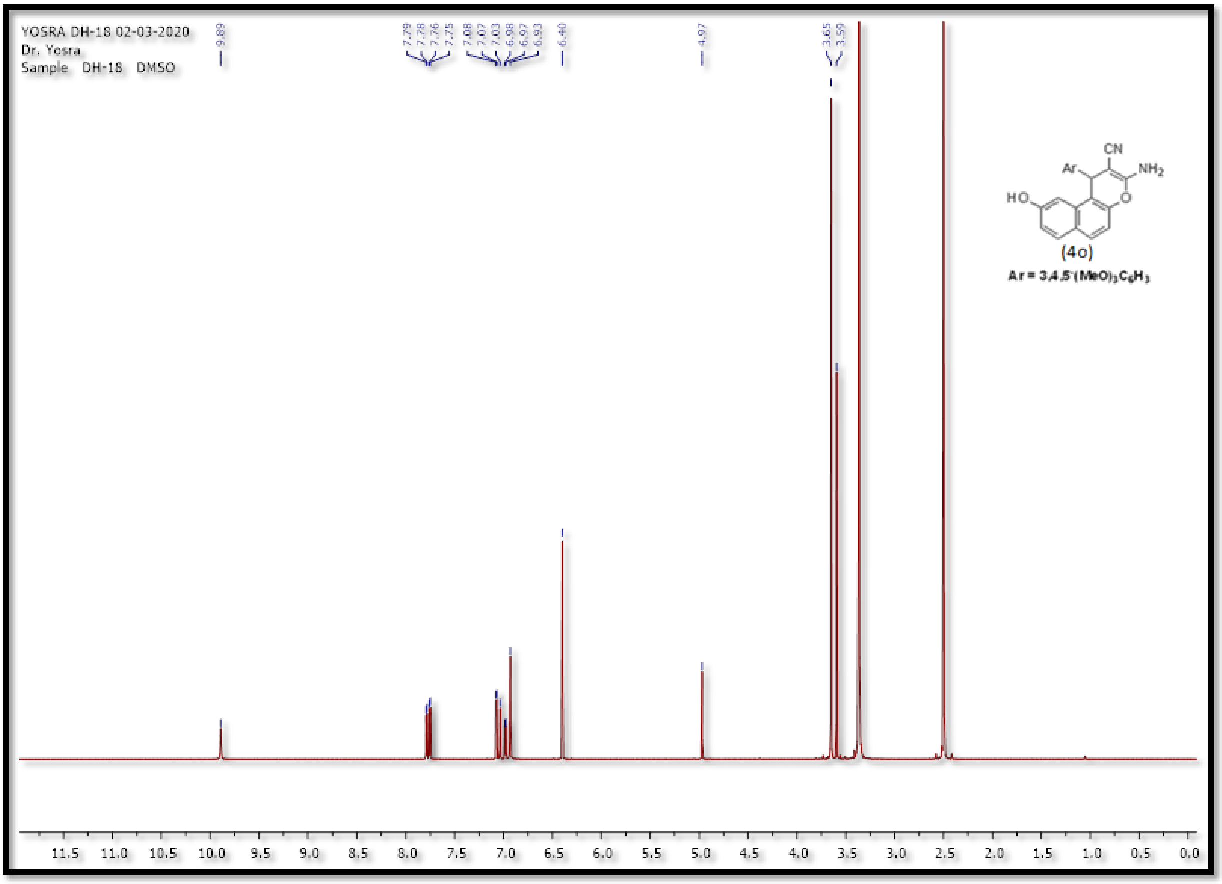

Figure S52: ^{1}H NMR of cpd. **(4o)**.

Table S1: The optimization Microwave irradiation condition for Synthesis of **4a-p**.

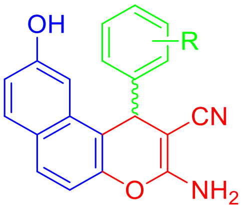

4a-p

Cpd.	R	yield (%)[a]	yield (%)[b]	yield (%)[c]
4a	2,4-F_2	56	75	88
4b	2,6-F_2	77	81	85
4c	2,3-Cl_2	76	83	89
4d	2,4-Cl_2	74	85	88
4e	2,5-Cl_2	79	81	84
4f	2,6-Cl_2	63	83	84
4g	3,4-Cl_2	66	76	83
4h	2-Cl-6-F	69	80	87
4i	3,5-Br_2	59	83	86
4j	2-HO-3-MeO	72	76	83
4k	2,4-$(MeO)_2$	62	84	90
4l	3,4-$(MeO)_2$	76	78	89
4m	2,3,4-$(MeO)_3$	78	80	90
4n	2,4,5-$(MeO)_3$	73	76	87
4o	3,4,5-$(MeO)_3$	64	80	89
4p	2,3,4,5,6-F_5	65	77	79

a: 200 W/ 1 min.; b: 300 W/ 1.5 min.; c: 400 W/2 min.
